# Sonosynthesis of new functionalized optically active triazines *via* double Mannich reaction: antibacterial potential and *in silico* docking study†

**DOI:** 10.1039/d5ra01283j

**Published:** 2025-06-04

**Authors:** Hajar A. Ali, Mohamed M. Hammouda, Mohamed A. Ismail, Eslam A. Ghaith

**Affiliations:** a Chemistry Department, Faculty of Science, Mansoura University El-Gomhoria Street Mansoura 35516 Egypt abdelghaffar@mans.edu.eg +2010244410784; b Department of Chemistry, College of Science and Humanities in Al-Kharj, Prince Sattam Bin Abdulaziz University Al-Kharj 11942 Saudi Arabia; c Chemistry Department, Faculty of Science, New Mansoura University New Mansoura City Egypt

## Abstract

In this study, we employed both conventional and ultrasound irradiation approaches to fabricate a library of ten new triazine hybrids by the divergent double-Mannich reaction. The titled compounds were characterized by extensive spectral analyses, including IR, MS, 1D NMR (1H, ^13^C, ^15^N, ^19^F), and 2D NMR (DEPT135, HSQC). Additionally, their antibacterial activity against a spectrum of bacterial strains, encompassing two Gram-positive and two Gram-negative bacteria, was assessed. Notably, compound 9 emerged as the most potent antibacterial agent with an inhibition zone of 39 mm against *Bacillus subtilis*, 50 mm against *Staphylococcus epidermidis*, 41 mm against *Enterobacter cloacae*, and 40 mm against *Escherichia coli*. Moreover, molecular docking simulation demonstrated the responsible binding affinity of the synthesized scaffolds with target proteins (PDB: 1OF0, 8P20, 1KQB, 1KZN). Likewise, structure–activity relationship (SAR) studies of the newly synthesized triazine derivatives demonstrated that substituent identity significantly impacts antibacterial activity. Notably, compound 9, bearing chloro and fluoro atoms, exhibited the highest antibacterial activity among all tested derivatives. Furthermore, optical activity measurements were performed through a polarimeter to confirm the tetrahedral stereocenter of nitrogenous scaffolds.

## Abstract 2

In this study, we employed both conventional and ultrasound irradiation approaches to fabricate a library of ten new triazine hybrids by the divergent double-Mannich reaction.

## Graphical abstract

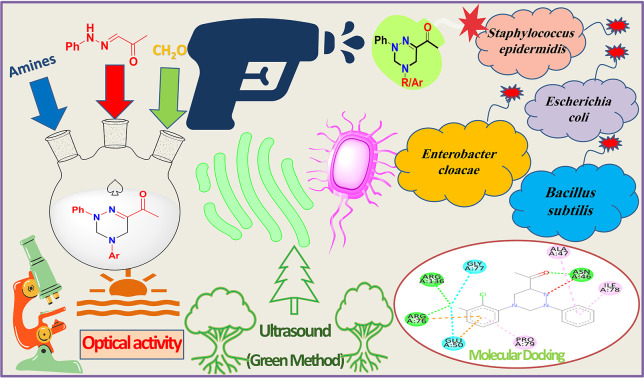

## 1 Introduction

Recently, the incidence of microbial infections and the emergence of resistance to current drugs have posed significant threats to human health, as one of the leading causes of death around the world.^1–4^ The etiology of this pathological phenomenon poses a formidable challenge in clinical studies, which can be attributed to the potential impacts of microbial infections on mortality and morbidity, causing serious fear and inconvenience. On the other hand, many reports expect that fatalities due to infections from multi-drug resistant (MDR) bacteria will exceed those from cancer by 2050 due to the long turnaround time and the limited efficacy of existing antibiotics against MDR.^5–8^ If no restricted measures are implemented, the annual death toll is projected to reach 10 million by 2050.^9^ In the same context, various spanning families of MDR pathogens were listed in the WHO's Bacterial Priority Pathogens List (WHO BPPL) for the year 2024, due to their concerns about treatability and transmissibility.^10^ As a result, it is imperative to synthesize and develop new antibacterial agents to overcome antibacterial resistance mechanisms that have eluded contemporary therapies due to their accelerated activities.^11–14^

**Fig. 1:**
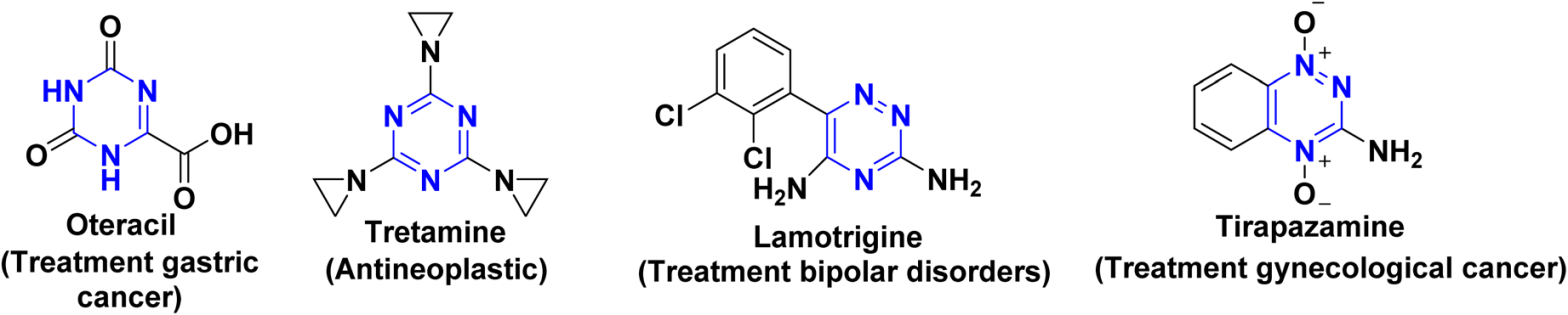
Some marketed drugs containing triazine molecules.

On the other hand, nitrogenous heterocyclic scaffolds are the basic moieties in various bioactive natural products and approved purchasable antibiotics such as penicillin, levofloxacin, and cefalexin. According to recent reports, approximately 60–75% of small molecules drugs approved by FDA are nitrogenous heterocyclic systems^2,15^ Among these scaffolds are triazine and its diverse derivatives, as they have a track record of medicinal applications such as antibacterial,^16^ antimicrobial,^17^ antiviral,^18–20^ antiulcer,^17^ anti-inflammatory,^21^ anticonvulsant,^22^ pyruvate dehydrogenase kinase inhibitors,^23^ and herbicidal agents.^24–26^ Additionally, some triazine molecules are omnipresent in medicinally active compounds and marketed drugs such as oteracil,^27^ tretamine,^28^ lamotrigine,^29^ and tirapazamine (Fig. 1).^30^

Based on these considerations and aiming at combating drug-resistant infections, we synthesized new triazines as central cores linked to various bioactive hybrids. Also, these synthesized hybrids were evaluated as antibacterial agents against four bacterial strains. Eventually, the results were analyzed and validated in light of the molecular docking, confirming an extensive approach to the experimental and theoretical results.

**Fig. 2:**
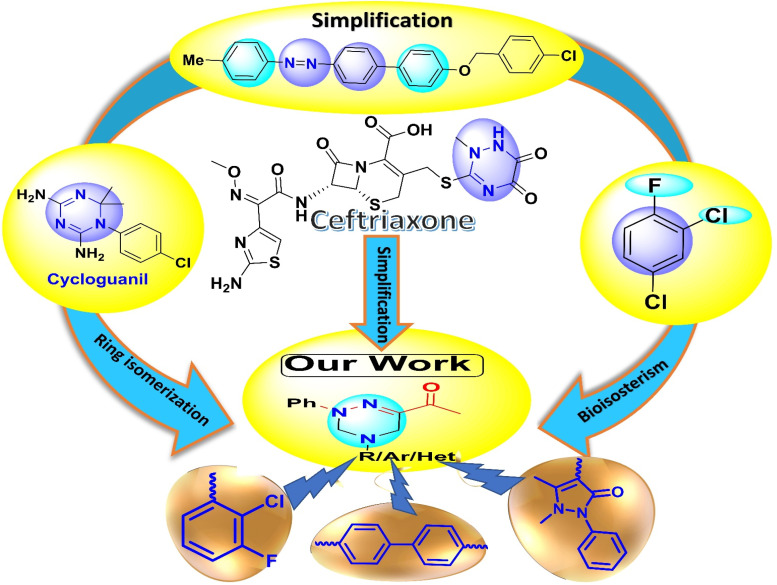
Rational design of triazine compounds for antibacterial activities.

A rational design was employed to investigate and develop new antibacterial agents, leveraging established evidence that scaffolds such as azabiphenyl,^31–33^ difluoro-chlorophenyl rings,^34^ and both 1,2,4- and 1,3,5-triazine hybrids^35,36^ could exhibit potent *in vitro* antibacterial activities. In this study, our objective is to synthesize a series of lead triazine scaffolds incorporating fluoro-chlorophenyl, azabiphenyl, benzidine, diphenylamine, tryptamine, and antipyrine as bioactive moieties, and subsequently evaluate their antibacterial activities (Fig. 2).

## 2 Results & discussion

**Scheme 1:**
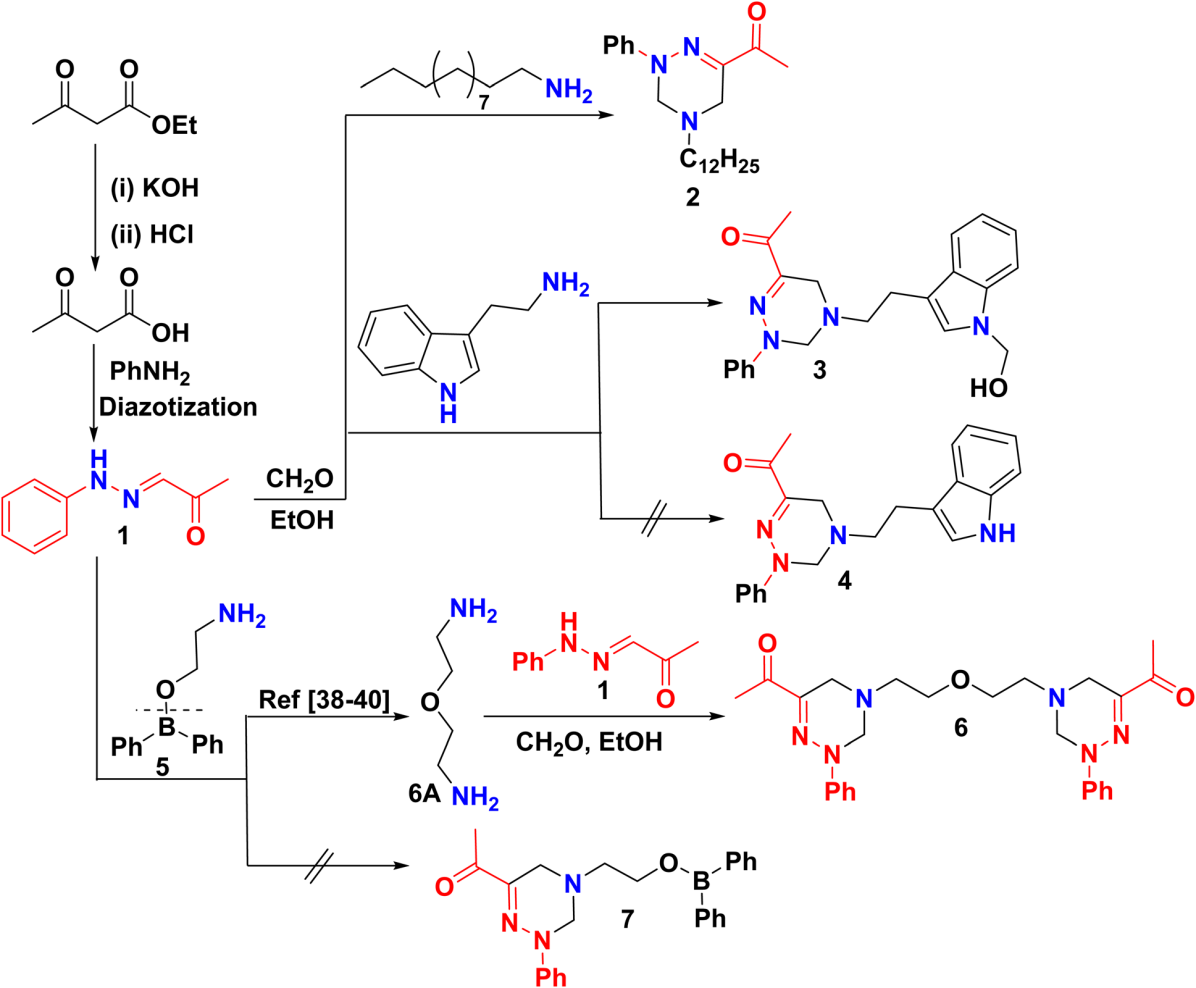
Synthesis of new 1,2,4-triazine derivatives.

Herein, an optimized approach was achieved to design and synthesize novel triazine derivatives incorporating numerous aliphatic, aromatic, and heteroaromatic entities through a straightforward multicomponent Mannich reaction. As the key starting material 1, was synthesized according to previously reported methodology^37^*via* diazotization reaction of aniline with NaNO_2_ and HCl at 0 °C, then coupling the diazonium salt with ethyl acetoacetate afforded (phenylhydrazineylidene)propan-2-one 1 (Scheme 1). Scaffold 1 was elucidated through the ^1^HNMR spectrum that revealed the presence of NH at *δ* = 11.33 ppm, and the ^13^CNMR of the carbonyl group of 1 appeared at *δ* = 196.84 ppm.

Multicomponent reactions (MCRs) of 1 with dodecyl amine as aliphatic amine and formalin (CH_2_O) in refluxing EtOH on a water bath *via* double Mannich reaction afforded 1-(4-dodecyl-2-phenyl-tetrahydro-1,2,4-triazin-6-yl)ethan-1-one 2 (Scheme 1). The structure of skeleton 2 was confirmed through its ^1^HNMR spectrum displaying three singlet signals at *δ* = 2.34, 3.49, 4.56 ppm for the methyl of the acetyl group and two deshielded methylene groups of the triazine moiety. Besides, two triplet signals at *δ* = 0.86 and 2.41 ppm correspond to dodecyl protons. Also, the ^13^C NMR spectrum of skeleton 2 exhibited characteristic dodecyl aliphatic carbon signals at *δ* = 14.44 to 31.77 and 53.33 ppm, in addition to the aliphatic carbons of triazine moiety at *δ* 45.65 and 64.60 ppm. The mass spectrometry of compound 2 displayed a molecular ion peak (*m*/*z*) = 371.14 attributed to C_23_H_37_N_3_O. Analogous to compound 2, treatment of 1 with tryptamine in EtOH yielded the crude product of compound 3 instead of the desired product 4 (Scheme 1). The residue was purified using preparative silica gel chromatography to yield a sol product identified as ((hydroxymethyl)-indol-tetrahydro-triazin-6-yl)ethanone 3. The structure of constitution 3 seemed to be consistent with its spectral data as its ^1^HNMR spectrum exhibited a singlet signal associated with the hydroxyl group at *δ* = 11.61 ppm, beside the olefinic proton of the indole ring at *δ* = 10.75 ppm. Further, ^13^C NMR highlighted the formation of compound 3 with significant carbons resonating at *δ* 24.51, 49.91, 50.34, 50.47, 79.58 ppm corresponding to five methylene carbons.

**Fig. 3:**
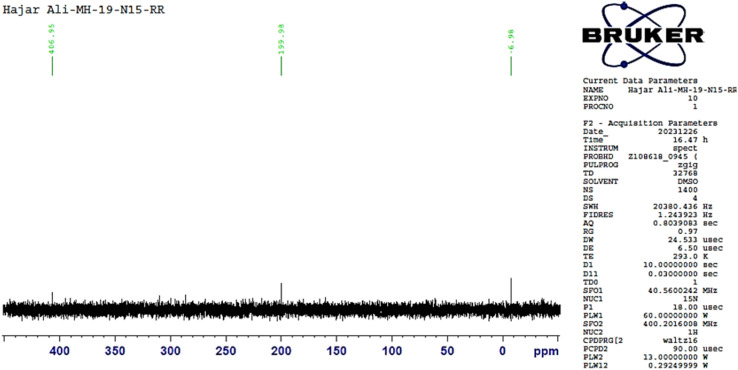
^15^N-NMR spectrum of compound 6.

It was noteworthy that bis-triazine 6 was synthesized, instead of the anticipated diphenylboraneyl ethoxy triazine 7. Initially, the reaction was carried out with the detachment of 2-aminoethyl diphenylborinate (5), followed by dimerization to afford 2,2′-oxybis(ethan-1-amine) (6A).^38–40^ Ultimately, Mannich product 6 was obtained by aminomethylation of oxybis(ethanamine) 6A with 1 and CH_2_O. The resulting product 6 was separated chromatography and characterized by ^1^HNMR spectrum with a distinctive triplet signal at *δ* 2.54 due to aza methylene protons (–NCH_2_C–), in addition to four multiplet signals at *δ* 3.53–3.56, 4.61–4.65, 7.03–7.07, and 7.35–7.40 corresponding to oxy ethylene (–OCH_2_CH_2_N–), methylene-H's of triazine ring, the other singles were related to aromatic protons, respectively. Meanwhile, ^13^CNMR spectrum of 6 showed 11 signals including; the methyl of acetyl group reverberated at *δ* 23.84 (2C), the methylene carbons bonded to nitrogen and oxygen atoms appeared at *δ* 55.96 (2C), 59.83 (2C), respectively and the methylene carbon of triazine ring showed at *δ* 46.44 (2C) and 65.11 (2C). Moreover, the quaternary carbons of the triazine ring appeared at *δ* 145.26 (2C), and the carbons of the carbonyl group resonated at 196.01 (2C). In contrast, all these quaternary carbons disappeared in DEPT (135) as shown in the ESI file.† On top of that, six nitrogen atoms were clearly evidenced by ^15^N NMR spectroscopy and revealed signals at −6.98, 199.98, and 406.95 ppm (Fig. 3).

**Scheme 2:**
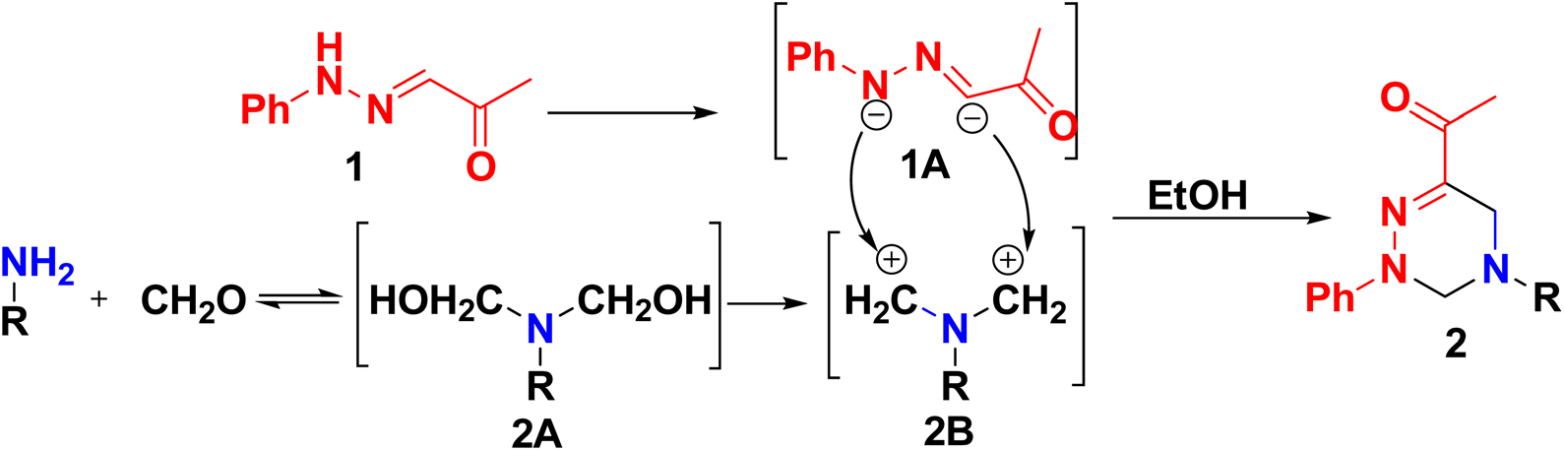
A postulated mechanism for the formation of triazine derivatives.

In general, the preparation of triazine derivatives may be rationalized based on a depicted mechanism (Scheme 2) involved *via* bis-Mannich reaction ongoing with the condensation of amine with formalin to produce an intermediate methylol derivative 2A, followed by removal of the two hydroxyl groups to afford carbonium ion 2B, RN(CH_2_^+^)_2_, in addition, elimination of two acidic protons from 1 yielded the carbanion intermediate 1A. Finally, a carbanion 1A reacted with carbonium ions 2B to form triazine derivatives 2.^41,42^

**Scheme 3:**
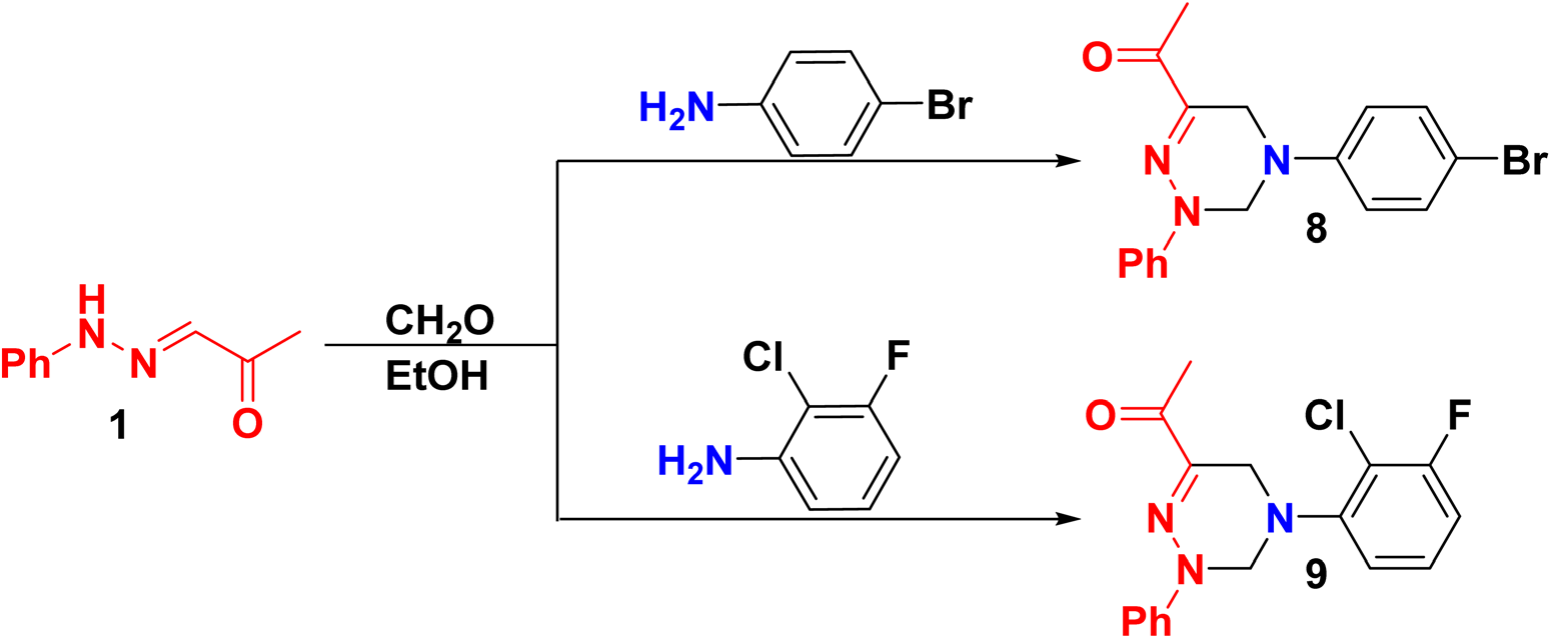
Treatment of 1 with substituted aniline.

Similarly, refluxing of 1 with 4-bromoaniline or 2-chloro-3-fluoroaniline and CH_2_O in EtOH furnished bromophenyl-2-phenyl-tetrahydro-triazinethanone 8 and (chloro-fluorophenyl-2-phenyl-tetrahydro-triazinyl)ethanone 9, respectively (Scheme 3). The structures of compounds 8 and 9 were supported by analytical and spectral data. As the mass spectra of constitutions 8 and 9 showed molecular ion peaks (*m*/*z*) at 358.41 and 331.54, respectively. Also, ^13^C NMR of skeleton 8 revealed 13 carbon signals corresponding to the elucidated constitution. However, the presence of a fluorine atom in compound 9 will cause signal splitting in ^13^C NMR due to a phenomenon known as the ^19^F–^13^C coupling so ^13^C analysis cannot be performed.^43,44^ To provide additional confirmation of the formation of compound 9, ^19^F NMR was conducted, revealing a singlet signal at −*δ* 126.87 ppm related to one fluorine atom.

**Scheme 4:**
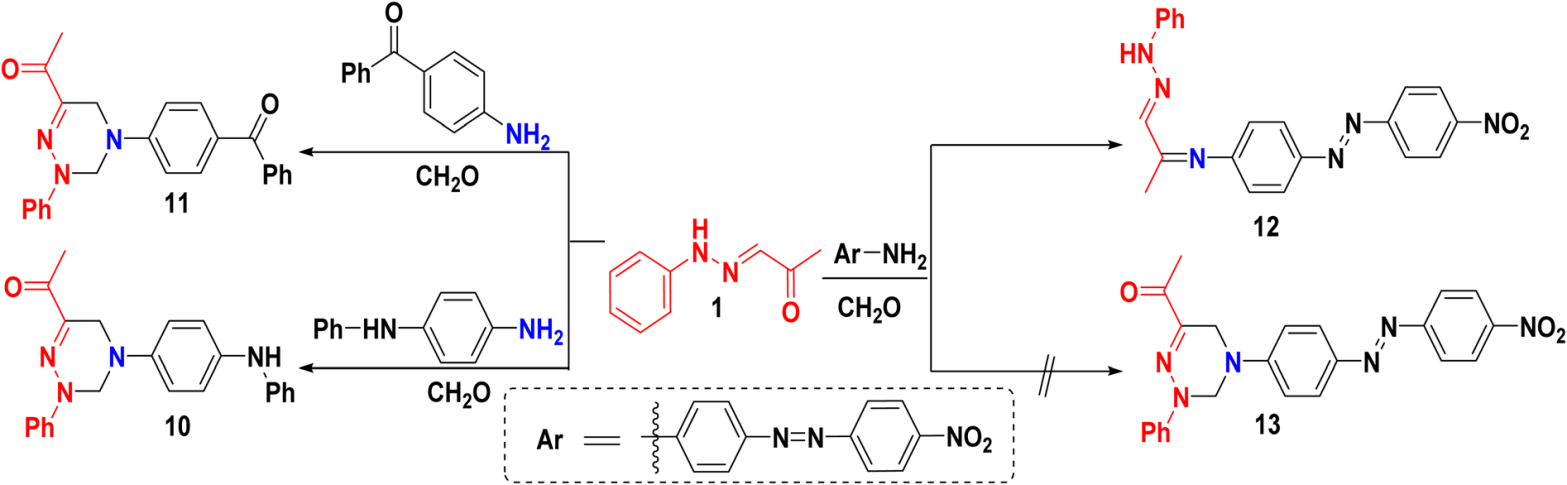
Synthesis of polyfunctionalized triazines with different spacers.

Encouraged by the aforementioned results, we expanded our research to include aromatic amines with different spacers, such as imino, carbonyl, and azo groups. Mannich reaction of 1 with *N*-phenyl-*p*-phenylenediamine or 4-aminobenzophenone and CH_2_O afforded ((4-(phenylamino)phenyl)tetrahydrotriazin)ethanone 10, and (4-benzoylphenyl)-2-(phenyl-tetrahydro-triazin-6-yl)ethanone 11, respectively, as illustrated in (Scheme 4). The IR spectrum of compound 10 displayed a characteristic band at 3353 cm^−1^, corresponding to the NH group. Additional supporting evidence for the skeleton 10 was provided by ^1^HNMR, which displayed four singlet signals at *δ* = 7.92, 5.20, 4.18, and 2.36 ppm related to the NH group, two methylene groups, and the CH_3_CO– group, respectively. In addition, its mass spectrum showed *m*/*z* at 370.41, which was in accordance with its molecular weight (see experimental part). The IR spectrum of compound 11 showed a prominent absorption band at 1723 and 1669 for (2C=O). Furthermore, its ^1^H NMR exhibited three singlet signals for the methyl of acetyl group and two methylene-H's of the triazine moiety at *δ* 2.39, 4.39, and 5.41 ppm. Meanwhile, the two carbonyl groups emerged in the ^13^C analysis at *δ* 194.55 and 195.64 ppm, yet concurrently, they were substantiated to have dissipated in the DEPT 135 spectrum.

In this line, we aimed to synthesize compound 13*via* Mannich reaction, while ((4-nitrophenyl)diazenyl)phenyl-1-(phenylhydrazineylidene)propanimine 12 was prepared through condensation of an amino group with the ketonic group of starting material 1. The IR spectrum of compound 12 revealed the presence of a new NH (PhNHN–) absorption band at 3272 cm^−1^. Whereby, ^1^HNMR of compound 12 confirmed the structure as revealing a singlet resonating signal at *δ* 13.33 ppm assigned to the NH group of 1. However, the protons of the two methylene groups were absent. Furthermore, its mass spectrum proved to possess *m*/*z* at 386.48.

**Scheme 5:**
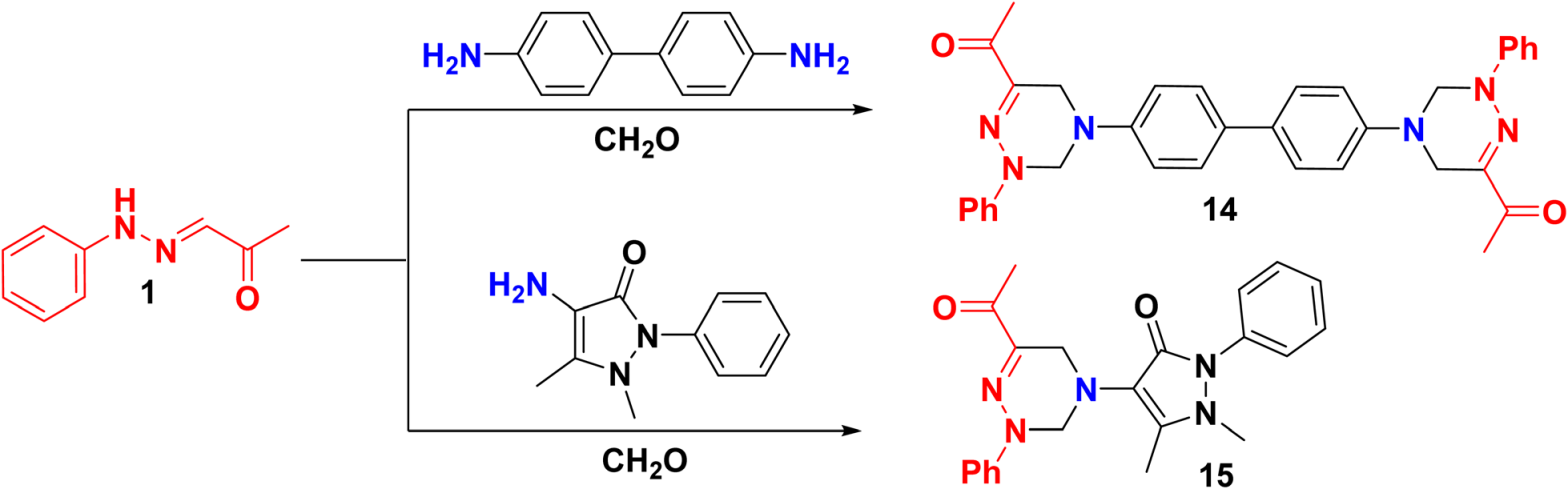
Double Mannich reactions for the construction of triazines.

The scope of the preparation of bis triazine derivatives was explored by refluxing 1 with benzidine and CH_2_O *via* bis double Mannich, yielding biphenyl-4,4′-bis(2-phenyl-tetrahydro-1,2,4-triazine-4,6-diyl)bis-ethan-1-one 14 as the anticipated product (Scheme 5). Once more, the constitution of compound 14 was based on mass spectrum, which showed a molecular ion peak at 556.87 (M^+^, 32.96%). On the other hand, ^13^C NMR displayed 14 carbon signals corresponding to C_34_H_32_N_6_O_2_.

**Fig. 4:**
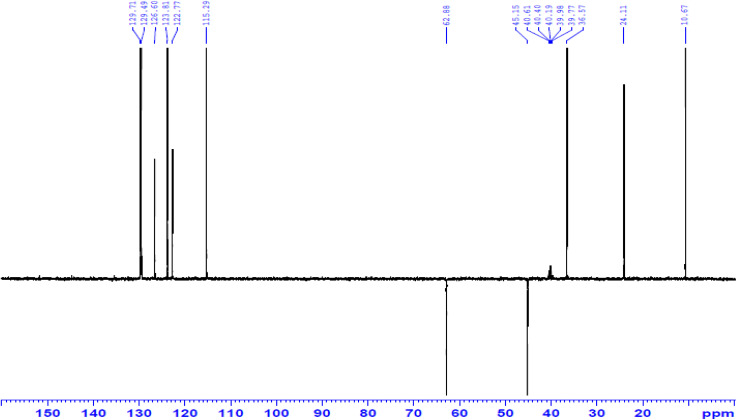
DEPT (135) spectrum of compound 15.

Likewise, the Mannich reaction of 1 with amino antipyrine and CH_2_O in refluxing EtOH afforded constitution 15 (Scheme 5). Notably, the DEPT 135 spectrum confirmed distinct signals according to the elucidated structure, as the spectrum exhibited the disappearance of signals associated with the quaternary carbon at *δ* 117.65, 135.40, 140.61, 144.76, and 151.81 ppm, as well as two carbonyl carbons at *δ* 162.56 and 195.64 ppm. Additionally, two methylene signals with negative phases were observed at 45.15 and 62.88 ppm, while characteristic positive signals were detected at *δ* 10.67, 24.10, and 36.57 ppm, corresponding to three methyl groups (Fig. 4).

**Fig. 5:**
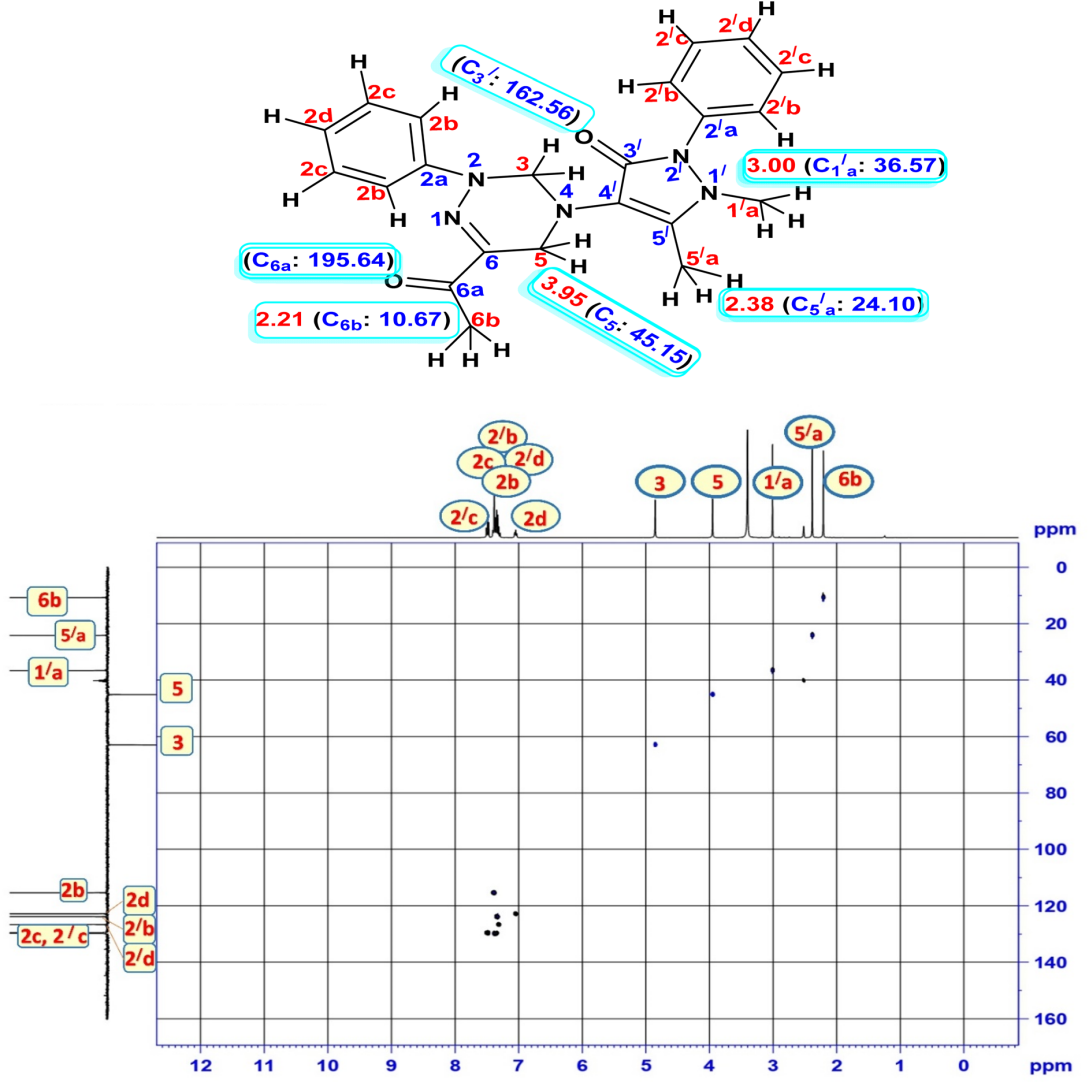
Two-dimension HSQC spectrum of compound 15.

The ^1^H, ^13^C NMR, and 2D NMR experiments, including the HSQC pulse sequence, provided valuable insights into the structural composition of compound 15. The acquired data revealed correlation plots, including the alpha methyl protons adjacent to the ketone group at *δ* 2.21(C_6b_: 10.67) as a singlet signal. Additionally, two methylene groups were observed at *δ* 3.95 (C_5_: 45.15), *δ* 4.85 (C_3_: 62.88), two methyl groups at *δ* 2.38 (C_5′a_: 24.10) and 3.00 (C_1′a_: 36.57) of the pyrazole ring Fig. 5.

**Scheme 6:**
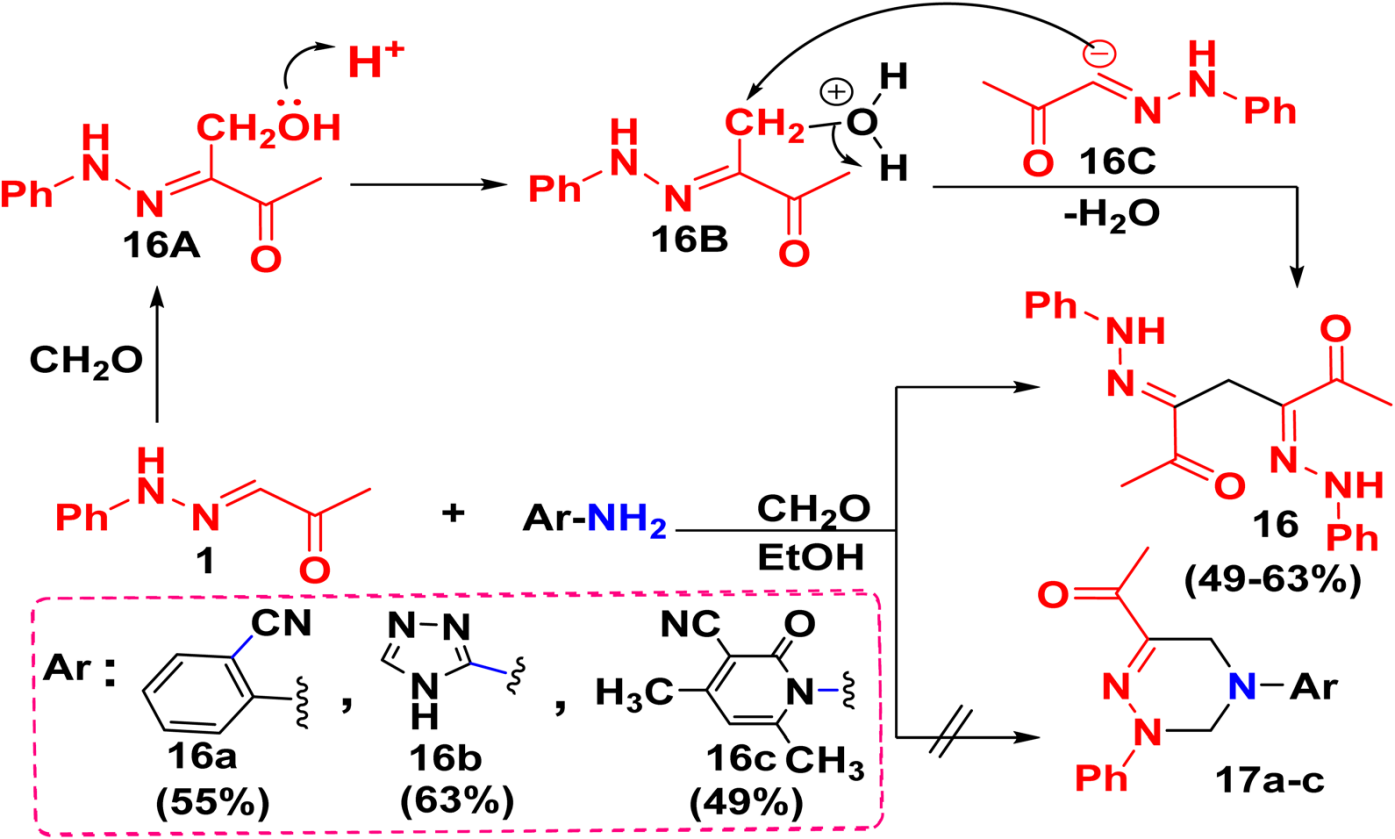
A deamination reaction mechanism of 1 for the synthesis of bis ketones.

Multi-component reaction of 1 with 2-aminobenzonitrile or aminotriazole, or aminopyridine derivative with CH_2_O was expected to follow the same synthetic route to synthesize acetyl triazine derivatives 17a–c, but did not result in this formation, whereas, a deamination reaction, which was a major problem of Mannich reaction was occurred that might be due to the long time of the reaction or unwanted side reaction or a drastic reaction conditions which led to a methylene bisketone synthesis 16 (Scheme 6).^45^ Compound 16 was evidenced by ^1^HNMR which revealed the presence of characteristic signal related to two symmetrical NH groups at *δ* = 10.97 ppm, in addition to one methylene group at *δ* = 3.75 ppm. Consequently, ^13^C NMR displayed 8 signals, corresponding to 19 carbons, with a characteristic methylene carbon at *δ* = 19.82 ppm.

Al-Mousawi et al.^46^ proposed a mechanistic pathway for the synthesis of bis(2-phenylhydrazineylidene)heptane-2,6-dione 16, starting with a methyolation reaction of one mole of 1 with one mole of CH_2_O in refluxing ethanol to afford a monomethylol intermediate 16A. On the other hand, the elimination of an acidic proton from another mole of 1 produced an anion intermediate 16C. Intermediate 16A was protonated to convert the bad leaving group (–OH) to the good leaving group (H_2_O) of intermediate 16B. Finally, bis-ketone product 16 was produced by condensing the anion intermediate 16C with intermediate 16B (Scheme 6).

Indeed, ultrasound is a highly green activation technique to meet the concept of sustainable chemistry in total organic synthesis,^47^ as it accelerates the reaction rate and allows significant results to be obtained in terms of atom economy through one-pot fashion, without purification of intermediates leading to lower time, costs and energy consumption over the conventional thermal methods.^48–51^ Therefore, we explored the obtained yields in the application of the Mannich reaction *via* both an ultrasound-assisted method and contrasting its efficacy with conventional synthetic techniques for the following compounds (Table 1).

**Table 1:** Time and yield of conventional method *versus* ultrasound-promoted synthesis of triazines

Compd	US method		Conv. method		Comp.	US method		Conv. method	
	Time (min)	Yield (%)	Time (min)	Yield (%)		Time (min)	Yield (%)	Time (min)	Yield (%)
2	45	66	45	47	10	45	66	45	42
	60	76	60	50		60	75	60	50
	75	84	75	52		75	79	75	56
	90	90	90	56		90	86	90	59
	105	93	105	64		105	95	105	67
	120	96	120	67		120	98	120	78
3	45	71	45	22	11	45	69	45	37
	60	78	60	43		60	71	60	44
	75	81	75	49		75	77	75	49
	90	84	90	55		90	79	90	51
	105	88	105	67		105	89	105	67
	120	90	120	78		120	94	120	72
6	45	40	45	20	12	45	52	45	35
	60	45	60	23		60	67	60	41
	75	51	75	28		75	71	75	55
	90	54	90	33		90	74	90	58
	105	58	105	36		105	89	105	62
	120	62	120	42		120	97	120	70
8	45	80	45	35	14	45	45	45	31
	60	85	60	50		60	54	60	37
	75	90	75	55		75	60	75	44
	90	92	90	60		90	64	90	58
	105	95	105	75		105	79	105	61
	120	97	120	80		120	85	120	68
9	45	31	45	21	15	45	71	45	22
	60	34	60	28		60	78	60	43
	75	38	75	31		75	85	75	49
	90	45	90	35		90	89	90	55
	105	49	105	41		105	91	105	67
	120	54	120	48		120	95	120	78

Additionally, chirality is one of the fundamental characteristics of molecular asymmetry and plays an essential role in the manufacture of pharmaceuticals in governing molecular interactions and therapeutic efficacy.^52^ The majority of commercially available drugs are chiral,^53^ approximately 90% of chiral pharmaceuticals approved by the FDA exist as racemates-equimolar mixtures of two enantiomers. Although racemates share identical chemical composition and bonding patterns, their biological behavior diverges significantly in chiral environments. This dichotomy manifests in distinct pharmacological, toxicological, metabolic, and pharmacokinetic profiles.^54–57^ Whereby, chirality influences the metabolism of drugs by activating one enantiomer more than the other.^58^

Whereby, the synthesized constitutions possess nitrogenous stereocenters, optical rotations for the synthesized scaffold were assessed at a concentration (weight percent) = 0.0033%. Each solution was held in the glass cell with a length of 17.5 cm. The thin film was determined at a temperature of 21 °C and wavelength 589 nm on WXG-4. UV polarimeter, the specific rotation is represented by [*α*]_*λ*_^*T*^ = *θ*/*CL*, with being the rotational angle, *C* the concentration, and *L* the optical path length of the chiral liquid^59–61^ (Table 2).

**Table 2:** Specific rotation of triazine scaffolds containing chiral nitrogenous atoms

Compd	Solvent	*θ*	*C*	*L*	[*α*]_589_^21^
1	DMF	5	0.0033	17.5	86.58
2	DMF	9	0.0033	17.5	155.84
3	DMF	7	0.0033	17.5	121.21
6	DMF	12	0.0033	17.5	207.79
8	DMF	5	0.0033	17.5	86.58
9	DMF	5	0.0033	17.5	86.58
10	DMF	12	0.0033	17.5	207.79
11	DMF	10	0.0033	17.5	173.16
12	DMF	16	0.0033	17.5	277.05
14	DMF	4	0.0033	17.5	69.26
15	DMF	6	0.0033	17.5	103.89

### 2.1 Antibacterial screening of the synthesized compounds

The bioactivity assessment revealed that some triazine derivatives exhibited considerable effectiveness against the tested bacteria, as demonstrated in Table 3 and Fig. S53.†

**Table 3:** Presented the inhibition zone diameter (IZD, mm) of bacteria strains treated with triazine compounds compared to the control, azithromycin, after incubation for 72 h at 28 °C

Compound	IZD			
Bacteria strain	*B. subtilis*	*S. epidermidis*	*Entero. Cloacae*	*E. coli*
1	13	14	—	—
2	—	—	—	12
3	15	16	—	12
6	—	—	—	—
8	—	—	—	18
9	39	50	41	40
10	22	20	14	17
11	12	—	—	11
12	—	11	—	20
14	19	32	20	15
15	14	11	11	18
Positive control (azithromycin, 2 mg ml^−1^)	24	22	12	20
Negative control (DMSO)	—	—	—	—

### 2.2 Molecular docking studies

Additional enhancements can be implemented in *in vitro* research techniques to facilitate the rapid screening of enzyme inhibitors by applying molecular modeling. Consequently, integrating bioinformatics simulations with *in vitro* analyses is beneficial for assessing the biological activities of triazine compounds. Molecular docking provides valuable insights into the binding interactions between protein targets derived from bacterial strains and all the synthesized compounds,^62,63^ thereby enhancing our understanding of the underlying biological mechanisms. In this approach, we shed light on the notable inhibitory effects of triazines against the four bacterial strains through an *in silico* computational molecular docking study. The findings from this approach, illustrated in Tables 4–7, employed the compounds' screening of binding affinities with four bacterial protein receptors.

**Table 4:** Represented the molecular docking results involving bond length, amino acid interactions, and binding energy

Compd	Binding affinity (kcal mol^−1^)	H-bond interaction	H-bond length in Å	Hydrophobic as well as other interactions
1	−5.117	THR15	2.18	ILE173, PRO192, GLU61, ASP190
		ASP187	2.84	
2	−6.376	THR15	1.97	ILE12, LEU243, ASP187, VAL191, PRO192, GLU61, THR15
3	−9.089	THR15	1.80	ILE12, ASP187, PRO192, GLU61, THR15, TYR250
6	−7.203	THR15	2.34	ALA9, GLU188, ASP187, ASP190, ASP14, THR15, ILE173, PRO192, GLU61
		ILE12	2.19	
8	−7.555	THR15	2.02	THR15, ILE12, ASP187, PRO192, GLU61
9	−7.905	LEU184	2.44	ARG251, LYS180, GLU179, SER186, GLU61, ASP187, HIS175
10	−8.588	THR15	1.93	GLU61, PRO192, THR15, ILE12, ASP187, ASP190, LEU243
11	−8.985	THR415	2.79	VAL225, PRO226, ALA227, HIS497
		HIS419	2.62	
		GLY417	2.91	
12	−6.966	TYR381	2.72	SER216, GLY382, LEU219, PRO226, PRO212, PRO209
14	−9.084	—	—	ASP14, GLU61, ASP187, HIS175, LEU184, LYS180, ARG251, LYS63
15	−8.027	HIS497	2.79	THR260, CYS322, PRO226, ALA227, ARG416
		HIS419	2.30	
		THR415	2.85	

**Table 5:** Data extracted from the docking interactions of triazines against the 8P20 protein

Compd	Binding affinity (kcal mol^−1^)	H-bond interaction	H-bond length in Å	Hydrophobic as well as other interactions
1	−5.423	LYS74	2.70	LYS150, VAL152, TYR147, LYS148, ASP171, LYS74, LYS44
		ALA45	2.07	
2	−6.157	LYS44	2.79	LYS148, VAL152, LYS150, ASP171, ILE71, LEU51, LYS52, VAL55, LYS48
3	−8.203	ARG179	2.44	ALA45, TYR145, PHE43, LYS153, LEU163, GLU166, ILE181
6	−7.520	PHE43	3.22	TYR145, LYS153, ALA45, GLU155, GLU166, PHE43, ILE188
			1.96	
8	−7.259	TYR145	2.84	TYR145, PHE43, VAL152, TYR147, ALA45
9	−7.372	GLY16	2.35	GLU266, VAL250, GLU403, LEU407, GLY17, MET18
		THR19	2.57	
10	−8.020	—	—	LEU163, PHE43, TYR145, GLU155, ALA45, GLU129, ILE181, TYR147
11	−8.382	LYS233, ASN203	2.31, 2.57	LYS264, TYR268, PRO232, VAL204, ILE188, LYS 233
			2.14	
12	−8.195	LYS233, ASN203	2.41	VAL262, ALA261, LYS264, ASP267, TYR268, PRO232, VAL204
			2.41	
14	−9.797	TYR268	2.40	VAL204, PRO232, ILE188, LEU163, ILE181, LYS264, TYR268
		ARG179	2.31	
15	−9.561	SER408, LEU409	2.31	TYR382, ILE324, LEU407, SER325, GLU403, GLY406, GLY17, GLY16, THR20
			2.47	
		ARG326	2.46	

**Table 6:** The docking interactions and the binding energy of inhibitors with the 1KQB protein

Compd	Binding affinity (kcal mol^−1^)	H-bond interaction	H-bond length in Å	Hydrophobic as well as other interactions
1	−5.082	PHE48	2.92	LYS31, LEU34, PHE48, HIS47
		HIS47	2.05	
2	−5.815	—	—	LEU34, PHE48, HIS47, PHE183, TRP94, ALA93, TRP46
3	−7.519	—	—	LYS31, LEU34, PHE48, HIS47, VAL98, ARG97, TRP94
6	−6.469	GLN44	3.24	ASP105, LYS31, PHE48, LEU34
		ARG97	3.07	
		TRP46	2.07	
8	−6.714	—	—	TYR144, LYS141, LEU186, ALA140, GLN137, PHE167, ALA169
9	−6.880	LYS31	2.16	ILE49, PHE48, VAL50, LYS31
		VAL50	3.36	
10	−7.291	PHE167	2.25	ASP136, THR184, ALA140, GLN137, LEU186, ALA169, ALA170, ASP168
11	−7.079	—	—	TYR144, LEU186, GLN137, ALA140
12	−7.031	SER40	2.02	PRO163, GLU165, VAL187, LEU145, LYS141, GLN142, TYR144
		TRP138	2.18	
		TYR144	3.38	
14	−8.497	SER40, TRP138	2.05	LYS141, LEU145, TYR144, VAL147, PRO163, VAL187, TRP138
			2.31	
15	−8.107	GLN35	2.99	LEU34, LYS31, PHE48, ARG97, TRP94, TRP46
		ARG97	2.24, 2.56	

**Table 7:** Molecular docking interaction of target compounds against DNA gyrase protein 1KZN

Compd	Binding affinity (kcal mol^−1^)	H-bond interaction	H-bond length in Å	Hydrophobic as well as other interactions
1	−5.755	ASN46	2.10, 2.73, 2.53, 2.61	ILE78, ASP49, GLU50
2	−6.284	—	—	ALA86, ILE90, PRO79, ASN46, ALA47
3	−8.161	GLY77	2.65	ALA96, ILE90, VAL167, THR165, ALA47
		GLU42	3.05	
		ASN46	2.67	
6	−7.972	GLY117	1.94	PRO79, ILE78, ALA47, ASN46, ASP49, ASP45
		HIS116	2.40	
8	−7.345	ARG136	2.52, 2.74	ALA53, ASP49, ARG76, ILE78, VAL167, VAL120, ASN46
9	−7.544	ARG136	2.53	GLY77, GLU50, ALA47, ASN46, ILE78, ARG76, PRO79
		ARG76	2.63	
		ASN46	2.55	
10	−7.878	ARG76	2.19	PRO79, ILE90, ASN46, GLY77, VAL89, ILE78, ALA86
		GLU50	3.37	
11	−8.141	ARG76	2.89	ARG76, ALA53, LEU52, ILE78, ALA47, THR165, ASN46
12	−6.959	ASN46	2.83	ALA47, VAL43, THR165, VAL167, ASP73, ILE78, ILE90, PRO79, ALA86
14	−8.184	ARG76	2.38	VAL84, VAL89, ALA86, PRO79, ILE90, ASN46, ILE78
15	−8.236	ASN46	2.40	ILE78, ALA96, GLY119, GLU42, ILE90
		HIS95	2.89	

#### 2.2.1 Docking and molecular protein interaction in *Bacillus subtilis* (PDB: ID 1OF0)^64^ with triazine compounds

**Fig. 6:**
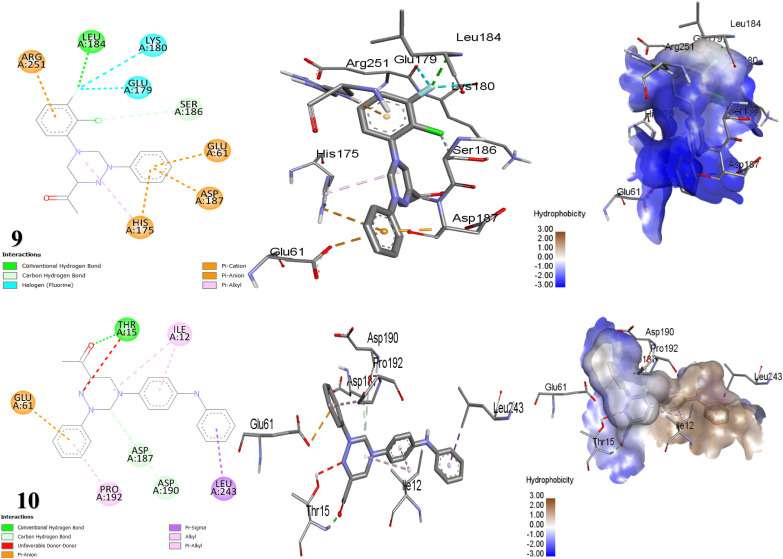
Depictions of the 2D, 3D structures, and hydrophobic view of scaffolds 9 and 10 against 1OF0 protein.

Docking studies forecasted that compounds 3, 14, and 10 have the strongest binding affinity. Nonetheless, *in vitro* studies indicated that compound 9 stands out among all the synthesized compounds as it is a potent antibacterial against all tested bacteria. Moreover, compound 10 revealed astonishing inhibitory potential against *B. subtilis*, manifesting an inhibition zone diameter of 22 mm. The fluorine atom on the substituted phenyl ring of compound 9 with 1OF0 receptor forms a H-bond with key amino acid LEU184 (bond length: 2.44 Å) and hydrophobic interactions with SER186 (C–H bond), LYS180, GLU179 (two fluorine bonds), ARG251, HIS175 (π-cation), GLU61, ASP187 (π-anion) and HIS175 (π-alkyl) (Table 4, Fig. 6). Whereby, compound 10 forms one conventional H-bonds with 1OF0 protein with bond length 1.93 Å with amino acid THR15. Furthermore, eight hydrophobic bonds were noted with THR15, ILE12, LEU243, ASP190, ASP187, PRO192, and GLU61. One potential explanation for the discrepancy observed between theoretical predictions and experimental results is the limitations of docking calculations. These calculations employ mathematical models and algorithms to forecast how drugs interact with biological targets. However, they overlook critical factors such as how drugs are delivered, variations in experimental conditions, and the properties of the biological targets themselves.^65^ Meanwhile, the antibacterial assessment revealed that neither compounds 2 nor 6, 8, and 12 inhibited the growth of *Bacillus subtilis*. CotA laccase, a multicopper oxidase, in *B. subtilis* (PDB ID: 1OF0), engage in the formation and stabilization of the spore coat. This enzyme parades oxidoreductase activity and has been structurally characterized in the presence of ABTS, a standard non-catalytic substrate used to assess laccase function. CotA contributes to protect spores from environmental challenges such as ultraviolet radiation, oxidative stress, and enzymatic degradation during germination. Remarkably, triazine-based compounds have demonstrated inhibitory effects on *B. subtilis* growth by disrupting CotA laccase activity.^66^

#### 2.2.2 Docking and molecular interaction of HA-MRSE [TarM(Se)] glycosylates RboP-WTA with glucose protein in *Staphylococcus epidermidis* (PDB: ID 8P20)^67^ with target compounds

**Fig. 7:**
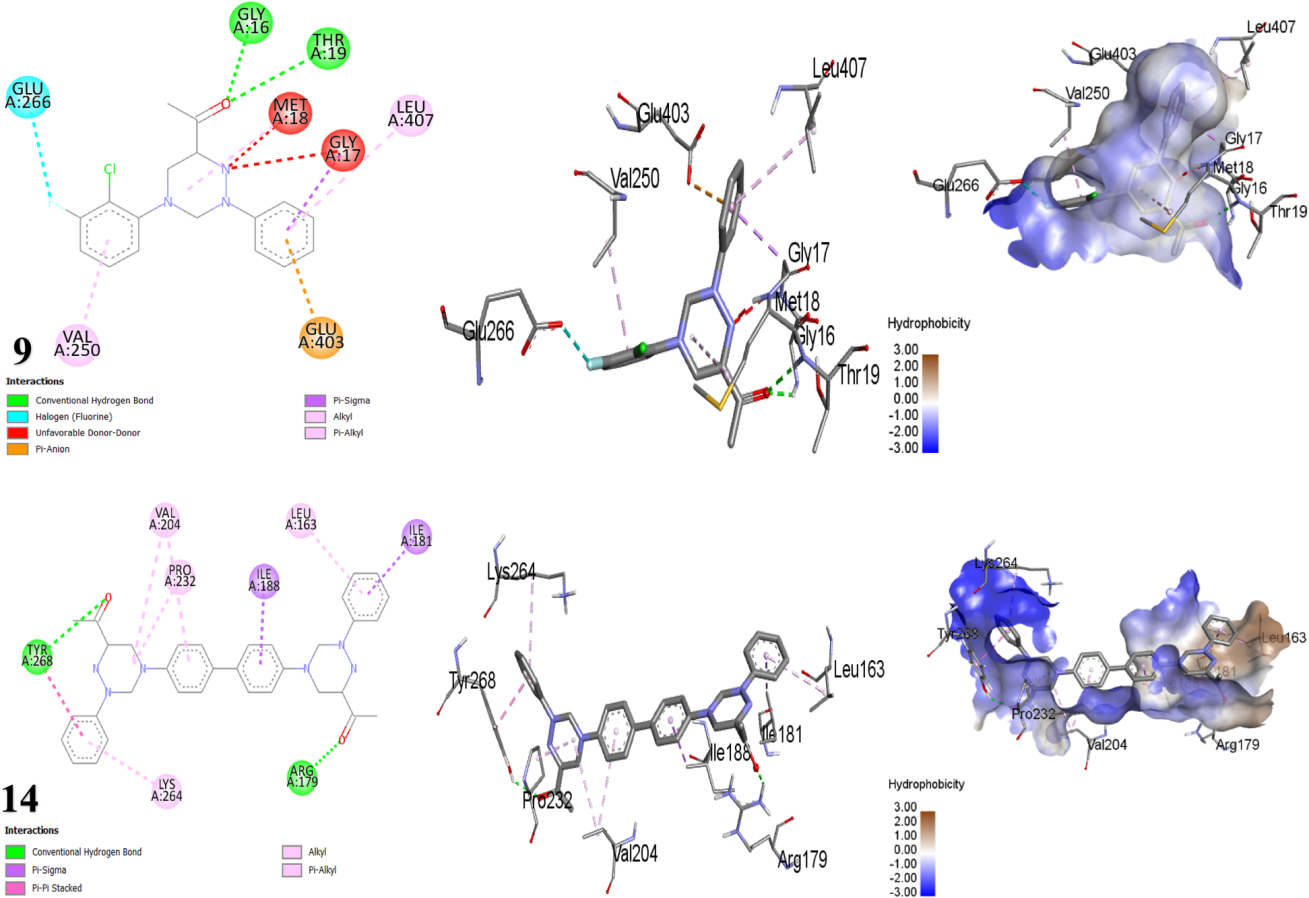
Binding mode and visual interaction of synthesized compounds 9 and 14 with the binding active site of 8P20 protein.

The tested compounds presented variable activities in inhibiting *S. epidermidis* bacteria. Notably, compounds 9 and 14 demonstrated tremendous antibacterial effects, while compound 10 established good inhibition. The binding interaction of these compounds with *S. epidermidis* may induce structural changes in the enzyme, potentially leading to its inhibition.^68^ Conversely, it was found that compounds 2, 6, 8, and 11 exhibited no effectiveness against *S. epidermidis*, suggesting that these compounds might possess additional antibacterial mechanisms beyond merely inhibiting bacteria.^69^ The docking results revealed a favorable binding affinity, with the lowest binding energy recorded at −9.797, −9.561, and −8.382 kcal mol^−1^ for compounds 14, 15, and 11, respectively. The docking outcomes are rather aligned with the experimental. A fluorine atom can establish a halogen bond with specific amino acid residues in the target protein, specifically (GLU266). The carbonyl oxygen atom on the acetyl group of hybrid 9 created two H-bonds with the key amino acids GLY16 and THR19. The binding model of compound 9 was stabilized by hydrophobic interactions between the atoms of scaffold 9 and the residues MET18, GLY17, LEU407, GLU403, VAL250, and GLU266. On the other hand, compound 14 formed two H-bonds between the oxygen atoms on its acetyl groups with the active-site-facing amino acids TYR268 and ARG179. In addition, the four aromatic rings of compound 14 engaged in hydrophobic interactions with various residues, including LEU163, VAL204, LYS264 (π-alkyl), PRO232, VAL204 (alkyl interaction), ILE181, ILE188 (π-sigma), and TYR268 (π–π stacked) (Table 5, Fig. 7). The 8P20 protein, also known as Small Basic Protein (Sbp), is an 18-kDa component of *S. epidermidis*, primarily localizes at the boundary between the biofilm and the underlying surface. Sbp plays a key structural role in supporting biofilm matrix and persistence, improving stable bacterial attachment, and facilitating the development of complex, multilayered biofilm communities. Additionally, it engages in biofilm construction mechanisms dependent on both polysaccharide intercellular adhesin (PIA) and the accumulation-associated protein (Aap), displaying co-localization with Aap's Domain-B, which suggests its engrossment in promoting Aap-driven biofilm accumulation. Moreover, Sbp contributes to bacterial cell clustering and reinforces the biofilm's overall integrity by forming amyloid fibrils. Notably, triazine scaffolds have been shown to inhibit the function of the 8P20 protein, potentially disrupting biofilm integrity and surface colonization.^70^

#### 2.2.3 Docking and molecular interaction of the nitro reductase enzyme in *Enterobacter cloacae* with tested compounds (PDB: ID 1KQB)^71,72^

**Fig. 8:**
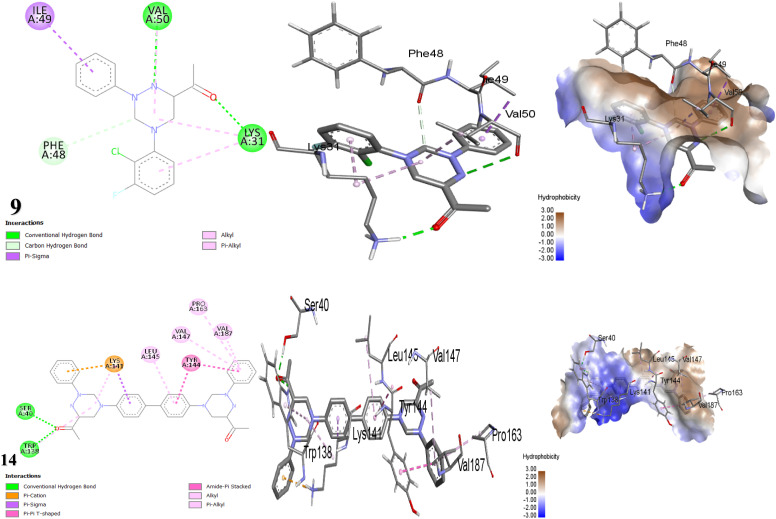
Molecular docking images of the inhibitors with the 1KQB protein.

Most of the tested compounds showed no activity toward *Enterobacter cloacae* except compounds 9, 10, 14 and 15. This is attributed to that the inactive compounds worked outside the cell membrane instead of inside it, leading them to bind to surface groups of the bacterial cells.^68^ As mentioned, compound 9 is an excellent paradigm against *Enterobacter cloacae* that showed an IZD of 41. It was revealed that the second most efficient compound in terms of antibacterial effect was compound 14. Compound 9 bonded with nitro reductase enzyme by two H-bonds with LYS31 and VAL50, and hydrophobic interactions with PHE48 (C–H Bond), ILE49 (π-sigma), VAL50, and LYS31 (alkyl, π-alkyl bond). Additionally, the conjugated system generated by the benzidine moiety as well as the substituted phenyl in compound 14 formed hydrophobic bonds with LYS141 (π-cation, π-sigma & alkyl), TYR144 (π–π, amide-π), TRP138, VAL147, PRO163, VAL187, and LEU145 (π-alkyl) that encourage electron delocalization, inducing its antibacterial activity. The individual significant H-bond interaction in compound 14 was formed between the oxygen of the carbonyl group and SER40, TRP138 over an intermolecular distance = 2.05 and 2.31 Å (Table 6, Fig. 8). The protein 1KQB from *Enterobacter cloacae* is a nitroreductase enzyme that belongs to a family of evolutionarily conserved proteins involved in the reduction of nitrogen-containing substrates. The oxidized form of nitroreductase has been elucidated through crystallographic studies in complex with benzoate, offering valuable insights into its catalytic mechanism and identifying potential sites for inhibitor binding. Triazine hybrids effectively inhibit the activity of nitroreductase, leading to the suppression of bacterial growth by targeting 1KQB.^73^

#### 2.2.4 Docking and molecular interaction of triazine scaffolds with DNA gyrase in *Escherichia coli* (PDB: ID 1KZN)^74,75^

**Fig. 9:**
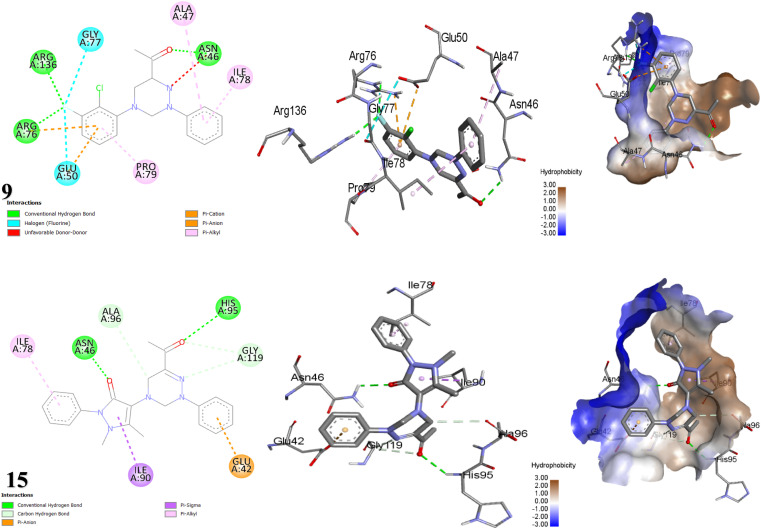
Interaction modes of compounds 9 and 15 with *E. coli* protein (PDB: 1KZN).

The forecast by docking studies anticipated that compounds 15 > 14 > 3 demonstrate very high inhibitory binding affinities (Table 7). Nevertheless, compound 9 manifested the highest inhibition zone equal to 40 mm in the antibacterial assay, which is attributed to the dynamic interactions stemming from the presence of two halogens, F and Cl in compound 9, which generate a dipole moment because of their electronegativity, thereby enhancing the compound's binding affinity.^76,77^ For the bacterium *E. Coli*, the docking results illustrated in Fig. 9 showed that the interaction mechanism between scaffold 9 and the 1KZN protein relies mainly on three H-bonds with amino acids ARG136, ARG76, and ASN46 at distances of 2.53 Å, 2.63 Å and 2.55 Å, respectively, as well as, two F-bonds with GLY77 and GLU50 along with hydrophobic interactions. Whereas, compound 15 exhibited a binding energy of −8.236 kcal mol^−1^ due to its participation in H-bond interactions with ASN46 and HIS95. Additionally, it engages in π-sigma interactions with ILE90, two C–H bonds with GLY119, ALA96, π-anion with GLU42, and π-alkyl interactions with ILE78 (Table 7). The 1KZN protein in *E. coli* signifies the 24 kDa N-terminal domain of the DNA gyrase subunit B (GyrB). DNA gyrase is a type II topoisomerase that demonstrates a crucial role in introducing negative supercoils into double-stranded DNA, a process essential for DNA transcription, replication, and the maintenance of DNA topology in bacteria. The GyrB subunit is essential for catalyzing ATP hydrolysis, which provides the energy needed for the supercoiling activity of the enzyme. Triazine derivatives have been demonstrated to inhibit the action of 1KZN protein, resulting in inhibiting the growth of bacteria.^78^

### 2.3 SAR studies

Notably, triazine derivatives bearing electron-withdrawing atoms such as fluoro and chloro, on the aromatic ring, as exemplified by compound 9, significantly exhibit the highest potency with a minimum inhibitory concentration (MIC) of 1.87 mg mL^−1^.^17*b*,79,80^ This enhancement is attributed to the presence of strongly electronegative halogenated atoms,^81^ which can increase the electrophilicity of the triazine core,^82^ potentially improving interactions with bacterial targets and facilitating better cell penetration.^83^ In contrast, incorporating a bulky, electron-donating dodecyl group, as in compound 2, resulted in a detrimental effect on antibacterial activity against most bacterial strains. On the other hand, introducing a hydrogen-bond acceptor, exemplified by an ethanol moiety in the tryptamine derivative 3, was compatible with the activity and increased it. Furthermore, linking the triazine ring with pyrazolone and phenyl moieties, as seen in compound 15, resulted in increased antibacterial activity.^83^ Whereby, the diazabiphenyl derivative 14 exhibited considerable antibacterial activity, which is thought to arise from the ability of its nitrogen atoms to form stabilizing hydrogen bonds with target proteins-an interaction often essential for the bactericidal action.^33^

## 3 Experimental

### 3.1 General methodology for preparation of 6-acetyl-1,2,4-triazine derivatives

One-pot three component reaction of (phenylhydrazineylidene)propan-2-one 1 (0.53 g, 3.3 mmol) with formalin (0.54 ml, 6.6 mmol, 37%) and various amines (3.3 mmol) involving dodecylamine, tryptamine, 2-((diphenylboryl)oxy)ethanamine (5), 4-bromoaniline, 2-chloro-3-fluoroaniline, *N*-phenyl-*p*-phenylenediamine, 4-aminobenzophenone, 4-((4-nitrophenyl)diazenyl)aniline, benzidine and 4-aminoantipyrine in ethanol was refluxed on water bath as well as by ultrasound method for 120 min as optimized time to afford of functionalized nitrogenous molecules 2, 3, 6, 8, 9, 10, 11, 12, 14 and 15, respectively. Whereby, the synthesis of bis(2-phenylhydrazineylidene)heptane-2,6-dione 16 was achieved through repeating the same Mannich reaction methodology of synthesizing the compounds as above-mentioned in the presence of 2-aminobenzonitrile or 4*H*-1,2,4-triazol-3-amine or 1-amino-4,6-dimethyl-2-oxo-1,2-dihydropyridine-3-carbonitrile as nitrogenous systems.

#### 3.1.1 1-(2-Phenylhydrazineylidene)propan-2-one (1)

Yield = 55%; red powder; Lit. m.p.^37^ = 148–150 °C, m.p. = 142–144 °C; *R*_f_ = 0.78 EtOAc/petroleum ether (1.5 : 4). ^1^HNMR (DMSO, d_6_); *δ* ppm 2.32 (s, CH_3_, 3H), 6.96 (t, *J* = 7.2 Hz, 1H_Ar_), 7.18–7.20 (m, 2H_Ar_), 7.25 (s, =CH, 1H), 7.31–7.35 (m, 2H_Ar_), 11.33 (s, NH, 1H). ^13^C NMR; *δ* ppm 24.60, 113.93 (2C), 122.11, 129.88 (2C), 135.14, 143.70, 196.84.

#### 3.1.2 1-(4-Dodecyl-2-phenyl-2,3,4,5-tetrahydro-1,2,4-triazin-6-yl)ethan-1-one (2)

Pale-yellow powder; m.p. = 56–58 °C; *R*_f_ = 0.67 EtOAc/petroleum ether (1.5 : 4). IR (*ν*^\^/cm^−1^): 2959, 2916, 2848 (sp^3^ C–H), 1726 (C=O). ^1^HNMR; *δ* ppm 0.86 (t, *J* = 6.6 Hz, 3H), 1.23 (m, 18H), 1.43–1.45 (m, 2H), 2.34 (s, CH_3_, 3H), 2.41 (t, *J* = 7.2 Hz, 2H), 3.49 (s, CH_2_ of –CCH_2_N–, 2H), 4.56 (s, CH_2_ of –NCH_2_N–, 2H), 7.03–7.07 (m, 1H_Ar_), 7.35–7.41 (m, 4H_Ar_). ^13^C NMR; *δ* ppm 14.44, 22.58, 23.83, 27.06, 27.30, 29.18, 29.26, 29.44, 29.48 (2C), 29.50, 31.77, 45.65, 53.33, 64.60, 115.19 (2C), 122.79, 129.72 (2C), 139.12, 145.24, 196.02. (EMIS) *m*/*z* (%): 371.14 (M^+^, 18.89%), 324.44 (57.86%), 203.02 (100%, base peak), 189.87 (65.15%), 185.00 (97.50%), 183.69 (56.24%), 175.22 (51.20%), 63.49 (48.98%). Anal. Calcd for C_23_H_37_N_3_O (371.57): C, 74.35, H 10.04, N 11.31%. Found C 74.32, H 10.06, N 11.27%.

#### 3.1.3 1-(4-(2-(1-(Hydroxymethyl)-1*H*-indol-3-yl)ethyl)-2-phenyl-2,3,4,5-tetrahydro-1,2,4-triazin-6-yl)ethan-1-one (3)

Yield = 63%; orange powder; m.p. = 182–184 °C; *R*_f_ = 0.17 EtOAc/petroleum ether (1 : 4). ^1^HNMR; *δ* ppm 1.25 (s, CH_2_, 2H), 2.44 (s, CH_3_, 3H), 2.72–2.79 (m, 4H), 3.66 (s, CH_2_, 2H), 3.88 (s, CH_2_, 2H), 6.93–7.04 (m, 3H), 7.19 (d, *J* = 7.6 Hz, 2H), 7.27–7.32 (m, 3H), 7.38 (d, *J* = 7.6 Hz, 1H), 10.75 (s, 1H), 11.61 (s, 1H, OH). ^13^C NMR; *δ* ppm 21.44, 24.51, 49.91, 50.34, 50.47, 79.58, 106.55, 111.43, 114.26 (2C), 117.90, 118.83, 120.95, 122.43, 127.00, 129.85 (2C), 132.37, 136.33, 139.03, 143.52, 196.40. (EMIS) *m*/*z* (%): 376.50 (M^+^, 20.70%), 367.40 (64.83%), 282.35 (28.81%), 275.35 (58.47%), 261.40 (31.84%), 233.11 (28.84%), 117.10 (100%, base peak), 89.23 (28.59%). Anal. Calcd for C_22_H_24_N_4_O_2_ (376.46): C 70.19, H 6.43, N 14.88%. Found C 70.23, H 6.46, N 14.85%.

#### 3.1.4 1,1′-((Oxybis(ethane-2,1-diyl))bis(2-phenyl-2,3,4,5-tetrahydro-1,2,4-triazine-4,6-diyl))bis(ethan-1-one) (6)

Yield = 68%; pale-yellow powder; m.p. = 78–80 °C; *R*_f_ = 0.27 EtOAc/petroleum ether (1 : 4). IR (*ν*^\^/cm^−1^): 3043 (sp^2^ C–H), 2954, 2920, 2879 (sp^3^ C–H), 1646 (C=O). ^1^HNMR; *δ* ppm 2.35 (s, 2CH_3_, 6H), 2.54 (t, *J* = 5.8 Hz, 4H), 3.53–3.56 (m, 8H), 4.61–4.65 (m, 4H), 7.03–7.07 (m, 2H_Ar_), 7.35–7.40 (m, 8H_Ar_). ^13^C NMR; *δ* 23.84 (2C), 46.44 (2C), 55.96 (2C), 59.83 (2C), 65.11 (2C), 115.25 (4C), 122.80 (2C), 129.72 (4C), 139.29 (2C), 145.26 (2C), 196.01 (2C). ^15^NNMR; *δ* ppm −6.98 (2N), 199.98 (2N), and 406.95 (2N). (EMIS) *m*/*z* (%): 476.06 (M^+^, 13.27%), 264.01 (65.26%), 173.95 (35.98%), 167.64 (51.63%), 160.01 (55.15%), 129.95 (100%, base peak), 104.53 (48.37%), 90.51 (85.63%). Anal. Calcd for C_26_H_32_N_6_O_3_ (476.58): C, 65.53; H 6.77, N 17.63%. Found C 65.50, H 6.81, N 17.60%.

#### 3.1.5 1-(4-(4-Bromophenyl)-2-phenyl-2,3,4,5-tetrahydro-1,2,4-triazin-6-yl)ethan-1-one (8)

Off-white powder; m.p. = 116–118 °C; *R*_f_ = 0.80 EtOAc/petroleum ether (1 : 4). IR (*ν*^\^/cm^−1^): 3065, 3026, 3005 (sp^2^ C–H), 2896 (sp^3^ C–H), 1653 (C=O). ^1^HNMR; *δ* ppm 2.35 (s, CH_3_, 3H), 4.24 (s, NCH_2_C, 2H), 5.28 (s, NCH_2_N, 2H), 7.00 (d, *J* = 8.8 Hz, 2H), 7.09 (t, *J* = 7.2 Hz, 1H), 7.39–7.42 (m, 4H_Ar_), 7.48 (d, *J* = 7.6 Hz, 2H_Ar_). ^13^C NMR; *δ* ppm 23.96, 44.78, 61.40, 112.69, 115.29 (2C), 119.35 (2C), 123.19, 129. 84 (2C), 132.37 (2C), 139.45, 144.72, 147.88, 195.67. (EMIS) *m*/*z* (%): 358.41 (M^+^, 46.49%), 270.57 (100%, base peak), 220.57 (89.53%), 211.01 (73.44%), 209.48 (65.42%), 204.77 (61.24%), 196.62 (66.04%), 43.61 (61.29%). Anal. Calcd for C_17_H_16_BrN_3_O (358.24): C 57.00, H 4.50, N 11.73%. Found C 57.04, H 4.49, N 11.71%.

#### 3.1.6 1-(4-(2-Chloro-3-fluorophenyl)-2-phenyl-2,3,4,5-tetrahydro-1,2,4-triazin-6-yl)ethan-1-one (9)

Pale-yellow powder; m.p. = 138–140 °C; *R*_f_ = 0.60 EtOAc/petroleum ether (1.5 : 4). IR (*ν*^\^/cm^−1^): 3061 (sp^2^ C–H), 2835 (sp^3^ C–H), 1654 (C=O). ^1^HNMR; *δ* ppm 2.36 (s, CH_3_, 3H), 4.23 (s, NCH_2_C, 2H), 5.26 (s, NCH_2_N, 2H), 7.01–7.11 (m, 2H_Ar_), 7.28–7.32 (m, 2H_Ar_), 7.41 (t, *J* = 7.8 Hz, 2H_Ar_), 7.50 (d, *J* = 8 Hz, 2H_Ar_). ^19^F-NMR; −*δ* 126.87 ppm. (EMIS) *m*/*z* (%): 331.54 (M^+^, 16.77%), 228.58 (89.42%), 215.18 (100%, base peak), 212.23 (68.87%), 169.11 (87.66%), 127.21 (77.75%), 81.26 (71.81%), 79.51 (81.06%). Anal. Calcd for C_17_H_15_ClFN_3_O (331.78): C 61.54, H 4.56, N 12.67%. Found C 61.52, H 4.59, N 12.63%.

#### 3.1.7 1-(2-Phenyl-4-(4-(phenylamino)phenyl)-2,3,4,5-tetrahydro-1,2,4-triazin-6-yl)ethan-1-one (10)

Brown powder; m.p. = 170–172 °C; *R*_f_ = 0.45 EtOAc/petroleum ether (1.5 : 4). IR (*ν*^\^/cm^−1^): 3353 (NH), 3023 (sp^2^ C–H), 2919 (sp^3^ C–H), 1659 (C=O). ^1^HNMR; *δ* ppm 2.36 (s, CH_3_, 3H), 4.18 (s, NCH_2_C, 2H), 5.20 (s, NCH_2_N, 2H), 6.73 (t, *J* = 7.4 Hz, 1H_Ar_), 6.93–7.02 (m, 6H_Ar_), 7.08 (t, *J* = 7.2 Hz, 1H_Ar_), 7.16 (t, *J* = 7.8 Hz, 2H_Ar_), 7.40 (t, *J* = 7.8 Hz, 2H_Ar_), 7.48 (d, *J* = 8 Hz, 2H_Ar_), 7.92 (s, NH, 1H, exchangeable with D_2_O). ^13^C NMR; *δ* ppm 23.90, 45.31, 62.59, 115.18 (2C), 115.82 (2C), 119.02 (2C), 119.11, 119.57 (2C), 123.04, 129.57 (2C), 129.84 (2C), 137.64, 139.60, 142.41, 144.79, 144.88, 195.76. (EMIS) *m*/*z* (%): 370.41 (M^+^, 30.99%), 367.48 (76.19%), 349.27 (93.95%), 347.23 (98.47%), 153.03 (69.31%), 91.41 (100%, base peak), 74.72 (66.44%), 68.29 (84.12%). Anal. Calcd for C_23_H_22_N_4_O (370.46): C 74.57, H 5.99, N 15.12%. Found C 74.53, H 6.01, N 15.15%.

#### 3.1.8 1-(4-(4-Benzoylphenyl)-2-phenyl-2,3,4,5-tetrahydro-1,2,4-triazin-6-yl)ethan-1-one (11)

Yellow powder; m.p. = 164–168 °C; *R*_f_ = 0.59 EtOAc/petroleum ether (1 : 4). IR (*ν*^\^/cm^−1^): 3061 (sp^2^ C–H), 2925, 2834 (sp^3^ C–H), 1723, 1669 (C=O). ^1^HNMR; *δ* ppm 2.39 (s, CH_3_, 3H), 4.39 (s, NCH_2_C, 2H), 5.41 (s, NCH_2_N, 2H), 7.11 (t, *J* = 7.2 Hz, 1H_Ar_), 7.20 (d, *J* = 8.8 Hz, 2H_Ar_), 7.43 (t, *J* = 7.8 Hz, 2H_Ar_), 7.52–7.56 (m, 4H_Ar_), 7.64–7.71 (m, 5H_Ar_). ^13^C NMR; *δ* ppm 24.13, 44.34, 59.96, 115.42 (2C), 115.50 (2C), 123.32, 128.55, 128.89 (2C), 129.64 (2C), 129.89, 132.41 (4C), 138.38, 139.75, 144.65, 151.92, 194.55, 195.64. (EMIS) *m*/*z* (%): 383.27 (M^+^, 33.51%), 349.57 (92.21%), 342.06 (76.87%), 333.08 (63.22%), 325.85 (86.58%), 320.23 (100%, base peak), 164.03 (90.16%), 149.48 (69.41%). Anal. Calcd for C_24_H_21_N_3_O_2_ (383.45): C 75.18, H 5.52, N 10.96%. Found C 75.21, H 5.53, N 10.99%.

#### 3.1.9 (1*E*)-*N*-(4-((*E*)-(4-Nitrophenyl)diazenyl)phenyl)-1-(2-phenylhydrazineylidene)propan-2-imine (12)

Red powder; m.p. = 200–202 °C; *R*_f_ = 0.55 EtOAc/petroleum ether (1.5 : 4). IR (*ν*^\^/cm^−1^): 3272 (NH), 3054 (sp^2^ C–H), 2925 (sp^3^ C–H), 1608, 1596, 1534 (C=N & C=C). ^1^HNMR; *δ* ppm 2.51 (s, CH_3_, 3H), 7.59–7.64 (m, 8H), 7.92 (d, *J* = 6.8 Hz, 2H_Ar_), 8.02 (d, *J* = 8.4 Hz, 2H_Ar_), 8.30 (d, *J* = 8.8 Hz, 2H_Ar_), 13.33 (s, NH, 1H). (EMIS) *m*/*z* (%): 386.48 (M^+^, 10.15%), 291.72 (53.63%), 279.54 (56.97%), 242.15 (64.12%), 239.92 (100%, base peak), 223.94 (59.55%), 179.04 (58.15%), 103.42 (57.91%). Anal. Calcd for C_21_H_18_N_6_O_2_ (386.42): C 65.27, H 4.70, N 21.75%. Found C 65.31, H 4.67, N 21.77%.

#### 3.1.10 1,1′-([1,1′-Biphenyl]-4,4′-diylbis(2-phenyl-2,3,4,5-tetrahydro-1,2,4-triazine-4,6-diyl))bis(ethan-1-one) (14)

Buff powder; m.p. = 238–240 °C; *R*_f_ = 0.94 EtOAc/petroleum ether (1.5 : 4). IR (*ν*^\^/cm^−1^): 3033 (sp^2^ C–H), 2924, 2853 (sp^3^ C–H), 1659, 1610 (C=O). ^1^HNMR; *δ* ppm 2.35 (s, 2CH_3_, 6H), 4.27 (s, 2NCH_2_C, 4H), 5.30 (s, 2NCH_2_N, 4H), 7.01–7.10 (m, 5H_Ar_), 7.38–7.50 (m, 13H_Ar_). ^13^C NMR; *δ* ppm 23.93 (2C), 44.89 (2C), 61.64 (2C), 115.19 (4C), 117.68 (4C), 123.10 (2C), 127.02 (2C), 127.32 (2C), 129.85 (4C), 132.61 (2C), 139.60 (2C), 144.75 (2C), 147.47 (2C), 195.72 (2C). (EMIS) *m*/*z* (%): 556.87 (M^+^, 32.96%), 287.95 (81.13%), 208.07 (100%, base peak), 201.56 (76.58%), 194.99 (79.44%), 96.76 (75.53%), 90.61 (94.20%), 41.43 (74.11%). Anal. Calcd for C_34_H_32_N_6_O_2_ (556.67): C 73.36, H 5.79, N 15.10%. Found C 73.33, H 5.81, N 15.13%.

#### 3.1.11 4-(6-Acetyl-2-phenyl-2,5-dihydro-1,2,4-triazin-4(3*H*)-yl)-1,5-dimethyl-2-phenyl-1,2-dihydro-3*H*-pyrazol-3-one (15)

Yield = 86%; yellow powder; m.p. = 100–102 °C; *R*_f_ = 0.29 EtOAc/petroleum ether (1 : 4). IR (*ν*^\^/cm^−1^): 3063 (sp^2^ C–H), 2923, 2835 (sp^3^ C–H), 1650, 1629 (C=O). ^1^HNMR; *δ* ppm 2.21 (s, CH_3_C=O, 3H), 2.38 (s, CH_3_C–, 3H), 3.00 (s, CH_3_N–, 3H), 3.95 (s, NCH_2_C, 2H), 4.85 (s, NCH_2_N, 2H), 7.02–7.06 (m, 1H_Ar_), 7.29–7.40 (m, 7H_Ar_), 7.48 (t, *J* = 7.8 Hz, 2H_Ar_). ^13^C NMR; *δ* ppm 10.67, 24.10, 36.57, 45.15, 62.88, 115.29 (2C), 117.65, 122.76, 123.81 (2C), 126.59 (1C), 129.48 (2C), 129.70 (2C), 135.40, 140.61, 144.76, 151.81, 162.56, 195.64. (EMIS) *m*/*z* (%): 389.09 (M^+^, 34.51%), 375.81 (76.32%), 330.70 (100%, base peak), 285.45 (79.14%), 253.70 (73.37%), 164.36 (70.75%), 69.47 (78.13%). Anal. Calcd for C_22_H_23_N_5_O_2_ (389.46): C 67.85, H 5.95, N 17.98%. Found C 67.89, H 5.91, N 18.01%.

#### 3.1.12 (3*Z*,5*E*)-3,5-Bis(2-phenylhydrazineylidene)heptane-2,6-dione (16)

Yield = 49–63%; orange powder; m.p. = 202–204 °C; Lit. m.p.^46^ = 204–205 °C; *R*_f_ = 0.80 EtOAc/petroleum ether (1.5 : 4). ^1^HNMR; *δ* ppm 2.45 (s, 2CH_3_, 6H), 3.75 (s, CH_2_, 2H), 7.03 (t, *J* = 7 Hz, 2H_Ar_), 7.33–7.41 (m, 8H_Ar_), 10.97 (s, 2H, 2NH). ^13^C NMR; *δ* ppm 19.82, 24.68 (2C), 114.52 (4C), 122.91 (2C), 129.99 (4C), 138.16 (2C), 143.46 (2C), 199.32 (2C).

### 3.2 *In vitro* antibacterial assay

Four bacterial strains (*Bacillus subtilis*, *Staphylococcus epidermidis*, and *Enterobacter cloacae*, and *Escherichia coli*) were implemented to assess the antibacterial efficacy of triazine derivatives. *Azithromycin* was adopted as a standard antibacterial agent for this evaluation, while DMSO was used as a negative control. The tested compounds exhibited promising antibacterial activity at a concentration of 20 mg with observable zones of inhibition.

#### 3.2.1 Evaluation of minimal inhibitory concentration (MIC) for compounds 9 and 14

Serial dilutions of the sample in a concentration of 20 mg ml^−1^ for compounds 9 and 14 were used to determine MIC in the nutrient broth medium. The control contained only inoculated broth and was incubated for 24 h at 37 °C. The MIC endpoint is the lowest concentration of the sample where no visible growth is seen in the tubes. The visual turbidity of the tubes was noted, both before and after incubation to confirm the MIC value, and O.D was measured at 600 nm to confirm the result (Table 8).^84^

**Table 8:** Minimum inhibitory concentration (MIC) of compounds 9 and 14

Compound	MIC (mg ml^−1^)			
Strain	*B. subtilis*	*S. epidermidis*	*Entero. Cloacae*	*E. coli*
9	1.87	1.87	1.87	1.87
14	0.62	5	5	5

### 3.3 *In silico* molecular docking

Enzyme regulation studies were conducted using molecular docking to comprehend the observed differences in antibacterial activities among the synthesized compounds while considering the system's complexities. The three-dimensional structure of protein receptors was assessed from PDB (https://www.rcsb.org) and prepared using Discovery Studio, whereas the ligands were constructed with Babel. Then receptors and ligands were subsequently uploaded into AutoDock Vina within PyMOL for docking analysis. Visualization of outcomes in both 2D and 3D formats was accomplished using Discovery Studio, providing a thorough understanding of the interaction dynamics between the ligands and their corresponding protein targets. Details about the bacteria's protein codes and resolutions are illustrated in Table 9.

**Table 9:** Lists the PDB's ID as well as their resolutions for the antibacterial

Bacteria	Gram (+)		Gram (−)	
	*B. subtilis*	*S. epidermidis*	*Entero. Cloacae*	*E. coli*
PDB	1OF0 (ref. 64)	8P20 (ref. 67)	1KQB ^71,72^	1KNZ ^74,75^
Resolution	2.45 Å	2.85 Å	1.80 Å	2.45 Å

## 4 Conclusion

Concisely, this comprehensive study provides an eco-friendly, effective, and wide substrate approach for synthesizing bioactive, relevant triazine scaffolds *via* cascade double Mannich under mild green conditions. Also, we developed a prominent comparison of conventional *versus* ultrasound-assisted one-pot Mannich reactions for the divergent synthesis of functionalized 1,2,4-triazine building blocks, demonstrating that the ultrasound method yielded superior results in terms of both yield and time. The antibacterial activity of the tested compounds was further validated through *in vitro* and *silico* studies, indicating that scaffold 9 has a broad-spectrum against four bacterial types. Regarding tested triazine hybrids, the antibacterial efficacy against four bacterial species was lessened as follows: for *Bacillus subtilis* compound 9 > 10 > 14; for *Staphylococcus epidermidis*, 9 > 14 > 10; for *Enterobacter cloacae*, 9 > 14 > 10>; and *Escherichia coli* the order is 9 > 12 > 15 = 8. Whereas, *in silico* studies demonstrated responsible binding affinity with a binding energy of −7.905, −7.372, −6.880, and −7.544 kcal mol^−1^ for compound 9, sufficient to inhibit crucial 1OF0, 8P20, 1KQB, and 1KZN proteins, respectively. Finally, the optical activity of the synthesized compounds was measured to confirm the regioselective chirality of nitrogenous atoms at positions 2 and 4.

## Data availability

All data and analysis during this study are available in this article and its ESI file.†

## Author contributions

H. A. A.: organic synthesis methodology, software, formal analysis, writing – original draft; M. M. H.: conceptualization, investigation, project administration, editing, validation; M. A. I.: conceptualization, supervision, investigation, project administration, editing, validation; E. A. G.: conceptualization, organic synthesis methodology, formal analysis, investigation, supervision, writing – original draft & editing, project administration; all authors reviewed the manuscript.

## Conflicts of interest

The authors confirm that there are no known competing financial interests or personal relationships associated with this publication for this work that could have influenced its outcome.

## Supplementary Material

RA-015-D5RA01283J-s001
