# Supplementary material for: Sonosynthesis of new functionalized optically active triazines via double Mannich reaction: antibacterial potential and in silico docking study
Source: RSC Adv. 2025 Jun 4;15(22):17516–34. doi: 10.1039/d5ra01283j (PMC12134990; doi:10.1039/d5ra01283j)
Supplement: RA-015-D5RA01283J-s001 [file RA-015-D5RA01283J-s001.pdf]

Supplementary Information (SI) for RSC Advances.
This journal is © The Royal Society of Chemistry 2025

Sonosynthesis of New Functionalized Optically Active Triazines *via* Double Mannich Reaction: Antibacterial Potential and In Silico Docking Study

Hajar A. Ali[a], Mohamed M. Hammouda[a,b], Mohamed A. Ismail[a], Eslam A. Ghaith[a,c*]

(a) Chemistry Department, Faculty of Science, Mansoura University, El-Gomhoria Street, Mansoura 35516, Egypt

(b) Department of Chemistry, College of Science and Humanities in Al-Kharj, Prince Sattam Bin Abdulaziz University, Al-Kharj 11942, Saudi Arabia

(c) Chemistry Department, Faculty of Science, New Mansoura University, New Mansoura City, Egypt.

Corresponding author: ***Eslam A Ghaith*** abdelghaffar@mans.edu.eg, Tel: +2010244410784

Additional file

General experimental:

Melting points (uncorrected) were measured using a Gallenkamp melting-point apparatus and were uncorrected. The reaction mixture was monitored by using thin-layer chromatography (TLC) which was made on silica gel 60 F_{254} precoated aluminum sheets and visualized under ultraviolet (UV) light. Also, Infrared (IR) spectra were recorded on a Thermo Scientific Nicolet iS10 FT-IR Spectrometer. A Bruker 400 *MHz* spectrometers was used for recording ^{1}H-NMR, ^{15}N-NMR, ^{19}F-NMR, ^{13}C-NMR, DEPT (135), HSQC spectra and chemical shifts (δ) were measured in parts per million (ppm) relative to the used DMSO-d_6 as solvent and self-internal standards. All of the exchangeable protons were detected by using D_2O. Electron impact mass spectra were determined at 70 eV on Varian MAT 3311 Kratosinstrument (Micro-analytical center, Faculty of Science, Cairo University. Sonication was performed in a "Spectralab model UCB 40D Ultrasonic cleaning bath" with a frequency of 40 kHz and power 250 W. The reaction flask was located in the cleaner, where the surface of reactants is slightly lower than the level of the water. All chemicals and solvents were used as received from Sigma Aldrich and Fisher scientific companies.

Fig. S1: ^{1}H-NMR spectrum of Compound 1.
Fig. S2: ^{13}C-NMR spectrum of Compound 1.
Fig. S3: IR spectrum of Compound 2.
Fig. S4: ^{1}H-NMR spectrum of Compound 2.
Fig. S5: ^{13}C-NMR spectrum of compound 2.
Fig. S6: Mass spectrum of compound 2.
Fig. S7: ^{1}H-NMR spectrum of compound 3.
Fig. S8: ^{13}C-NMR spectrum of compound 3.
Fig. S9: Mass spectrum of compound 3.
Fig. S10: IR spectrum of compound 6.
Fig. S11: ^{1}H-NMR spectrum of compound 6.
Fig. S12: ^{13}C-NMR spectrum of compound 6.
Fig. S13: ^{15}N-NMR spectrum of compound 6.
Fig. S14: DEPT (135) spectrum of compound 6.
Fig. S15: Mass spectrum of compound 6.
Fig. S16: IR spectrum of compound 8.
Fig. S17: ^{1}H-NMR spectrum of compound 8.
Fig. S18: ^{13}C-NMR spectrum of compound 8.
Fig. S19: Mass spectrum of compound 8.
Fig. S20: IR spectrum of compound 9.
Fig. S21: ^{1}H-NMR spectrum of compound 9.
Fig. S22: ^{19}F-NMR spectrum of compound 9.
Fig. S23: Mass spectrum of compound 9.
Fig. S24: IR spectrum of compound 10.
Fig. S25: ^{1}H-NMR spectrum of compound 10.
Fig. S26: ^{1}H-NMR (D_2O) spectrum of compound 10.
Fig. S27: ^{13}C-NMR spectrum of compound 10.
Fig. S28: Mass spectrum of compound 10.
Fig. S29: IR spectrum of compound 11.
Fig. S30: ^{1}H-NMR spectrum of compound 11.
Fig. S31: ^{13}C-NMR spectrum of compound 11.
Fig. S32: DEPT (135) spectrum of compound 11.
Fig. S33: Mass spectrum of compound 11.
Fig. S34: IR spectrum of compound 12.
Fig. S35: ^{1}H-NMR spectrum of compound 12.
Fig. S36: Mass spectrum of compound 12.
Fig. S37: IR spectrum of compound 14.

Fig. S38: ^{1}H-NMR spectrum of compound 14.
Fig. S39: ^{13}C-NMR spectrum of compound 14.
Fig. S40: Mass spectrum of compound 14.
Fig. S41: IR spectrum of compound 15.
Fig. S42: ^{1}H-NMR spectrum of compound 15.
Fig. S43: ^{13}C-NMR spectrum of compound 15.
Fig. S44: DEPT (135) NMR spectrum of compound 15.
Fig. S45: HSQC spectrum of compound 15.
Fig. S46: Mass spectrum of compound 15.
Fig. S47: ^{1}H-NMR spectrum of compound 16.
Fig. S48: ^{13}C-NMR spectrum of compound 16.
Fig. S49: Depictions of the 2D, 3D structures, and hydrophobic views of tested compounds against 1OF0 protein.
Fig. S50: Binding mode as well as visual interaction of all synthesized compounds with the binding active site of 8P20 protein.
Fig. S51: Molecular docking images of the inhibitors with 1KQB protein.
Fig. S52: Interaction modes of synthesized compounds with *E. coli* protein (PDB: 1KZN).
Fig. S53: Inhibition zone diameter for the designed compounds.

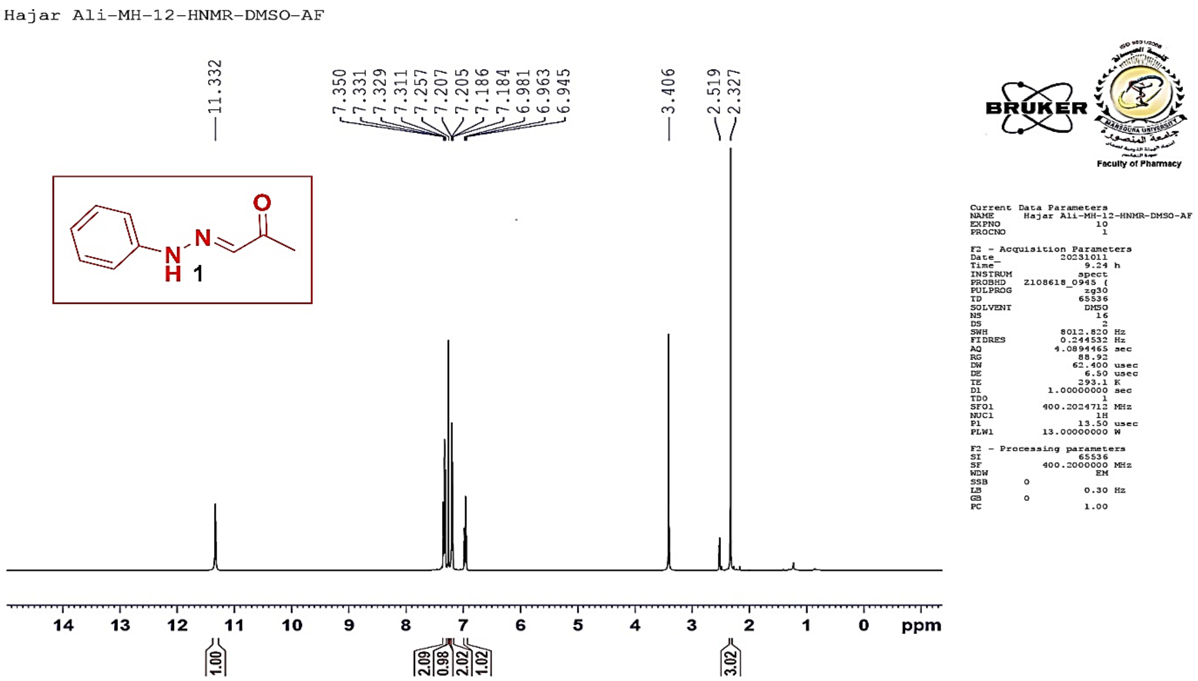

Fig. S1: ^{1}H-NMR spectrum of compound 1

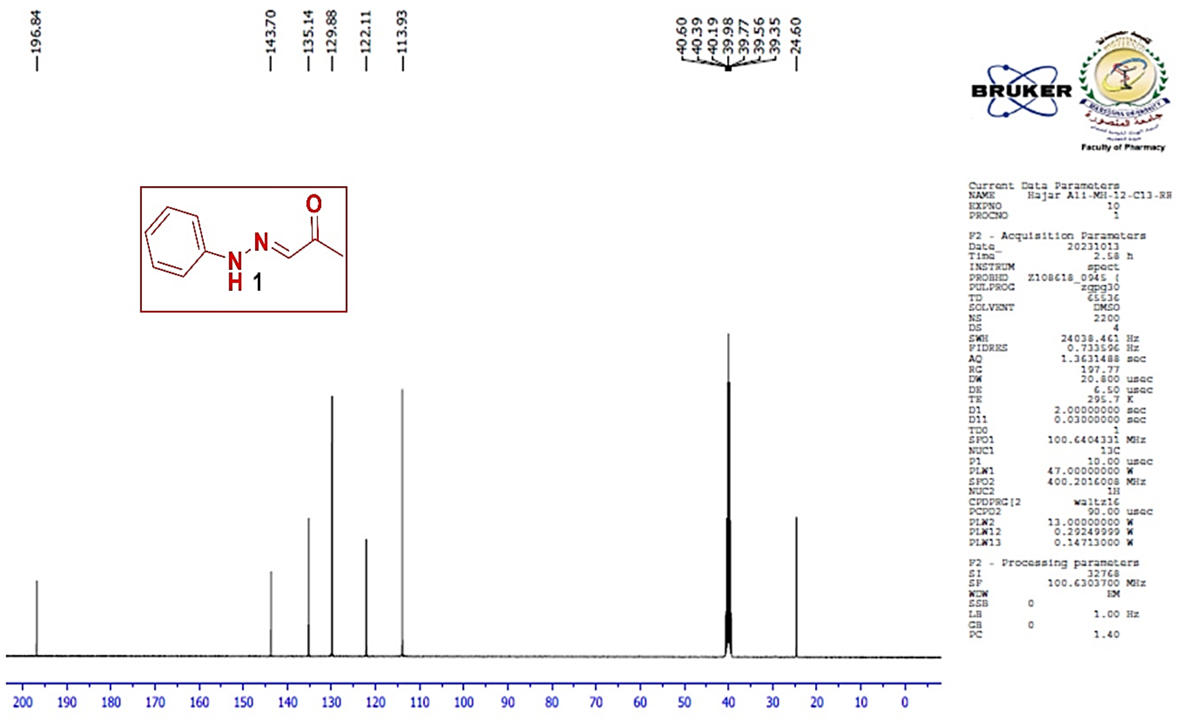

Fig. S2: ^{13}C-NMR spectrum of compound 1

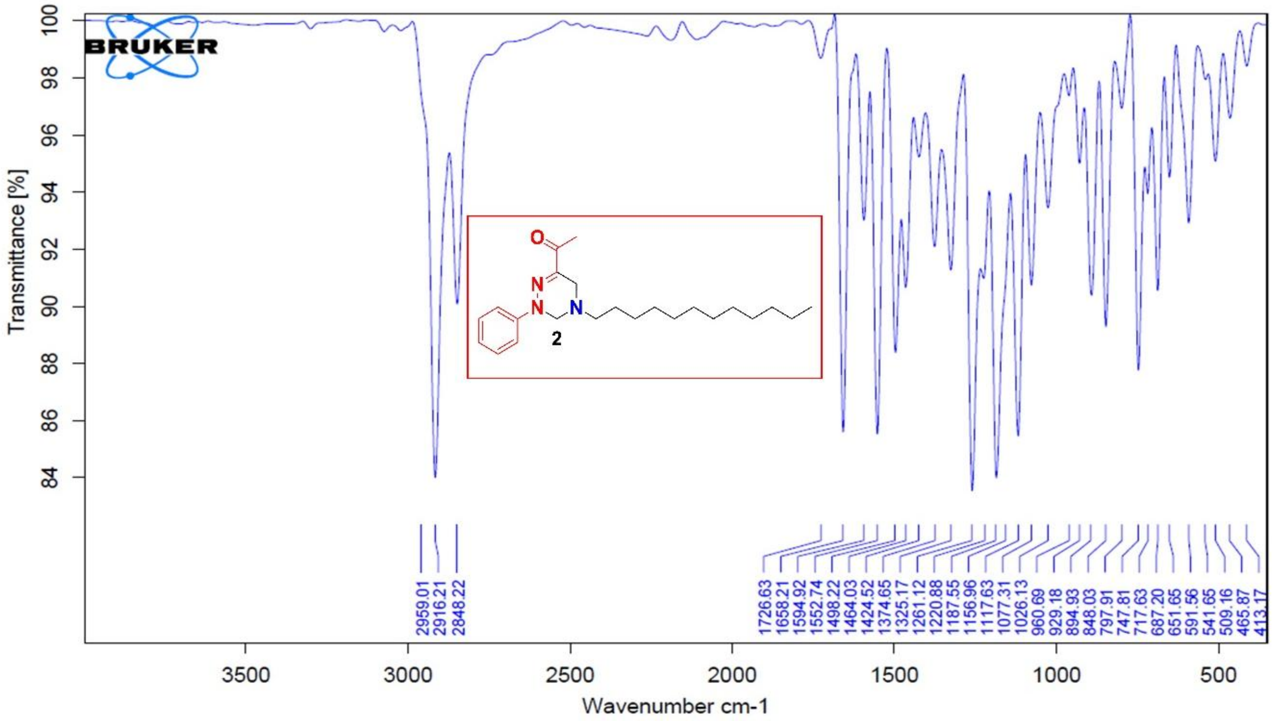

Fig. S3: IR spectrum of Compound 2

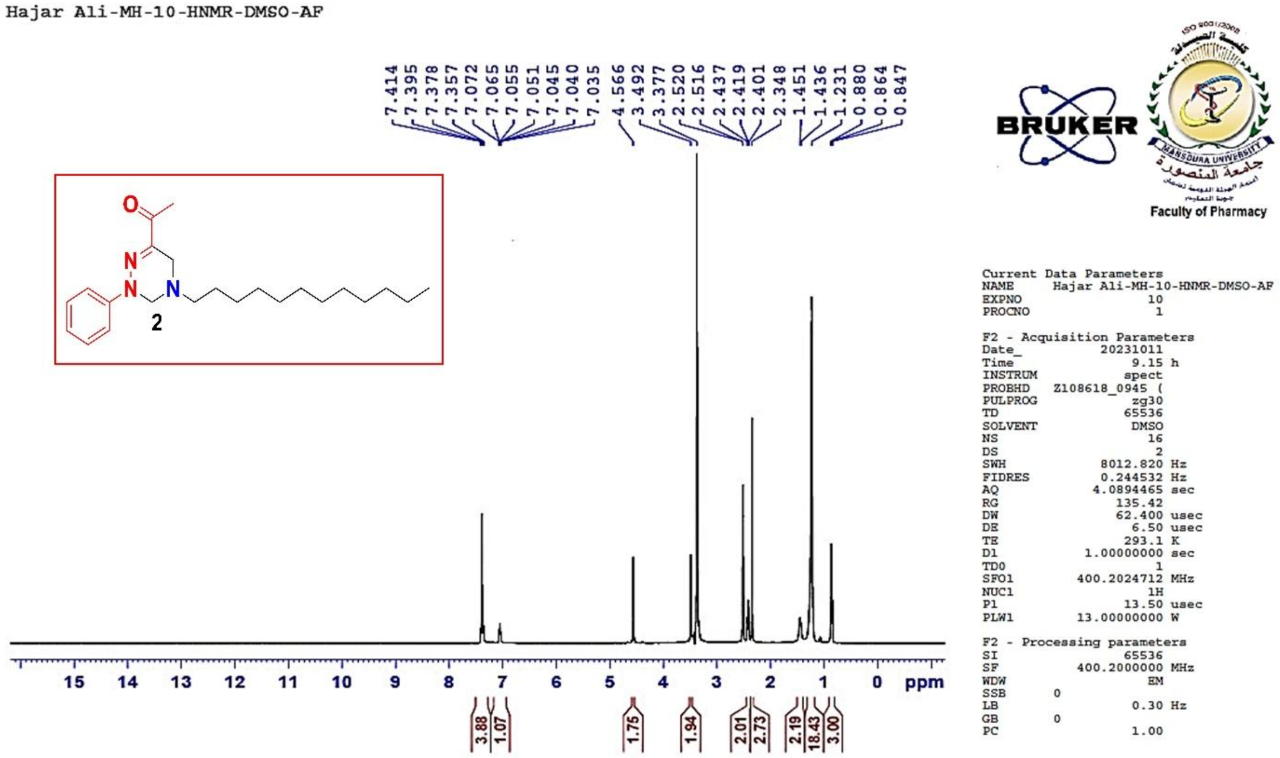

Fig. S4: ^{1}H-NMR spectrum of Compound 2

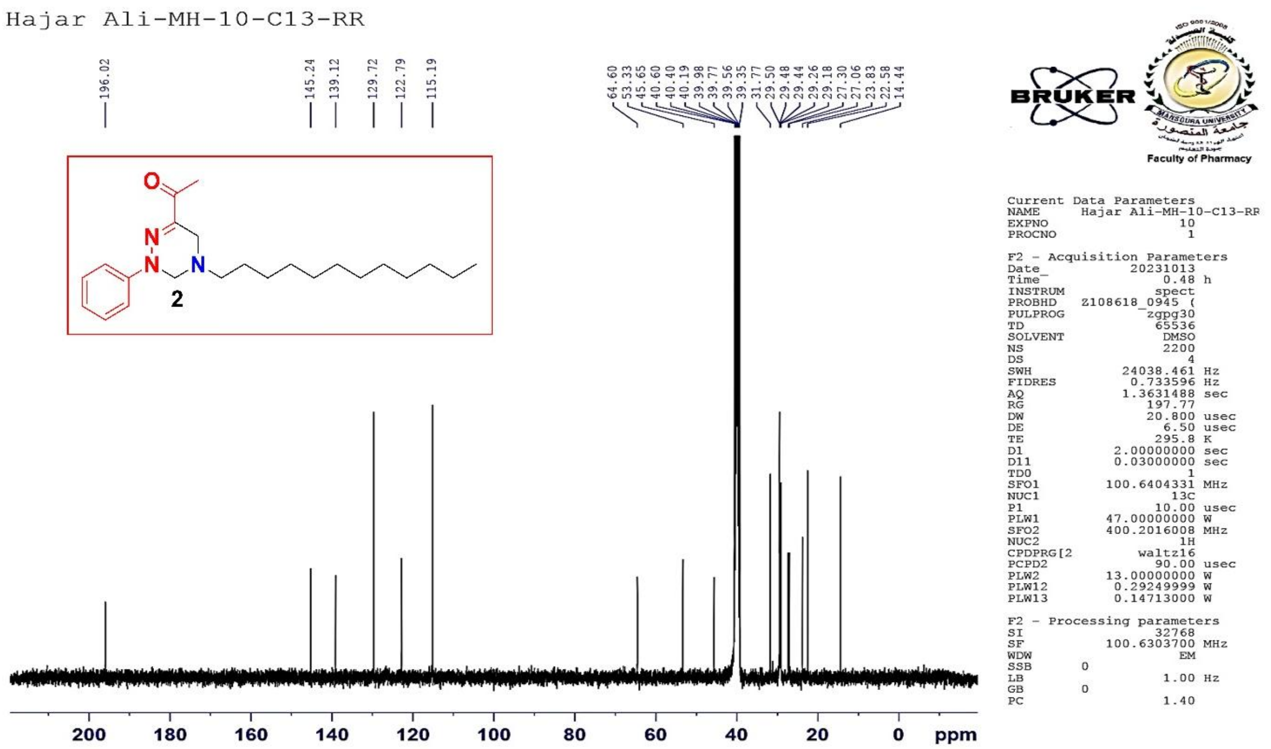

Fig. S5: ^{13}C-NMR spectrum of compound 2

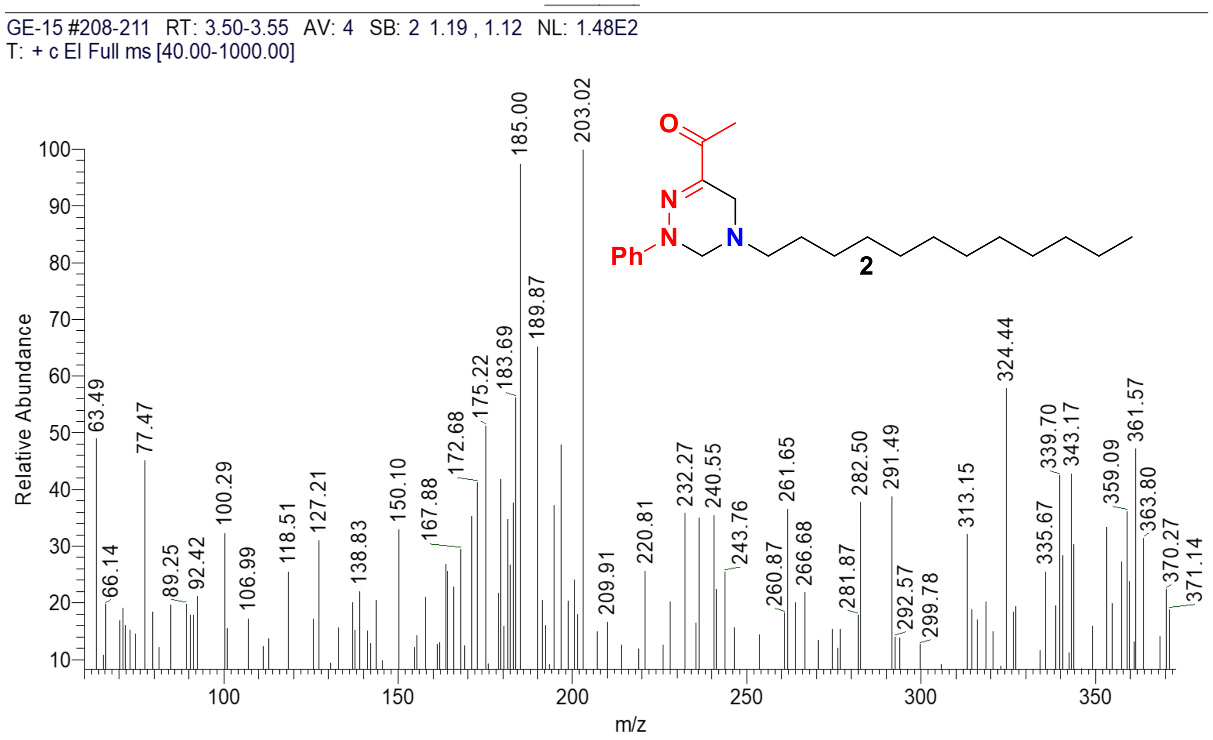

Fig. S6: Mass spectrum of Compound 2

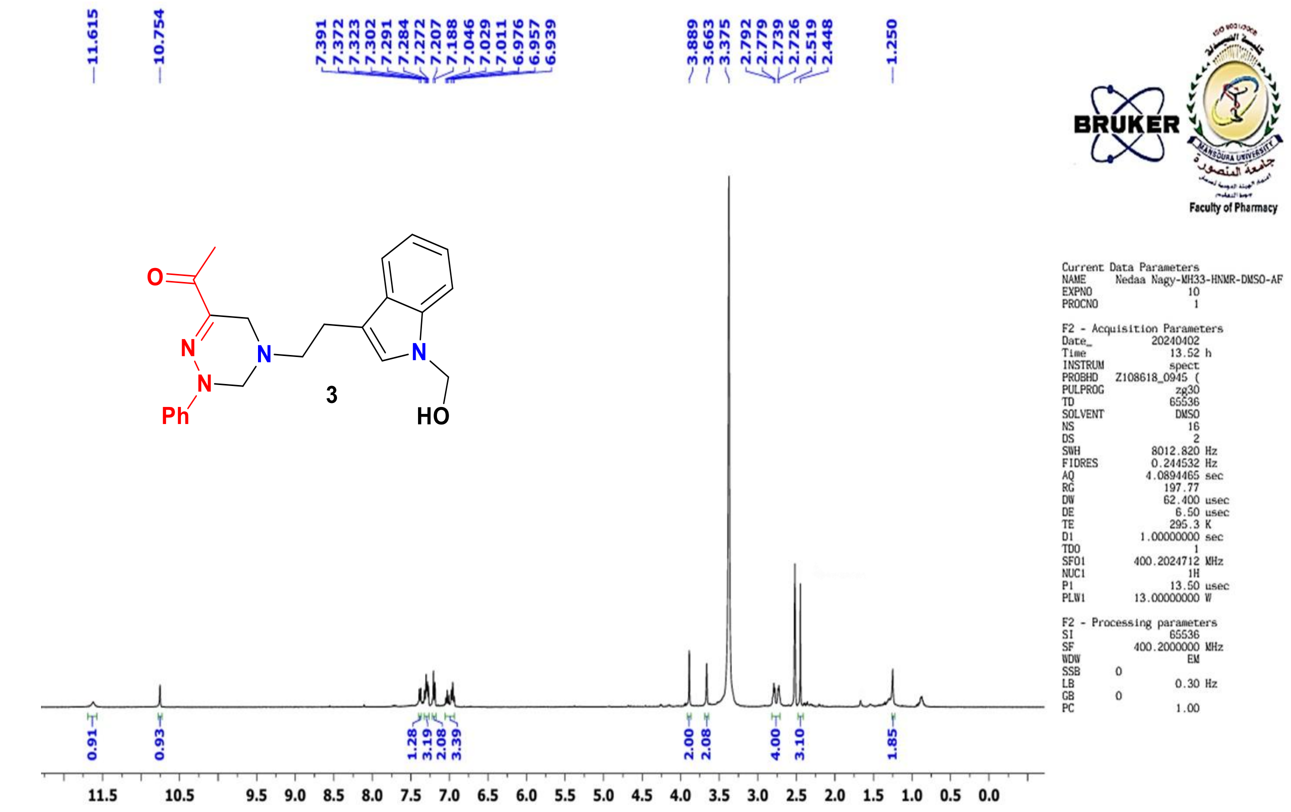

Fig. S7: ^{1}H-NMR spectrum of compound 3

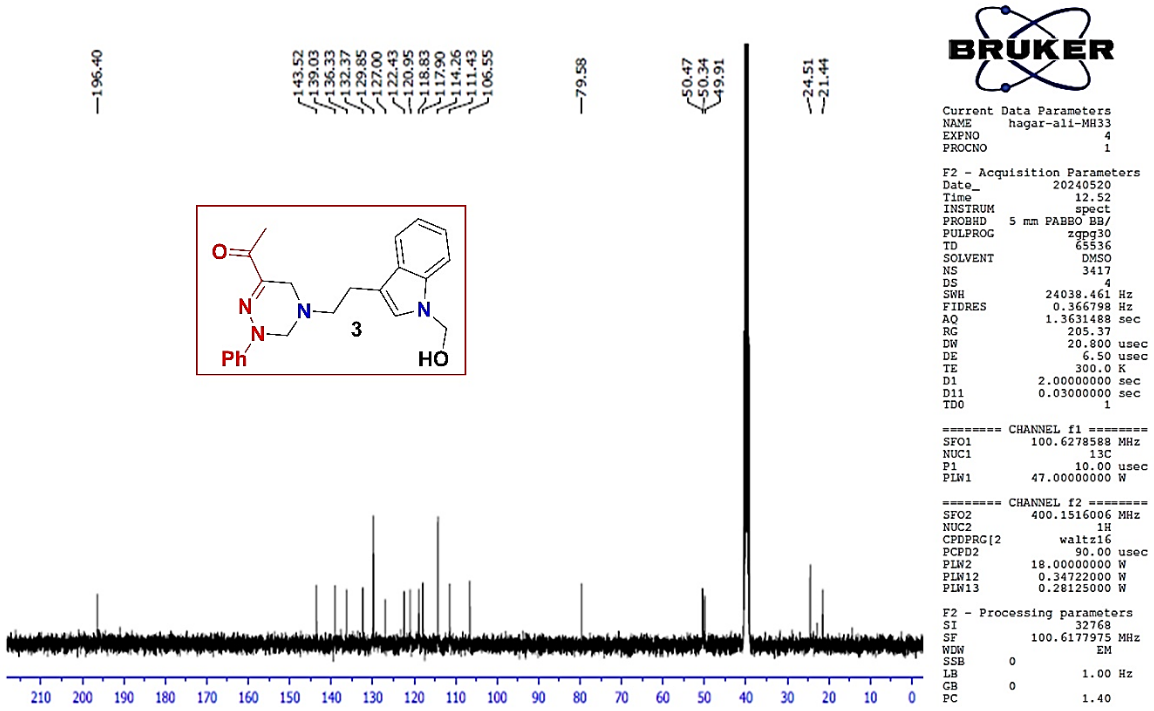

Fig. S8: ^{13}C-NMR spectrum of compound 3

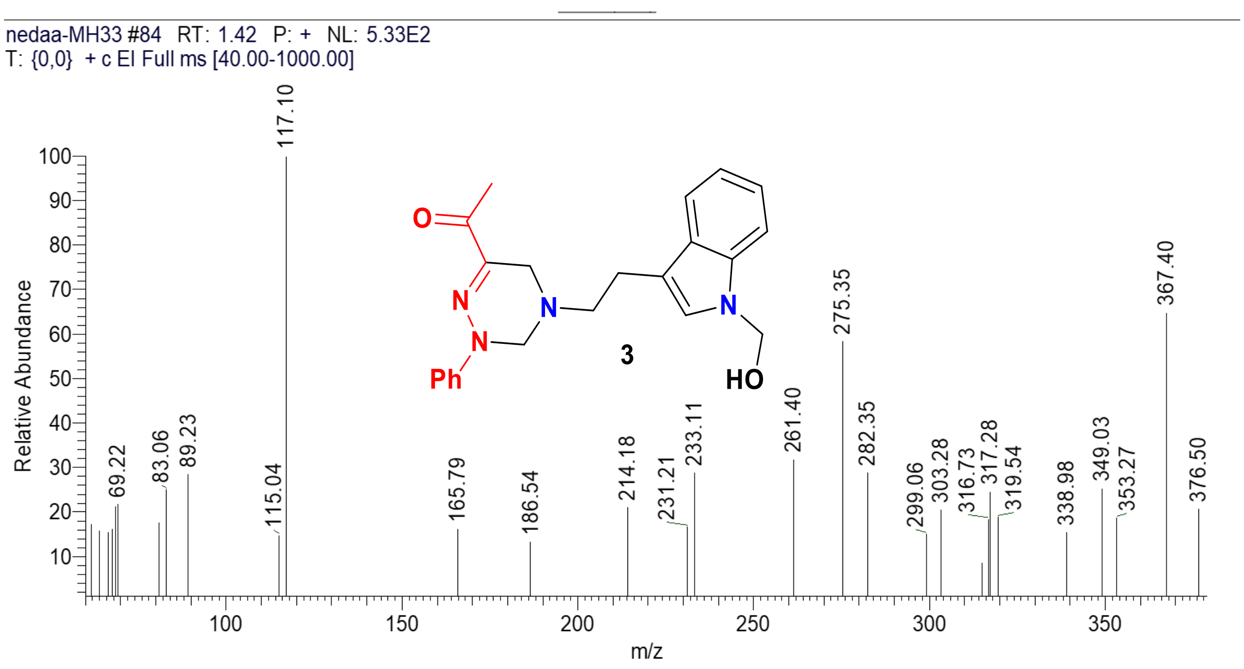

Fig. S9: Mass spectrum of compound 3

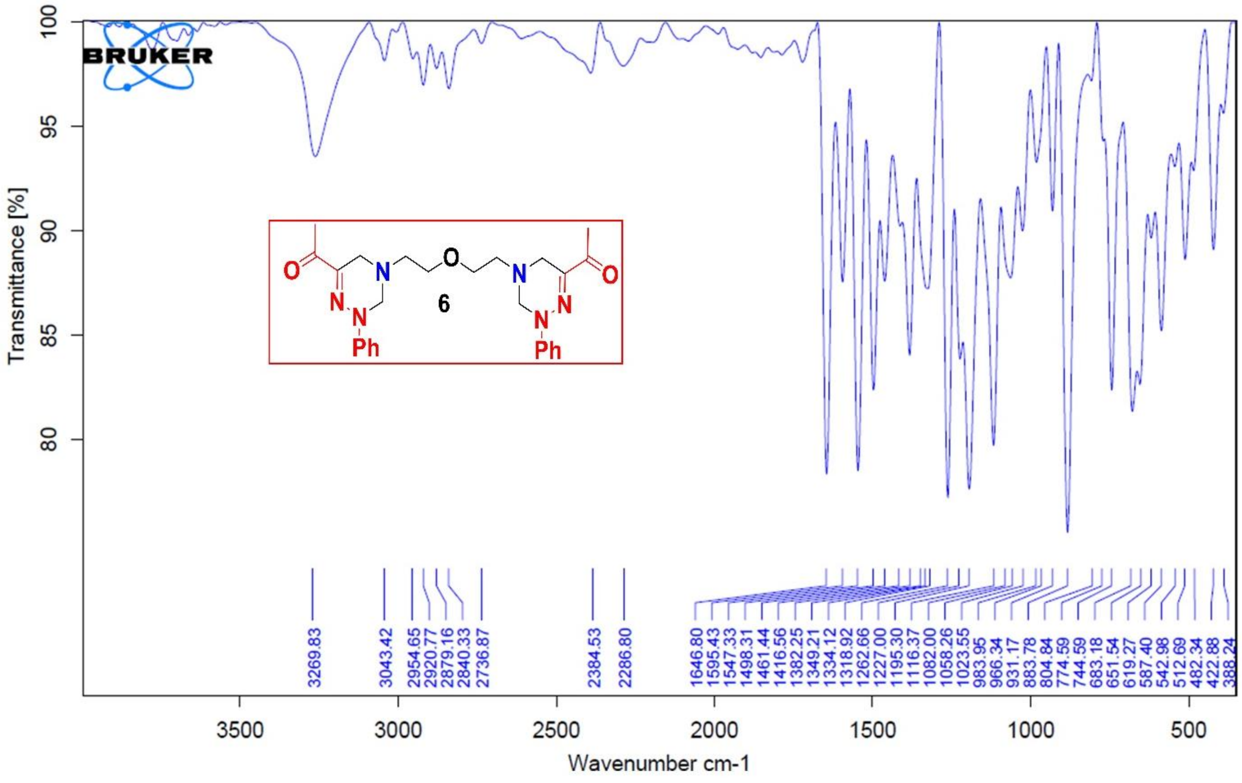

Fig. S10: IR spectrum of compound 6

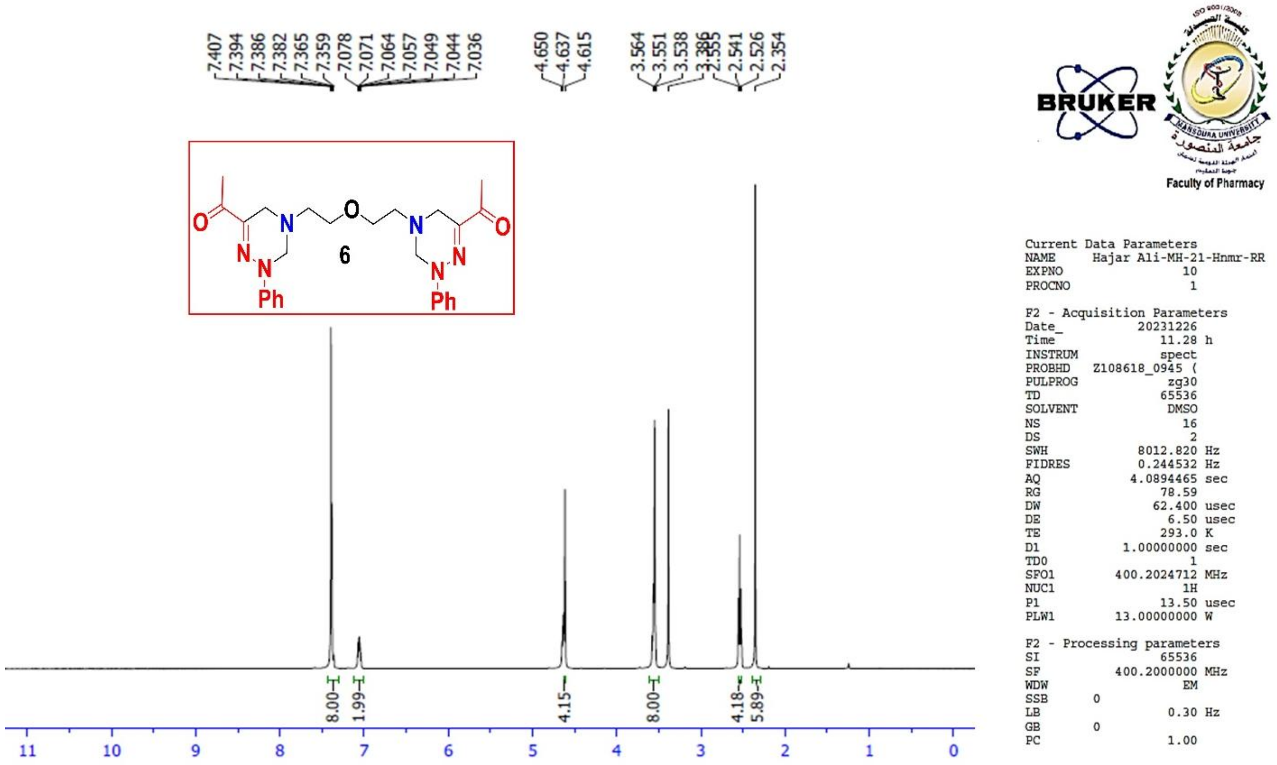

Fig. S11: ^{1}H-NMR spectrum of compound 6

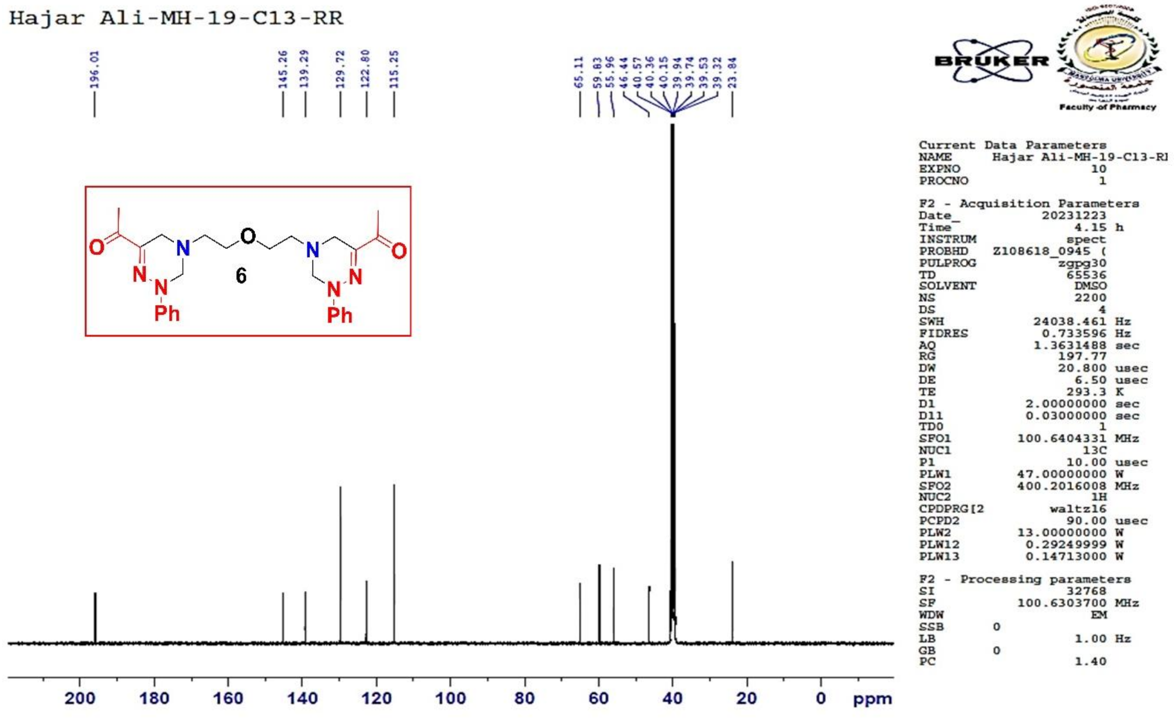

Fig. S12: ^{13}C-NMR spectrum of compound 6

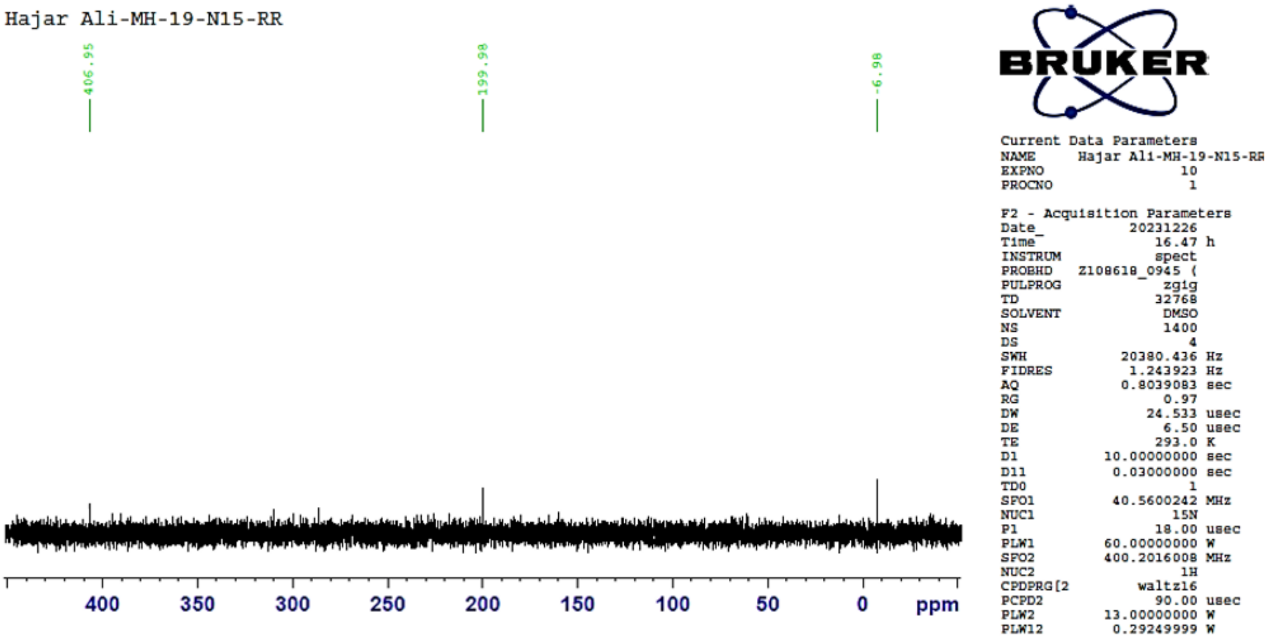

Fig. S13: ^{15}N-NMR spectrum of compound 6

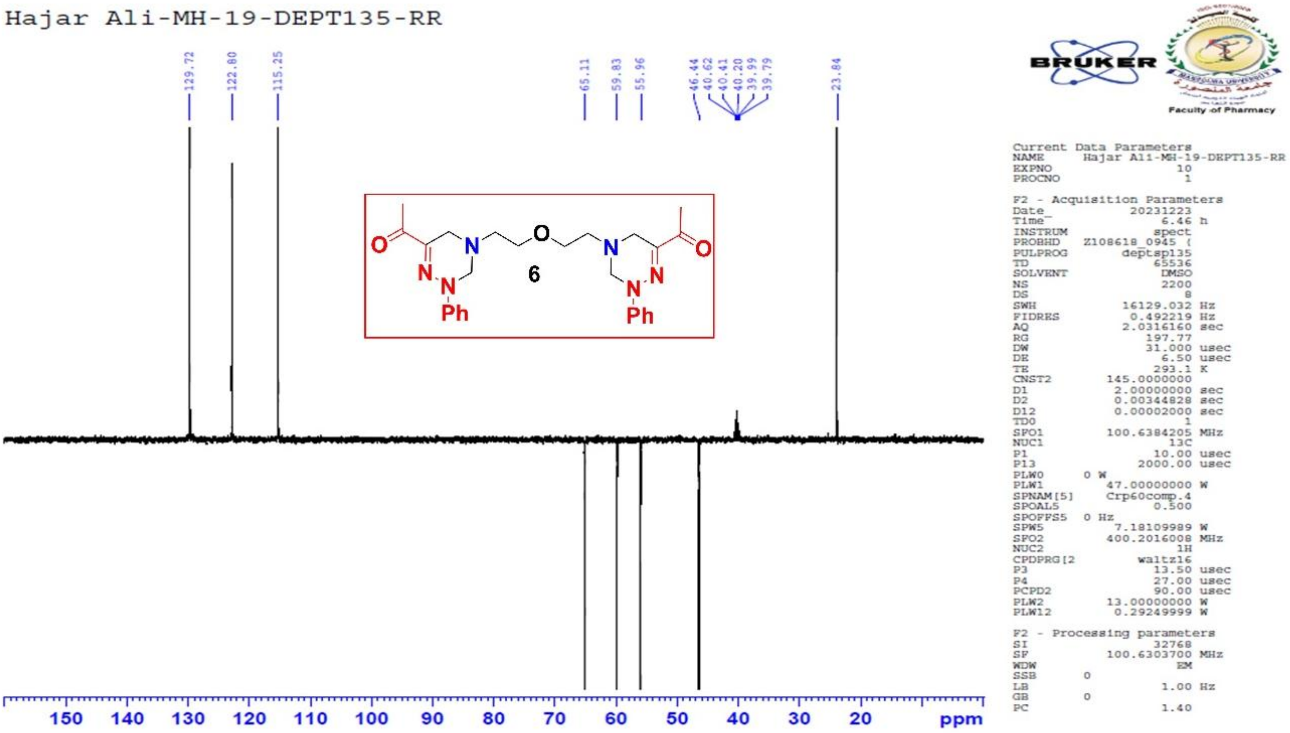

Fig. S14: DEPT (135) spectrum of compound 6.

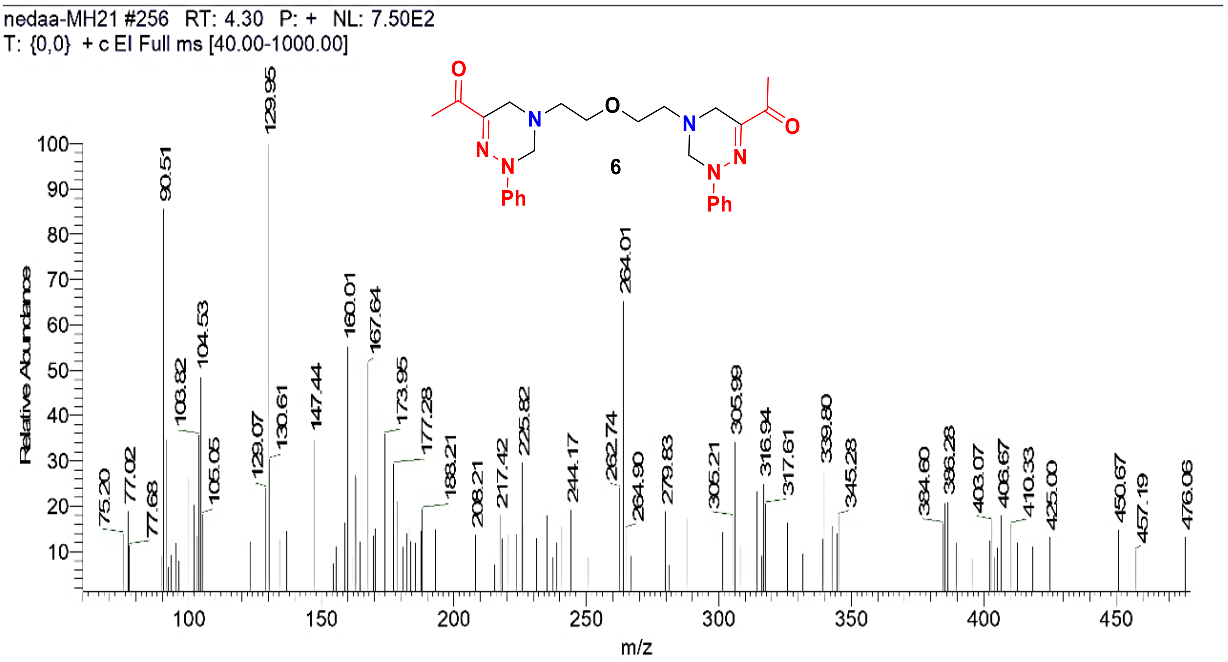

Fig. S15: Mass spectrum of compound 6

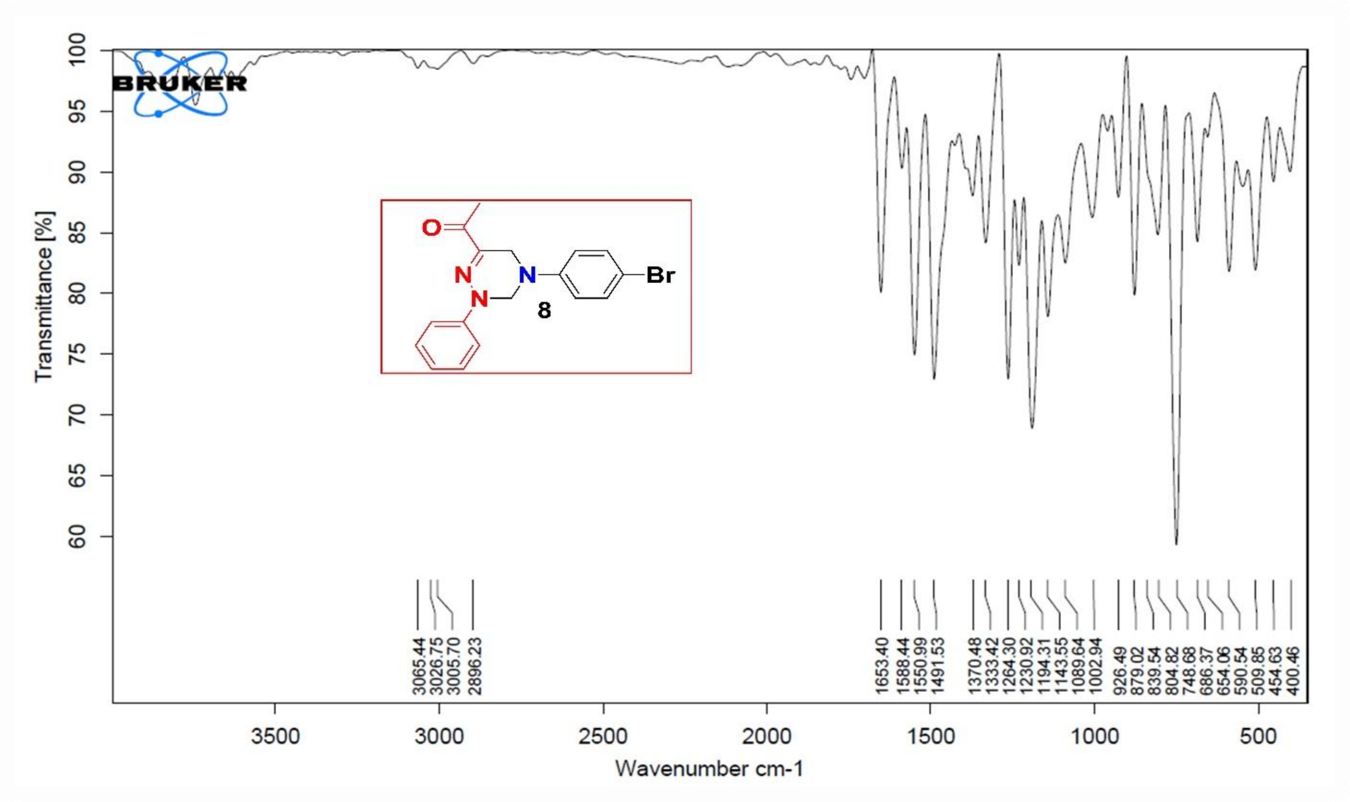

Fig. S16: IR spectrum of compound 8

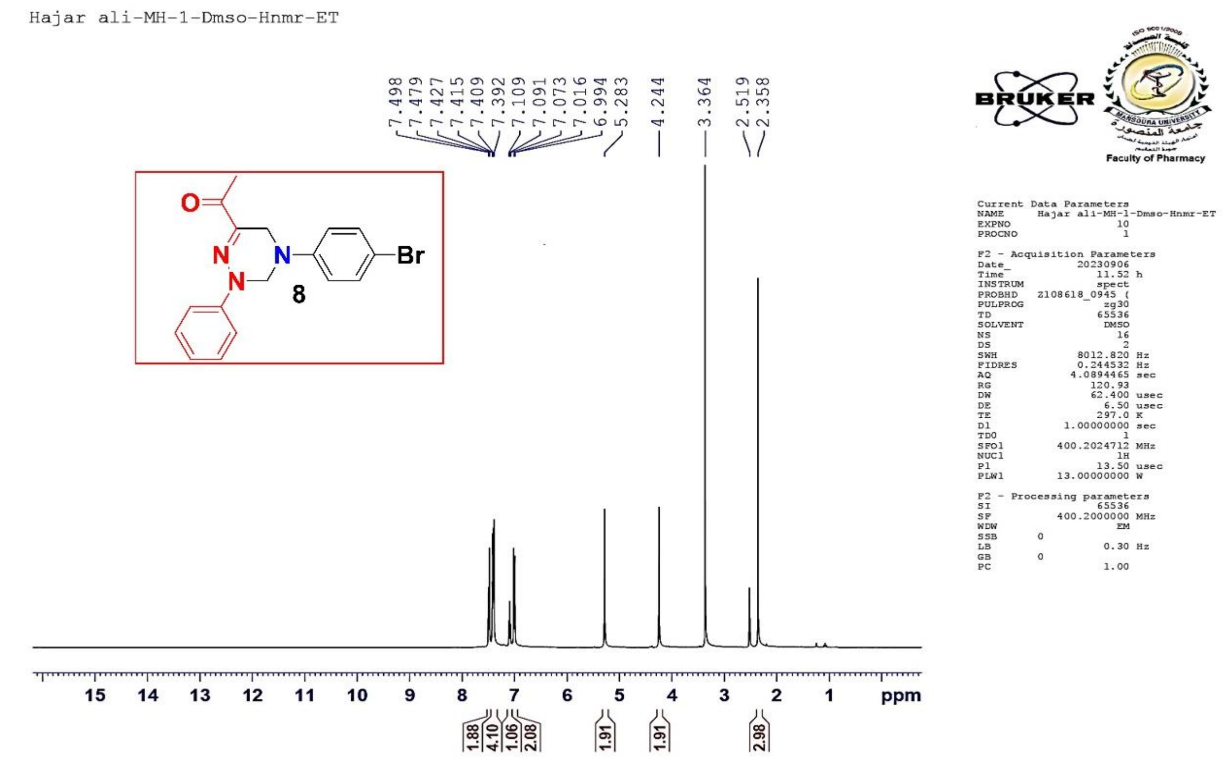

Fig. S17: ^{1}H-NMR spectrum of compound 8

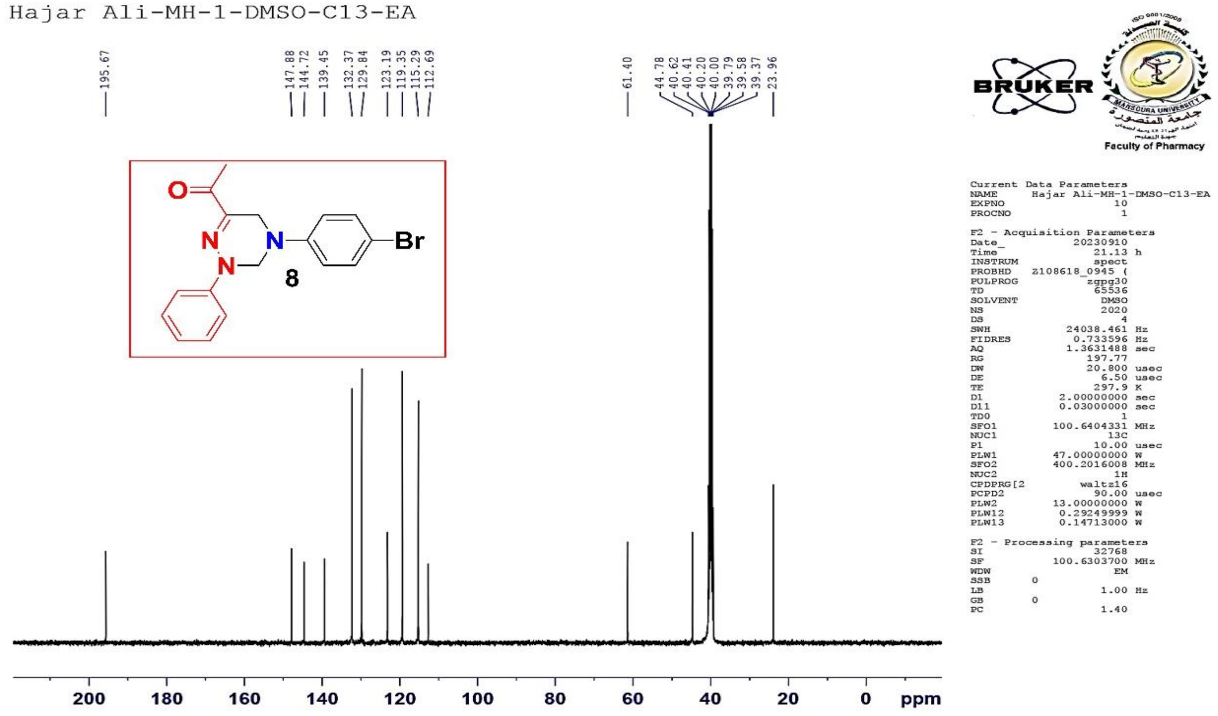

Fig. S18: ^{13}C-NMR spectrum of compound 8

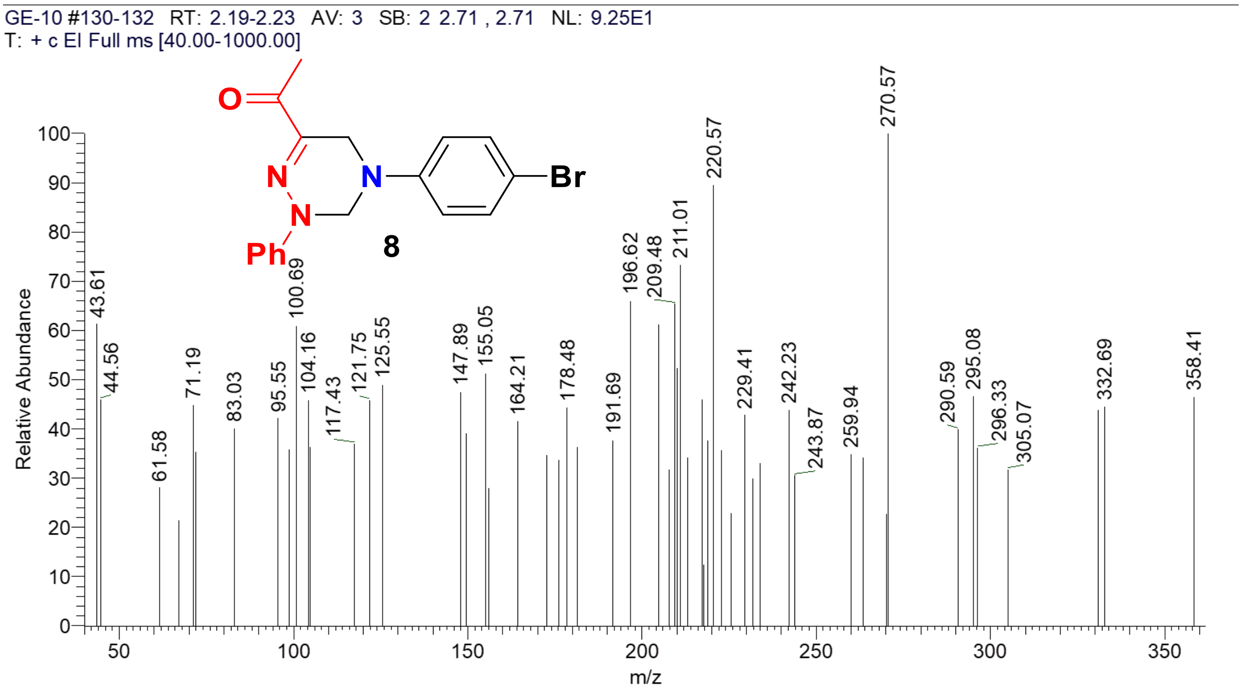

Fig. S19: Mass spectrum of compound 8

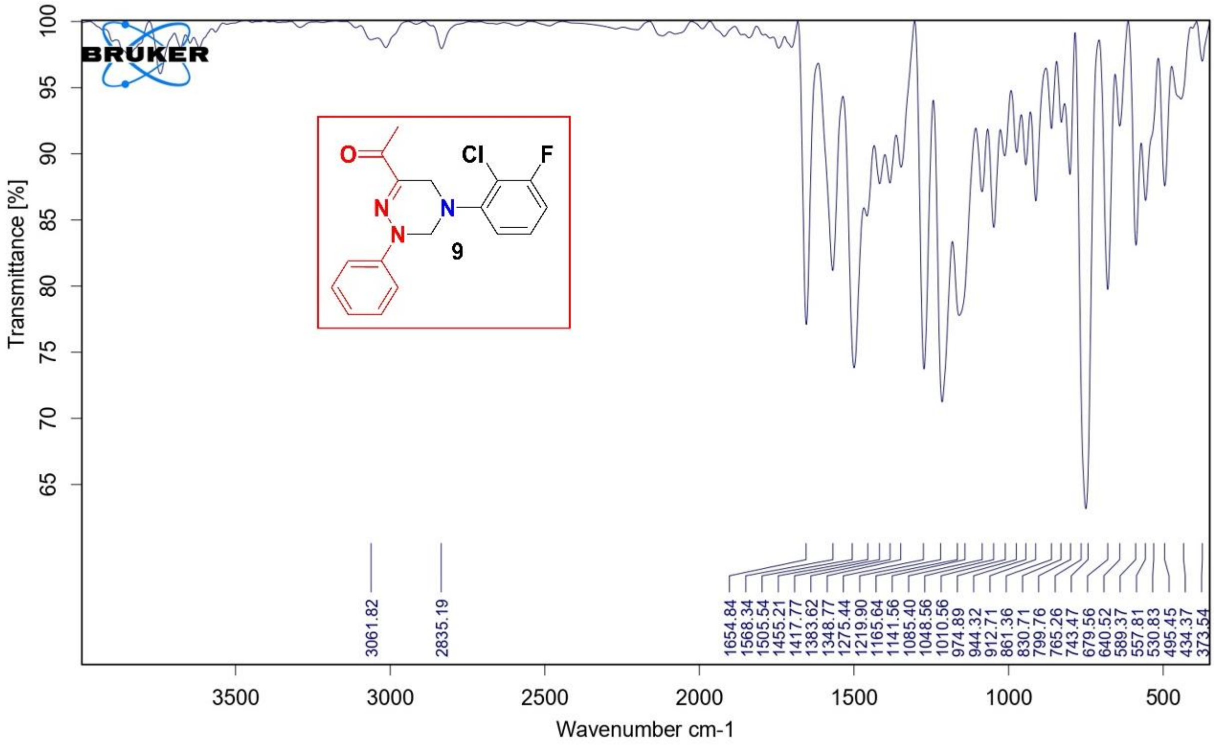

Fig. S20: IR spectrum of compound 9

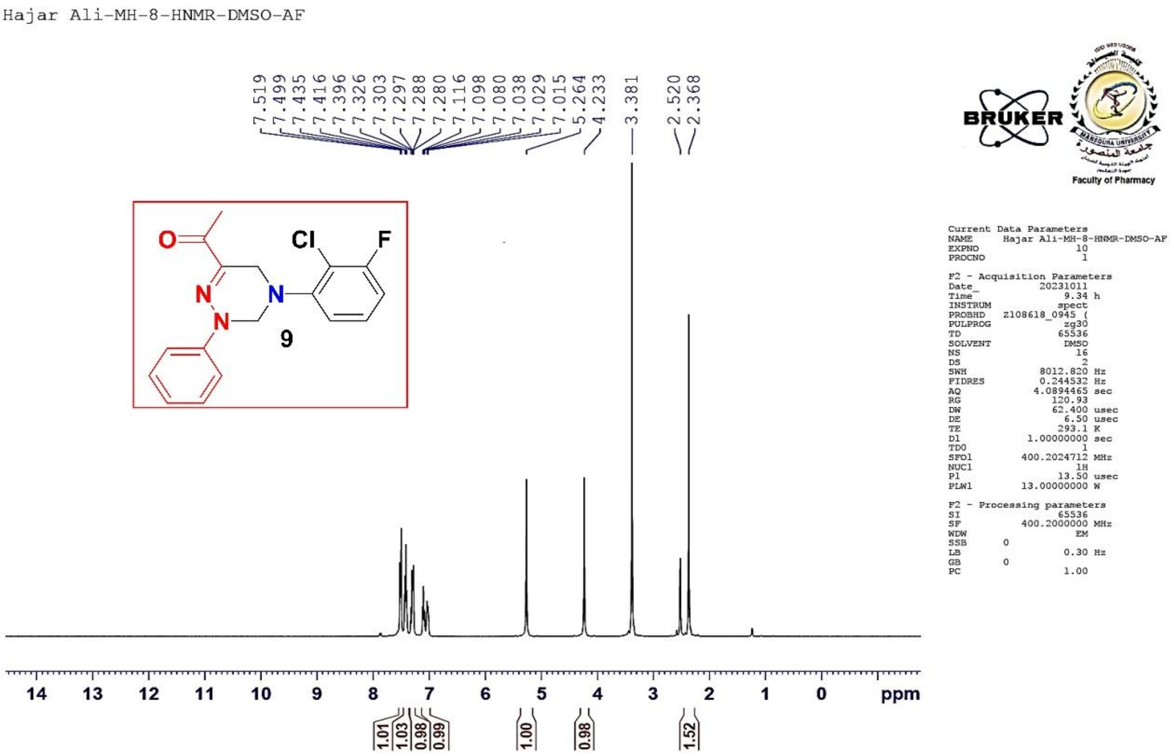

Fig. S21: ^{1}H-NMR spectrum of compound 9

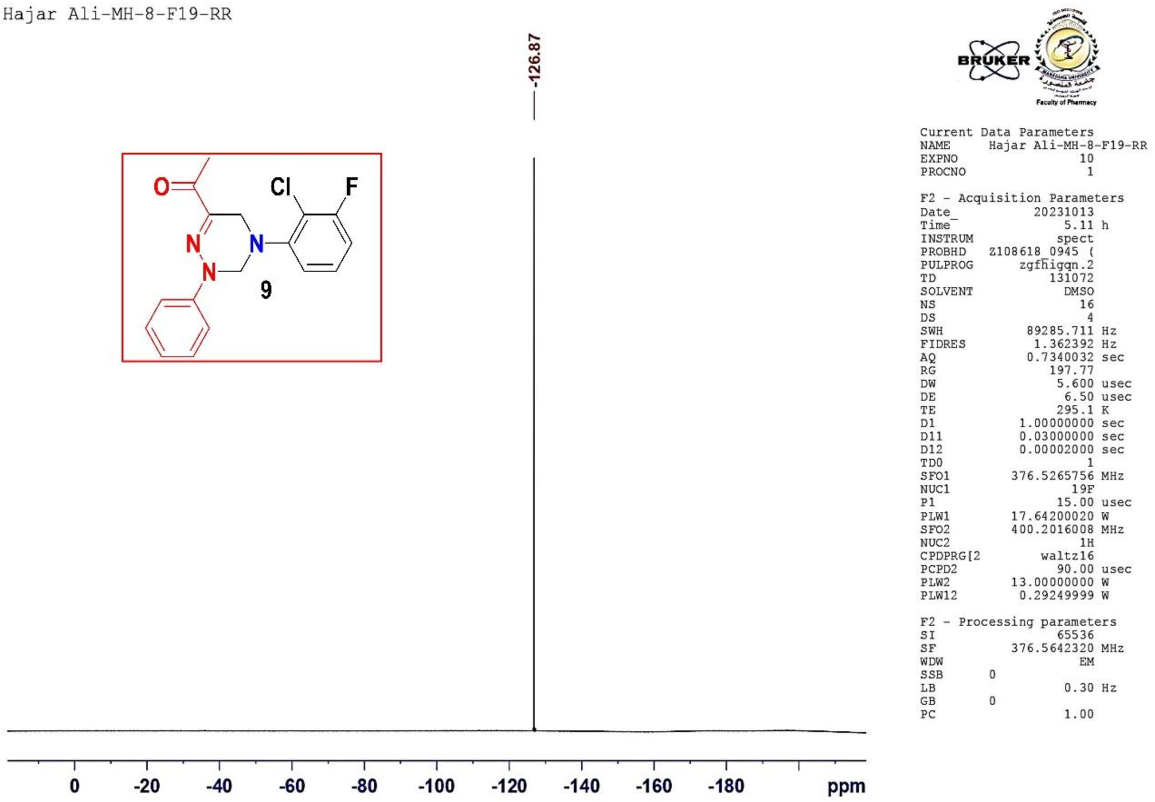

Fig. S22: ^{19}F-NMR spectrum of compound 9

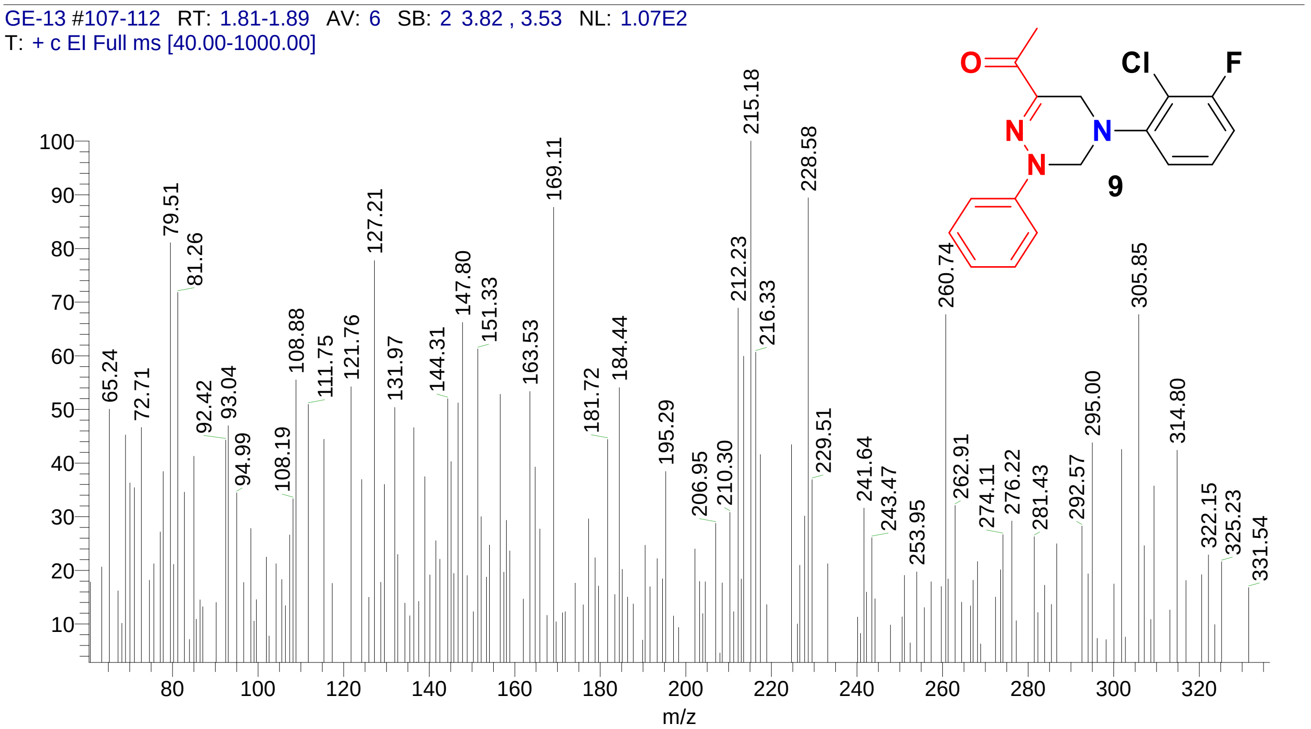

Fig. S23: Mass spectrum of compound 9

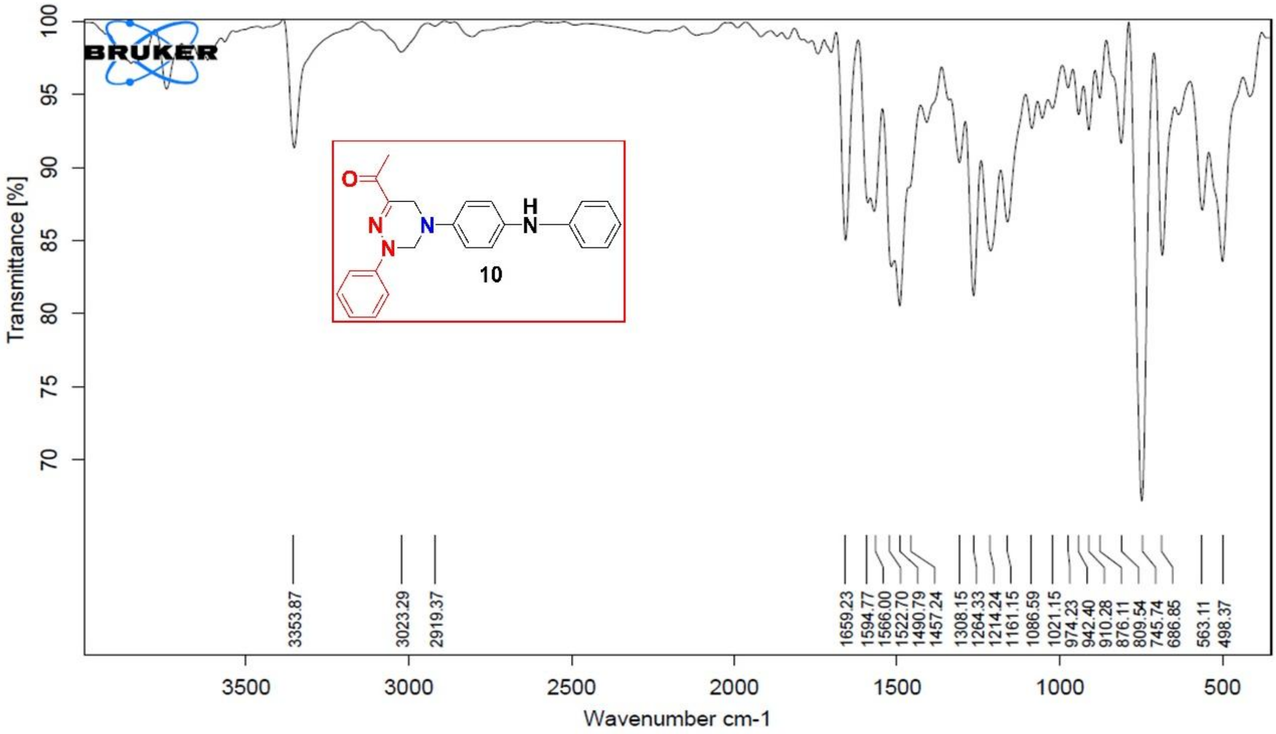

Fig. S24: IR spectrum of compound 10

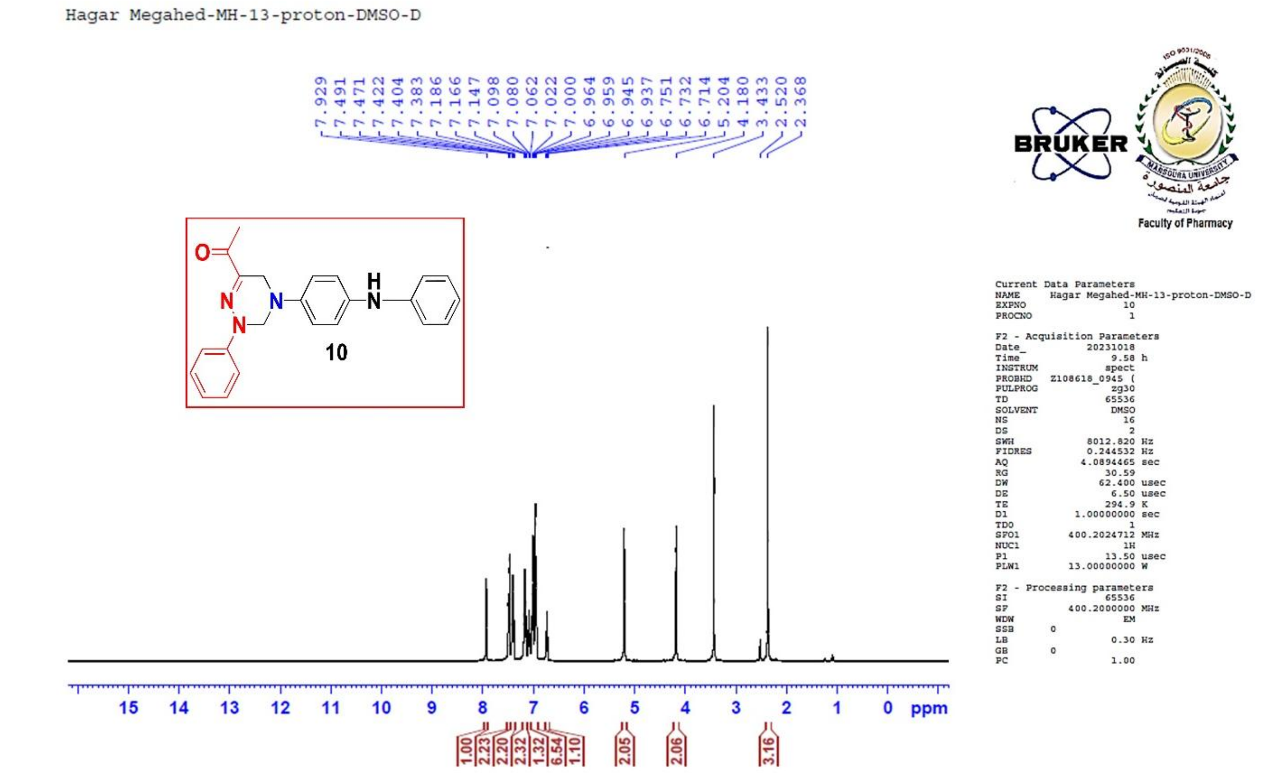

Fig. S25: ^{1}H-NMR spectrum of compound 10

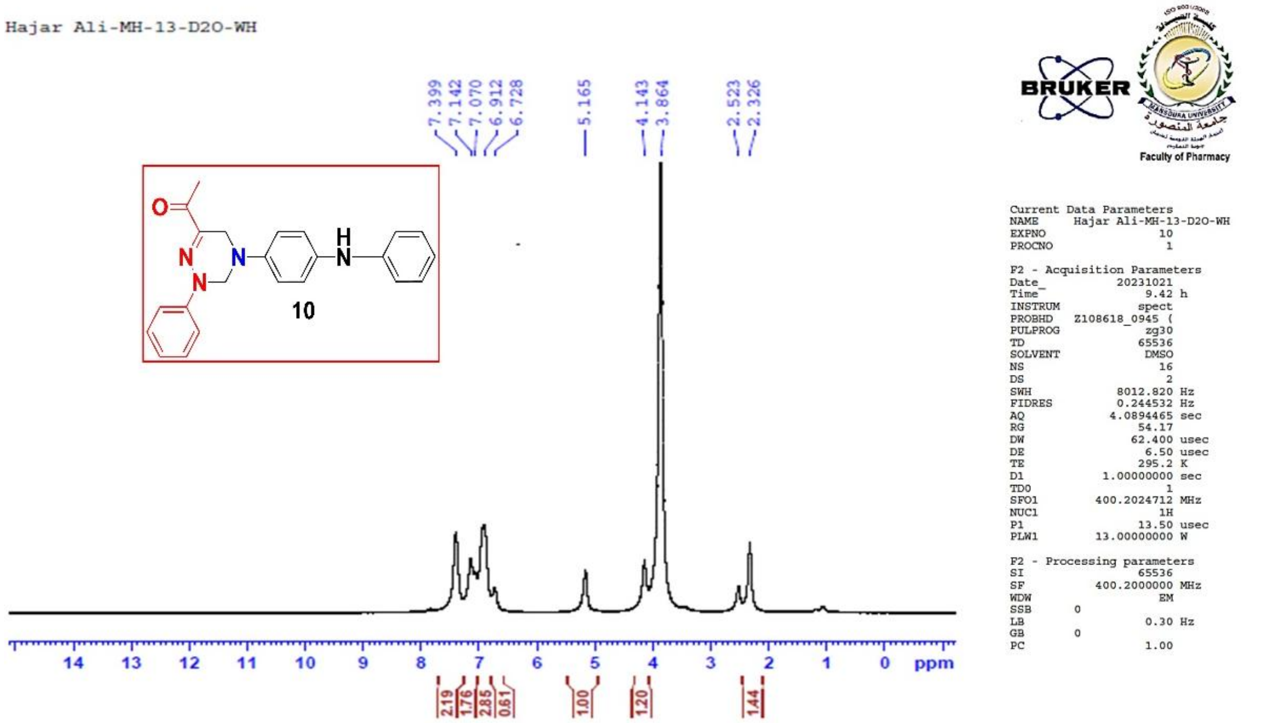

Fig. S26: ^{1}H-NMR (D_2O) spectrum of compound 10

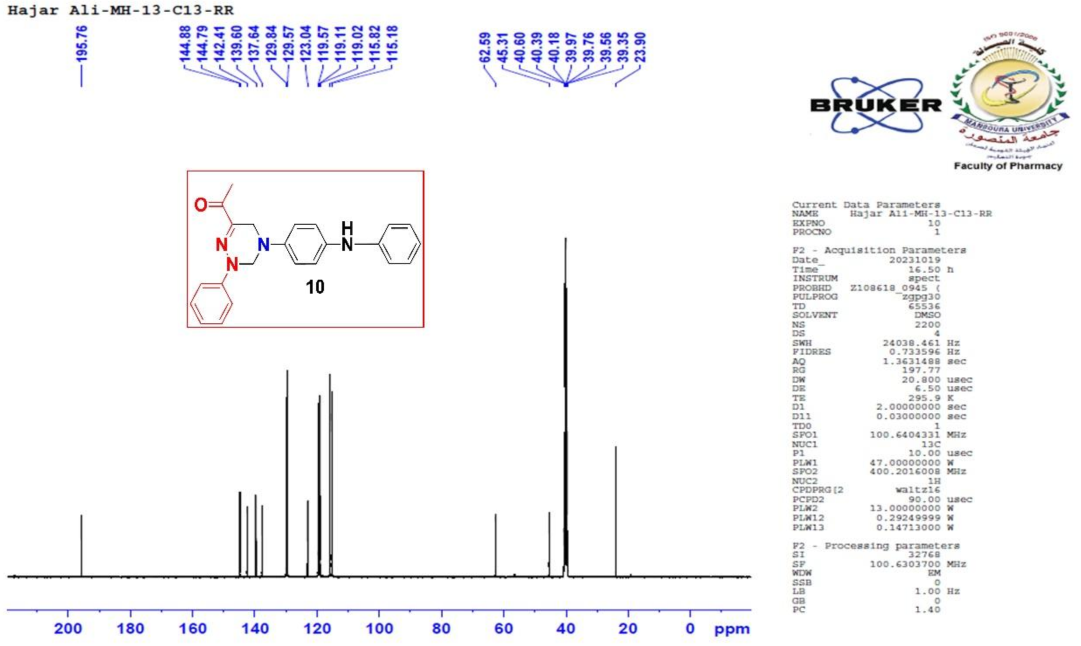

Fig. S27: ^{13}C-NMR spectrum of compound 10

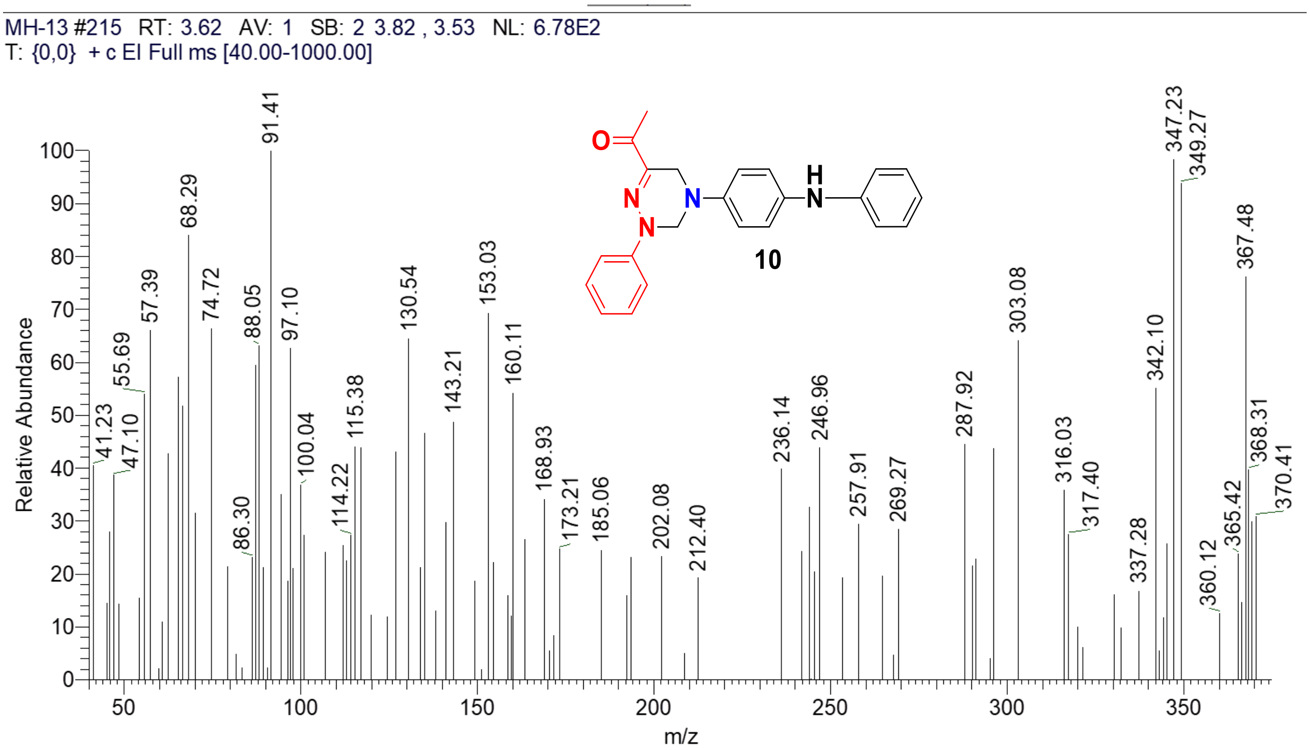

Fig. S28: Mass spectrum of compound 10

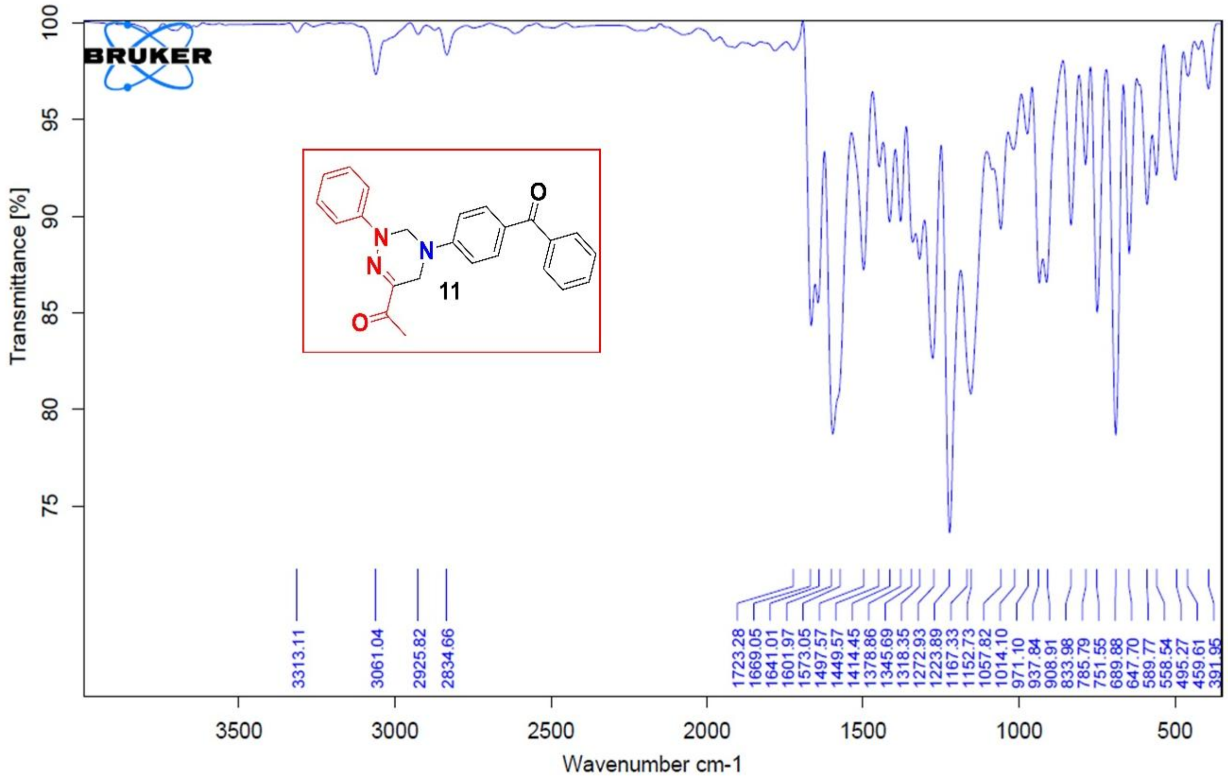

Fig. S29: IR spectrum of compound 11

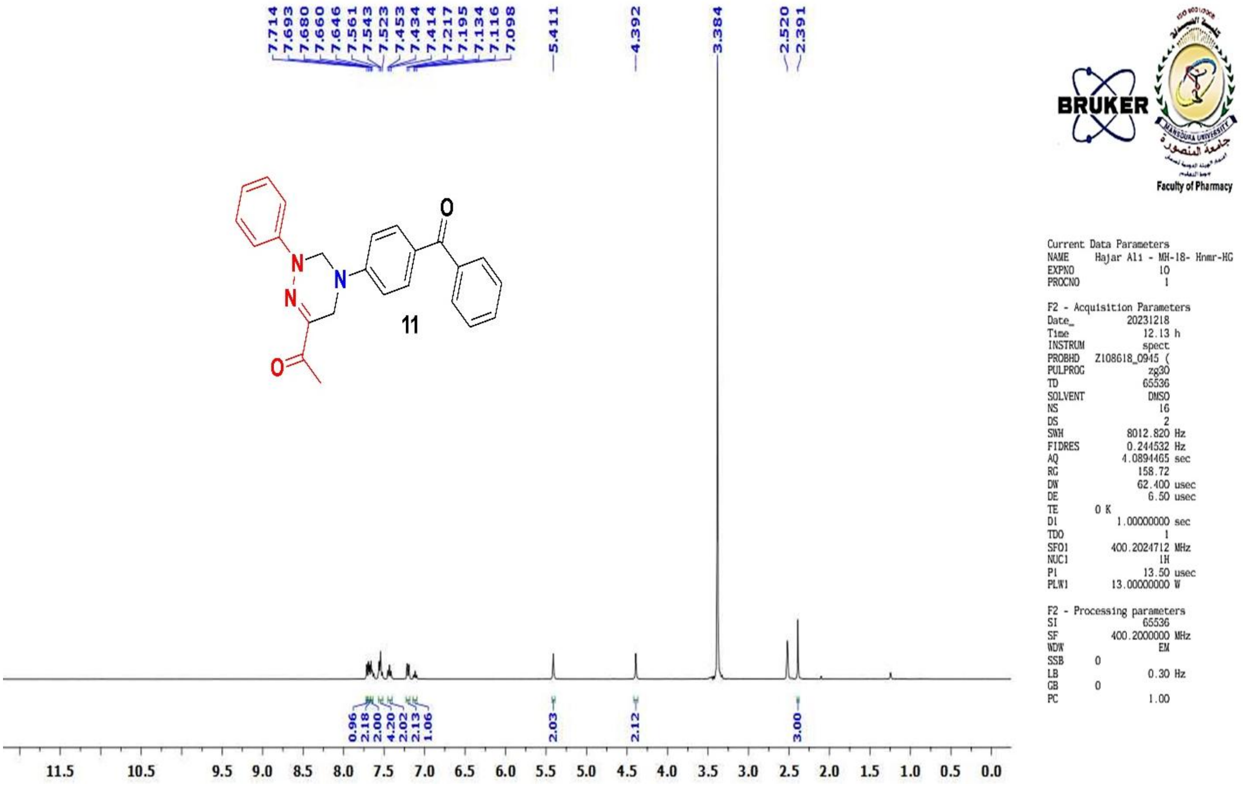

Fig. S30: ^{1}H-NMR spectrum of compound 11

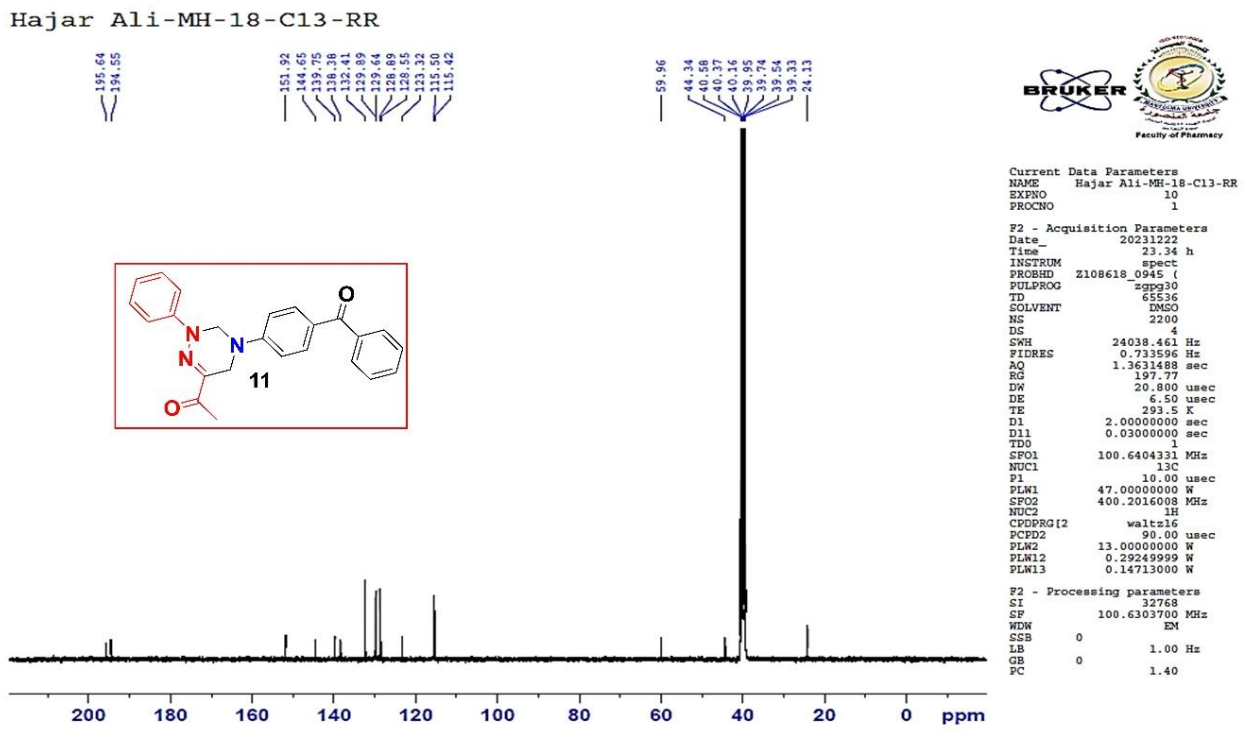

Fig. S31: ^{13}C-NMR spectrum of compound 11

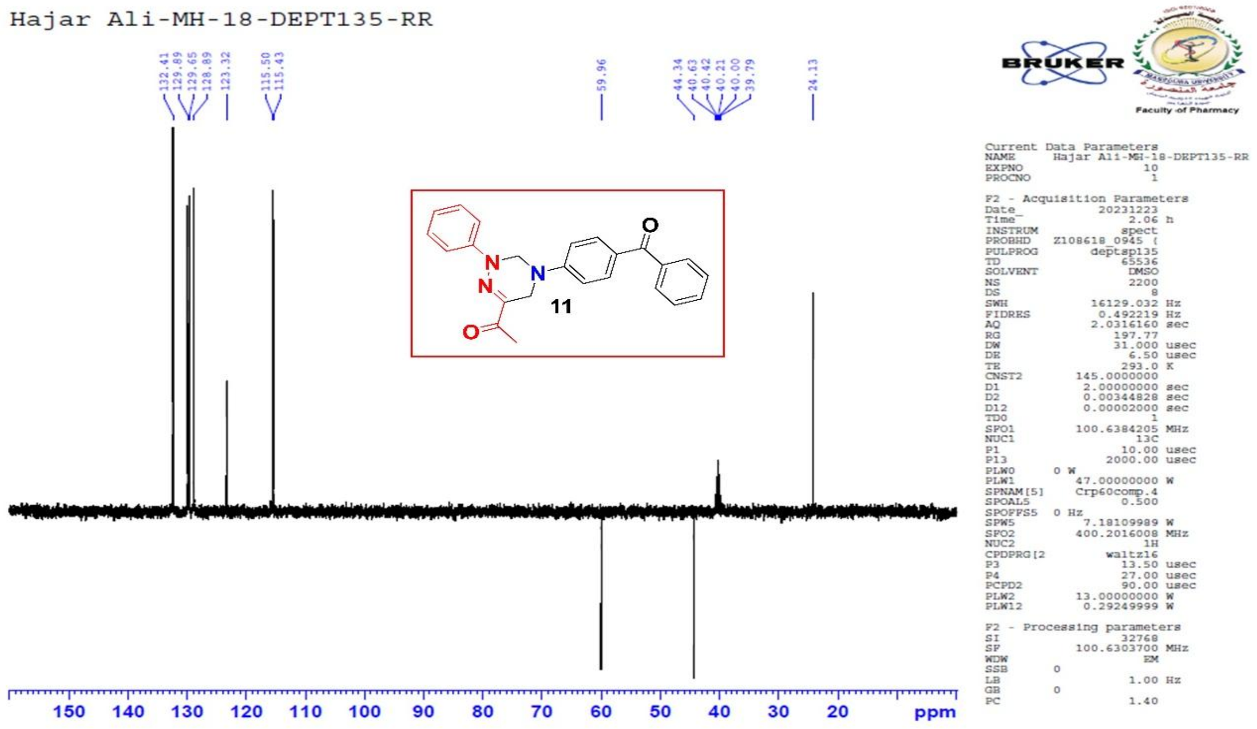

Fig. S32: DEPT (135) spectrum of compound 11

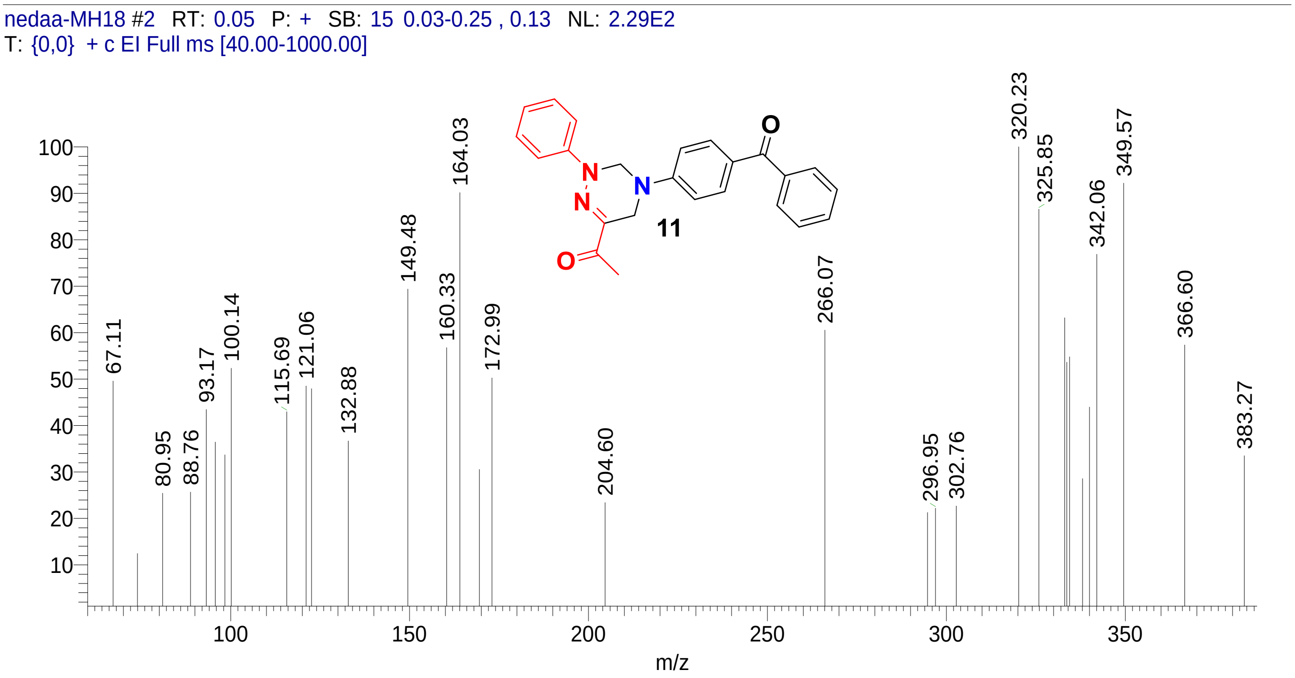

Fig. S33: Mass spectrum of compound 11

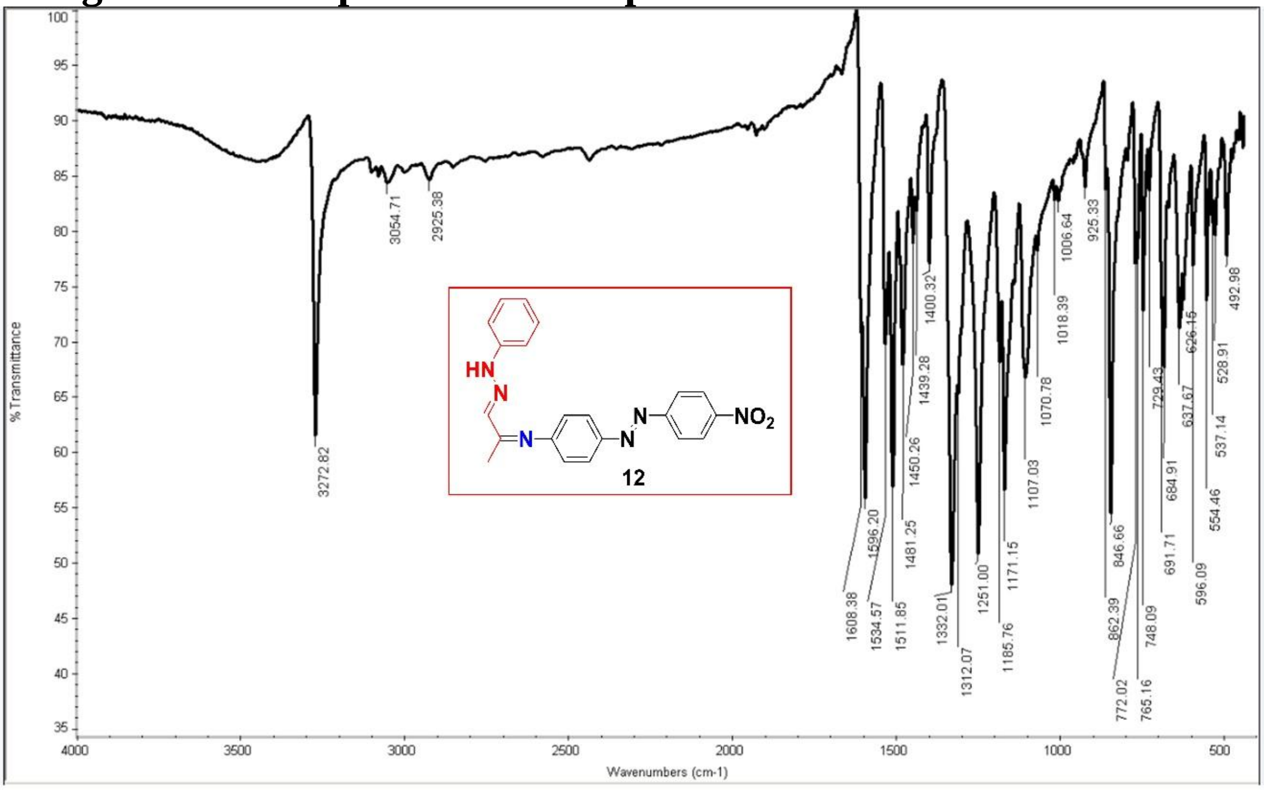

Fig. S34: IR spectrum of compound 12

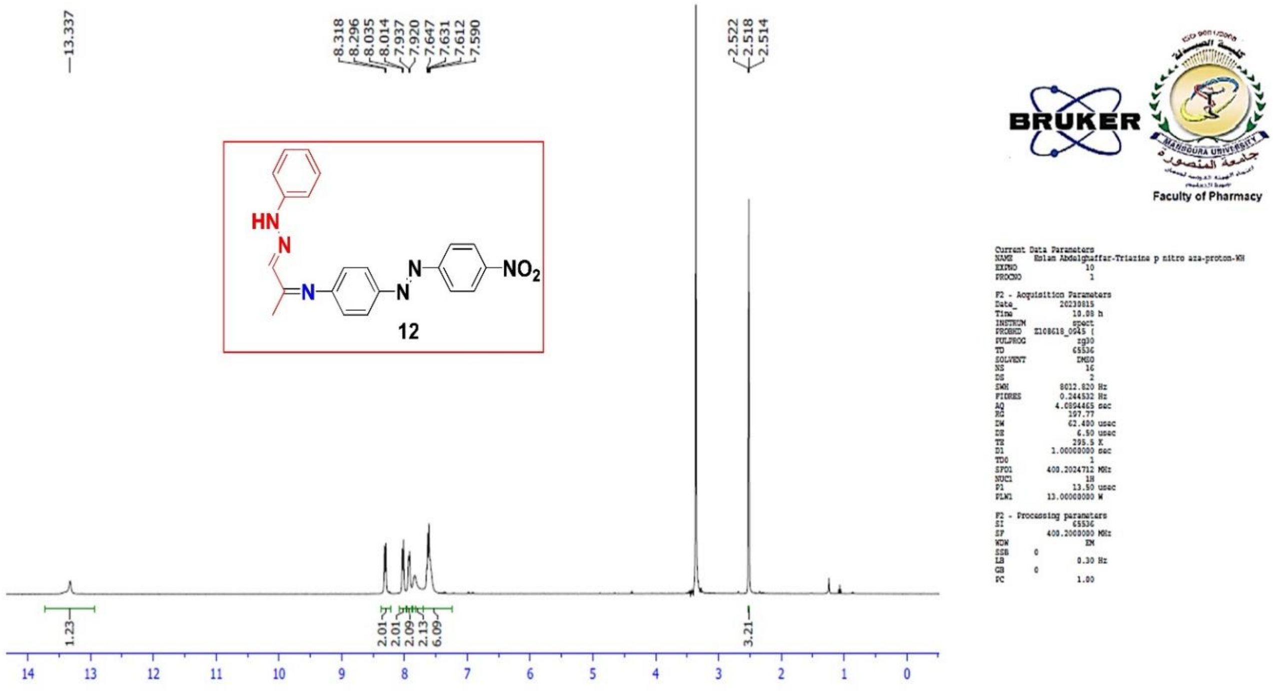

Fig. S35: ^{1}H-NMR spectrum of compound 12

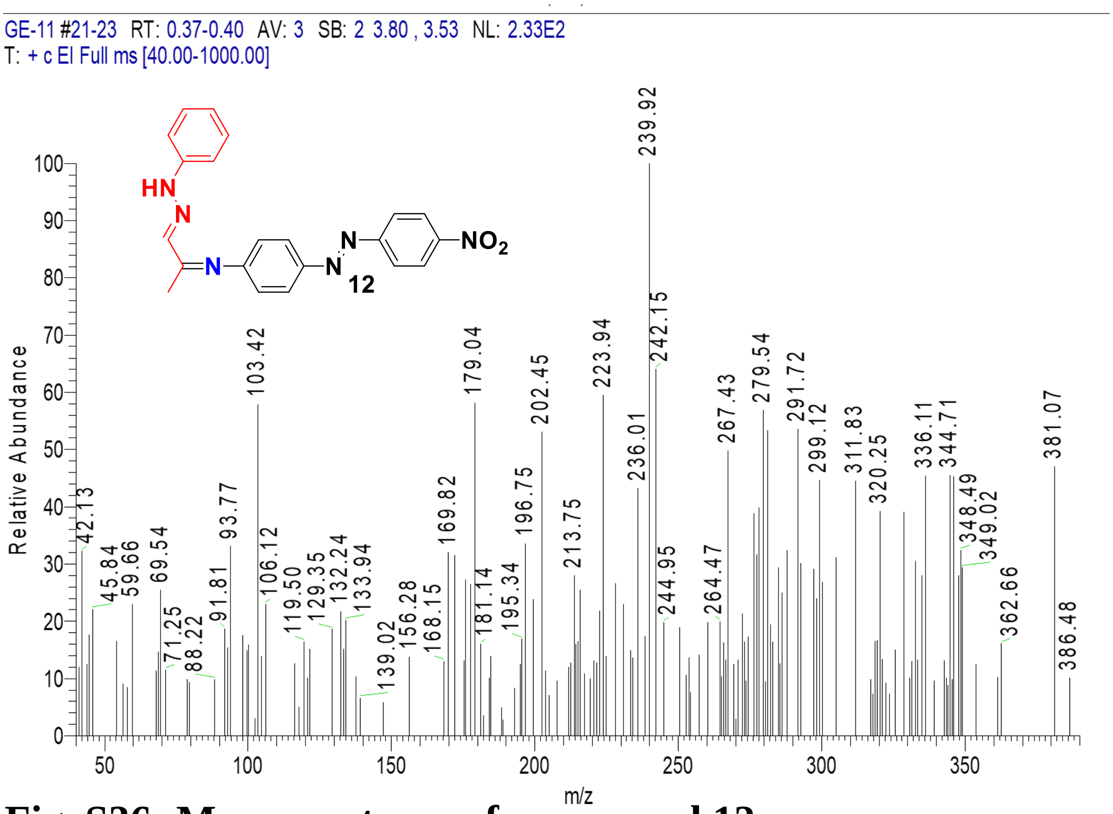

Fig. S36: Mass spectrum of compound 12

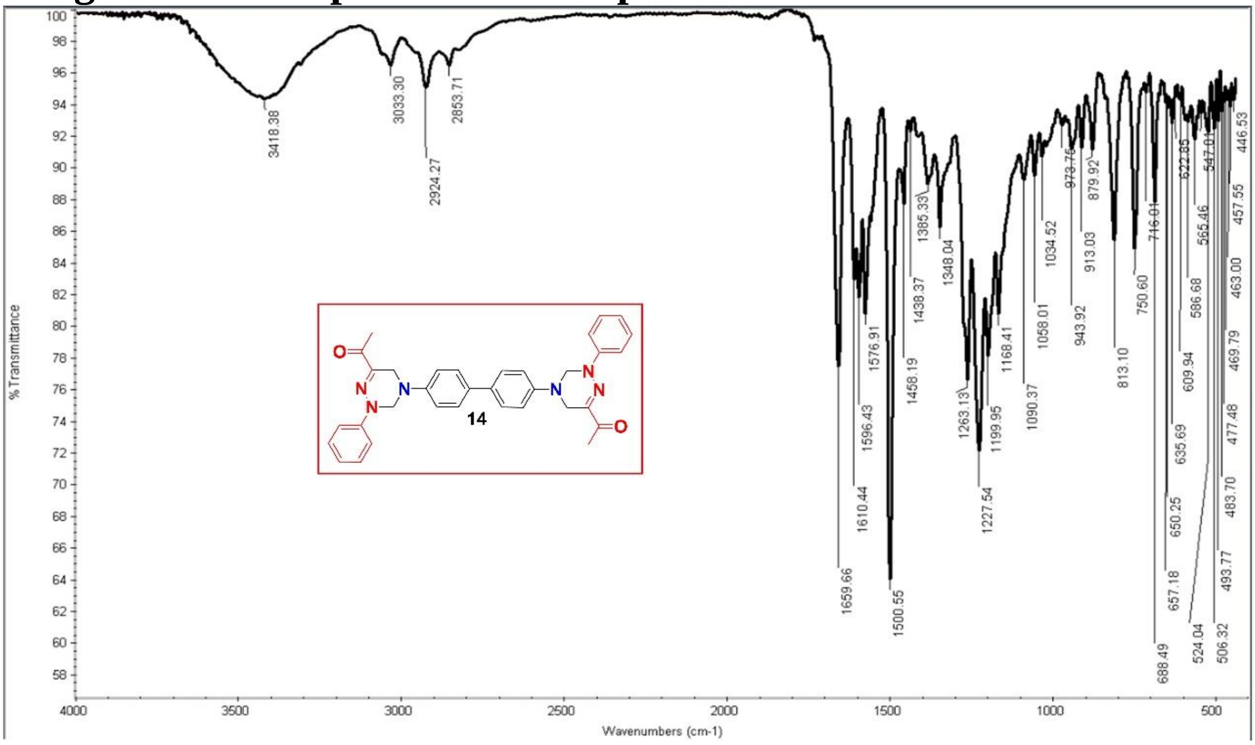

Fig. S37: IR spectrum of compound 14

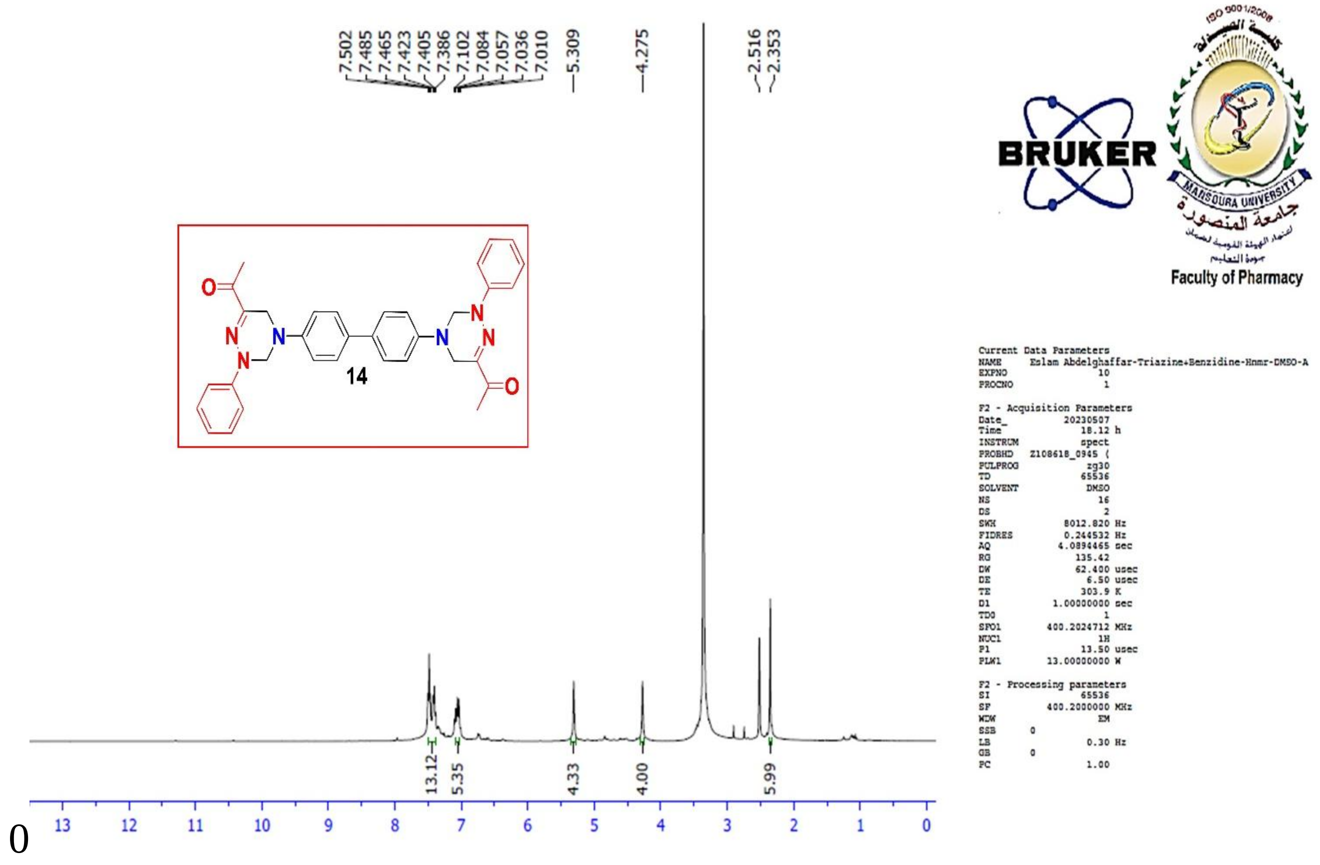

Fig. S38: ^{1}H-NMR spectrum of compound 14

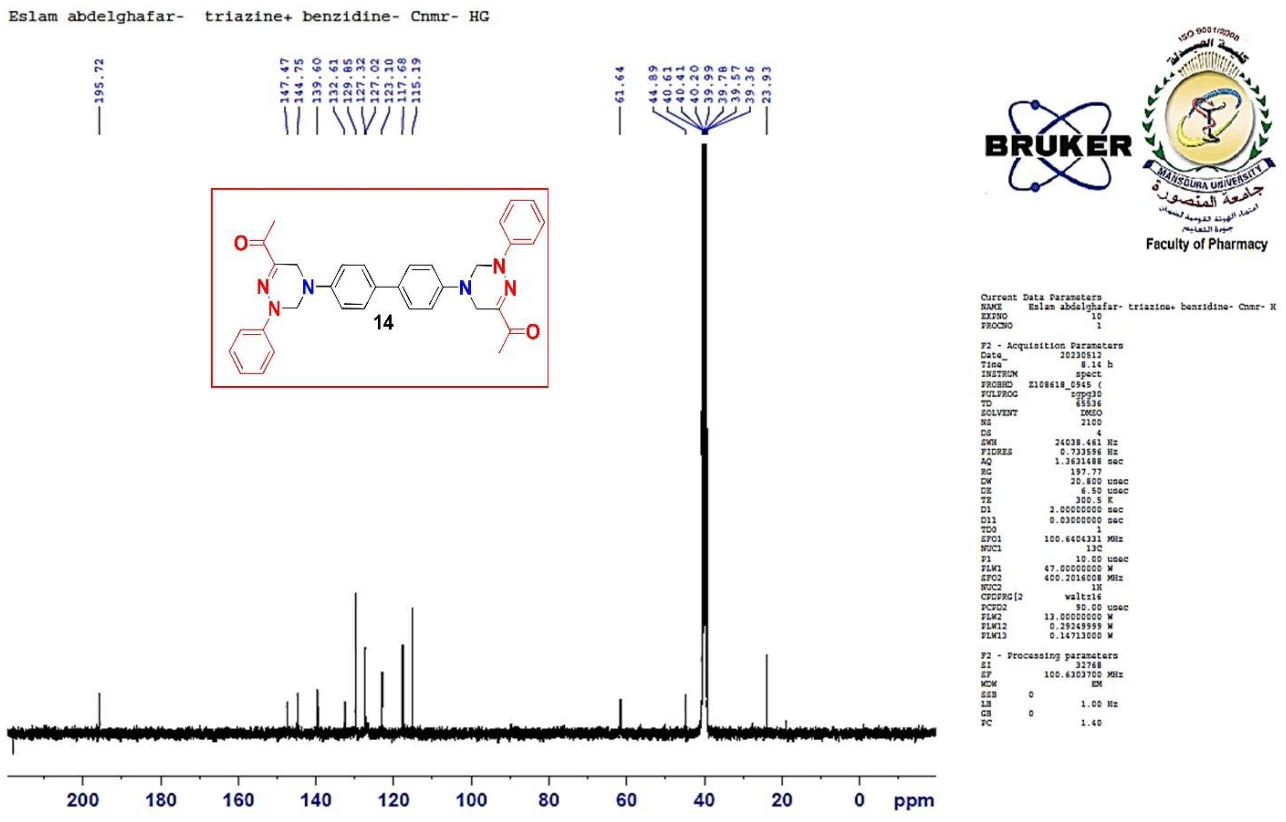

Fig. S39: ^{13}C-NMR spectrum of compound 14

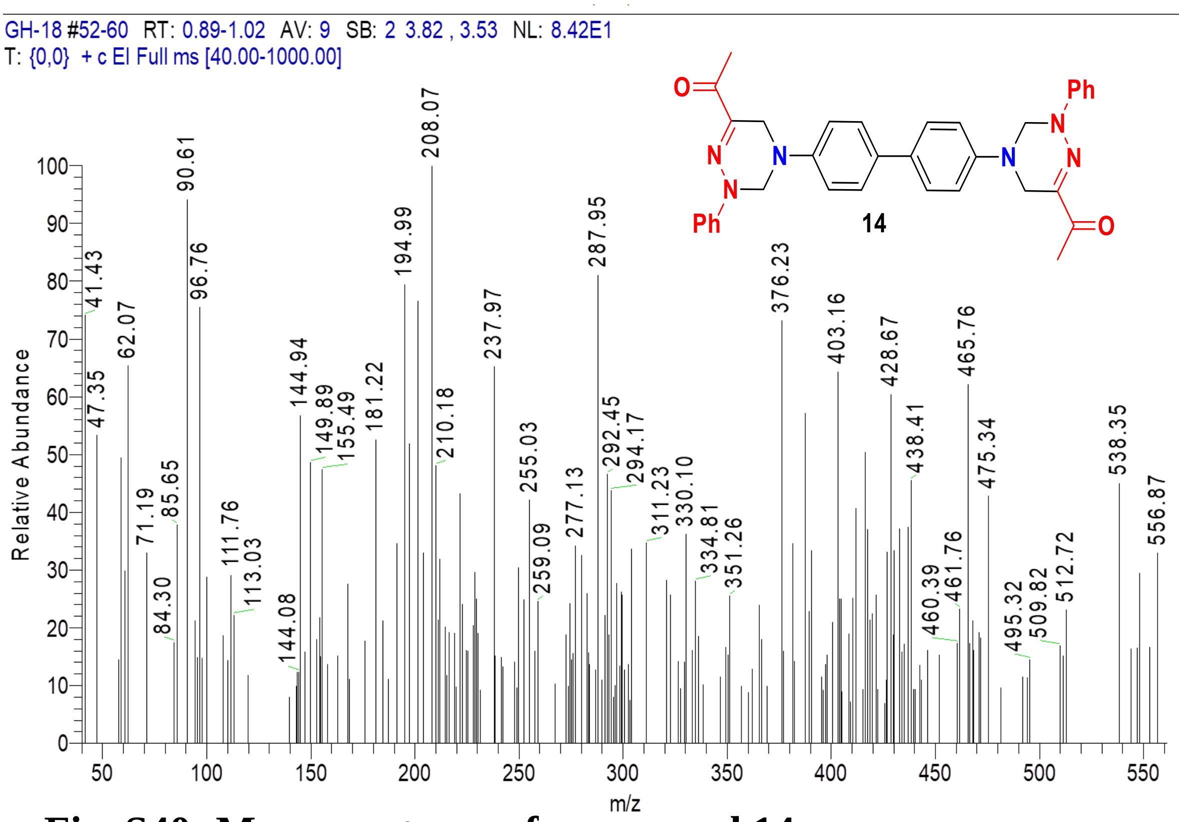

Fig. S40: Mass spectrum of compound 14

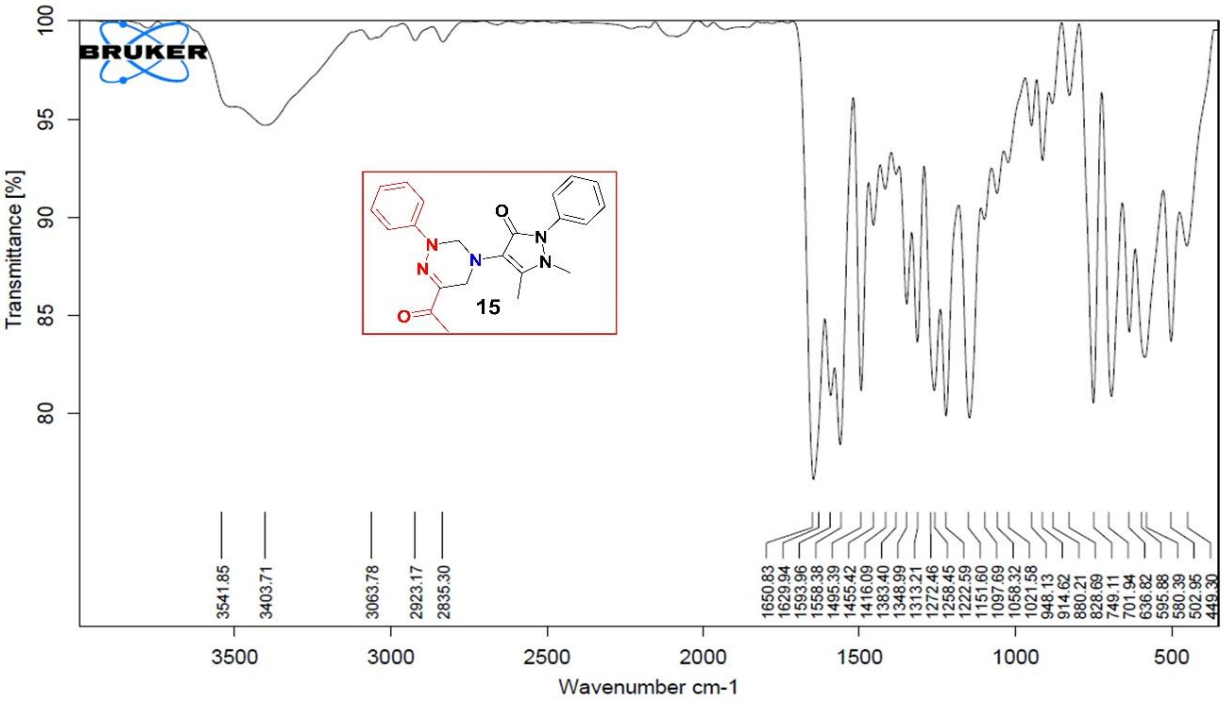

Fig. S41: IR spectrum of compound 15

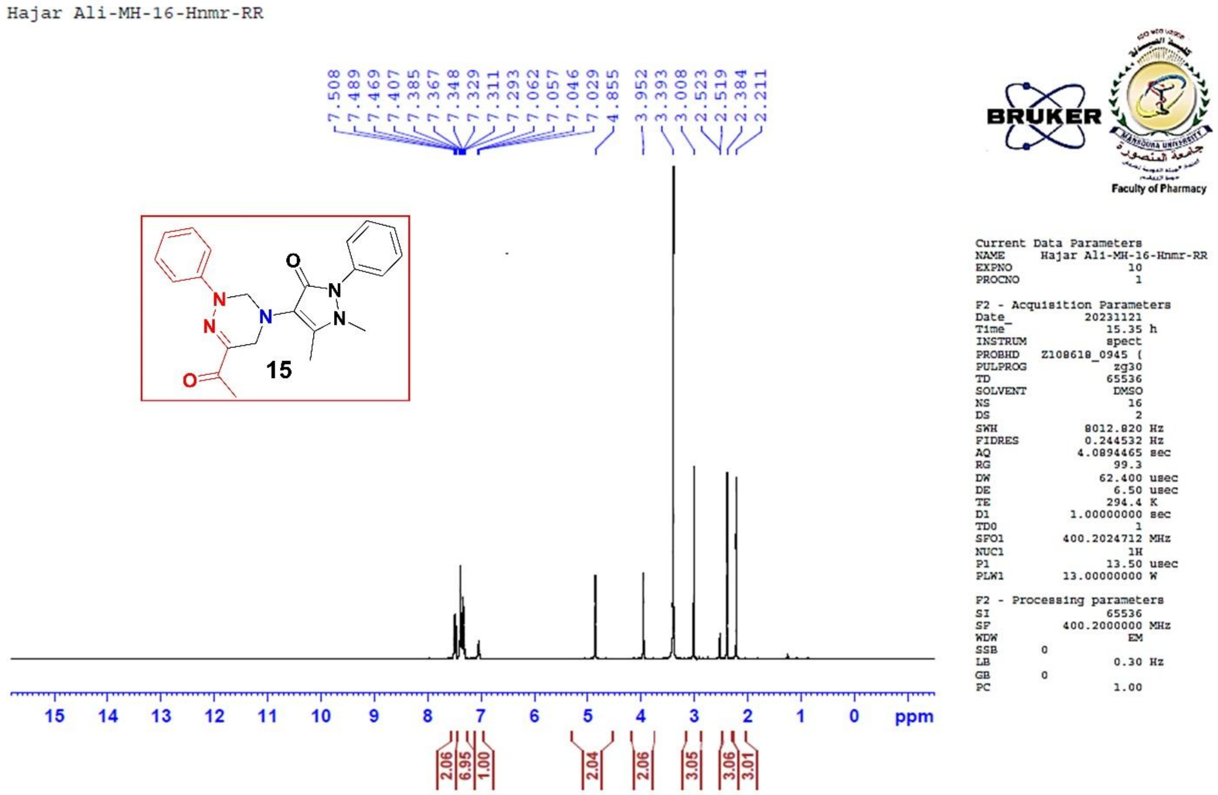

Fig. S42: ^{1}H-NMR spectrum of compound 15

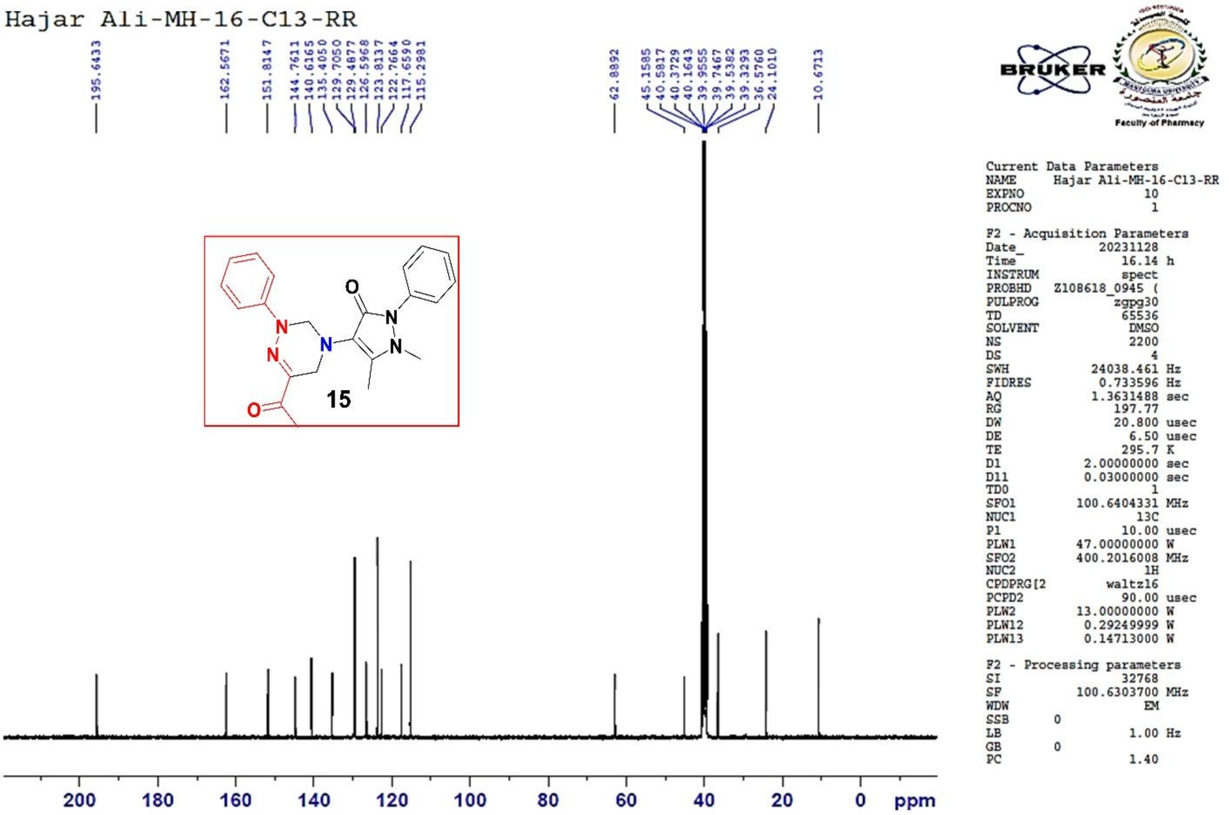

Fig. S43: ^{13}C-NMR spectrum of compound 15

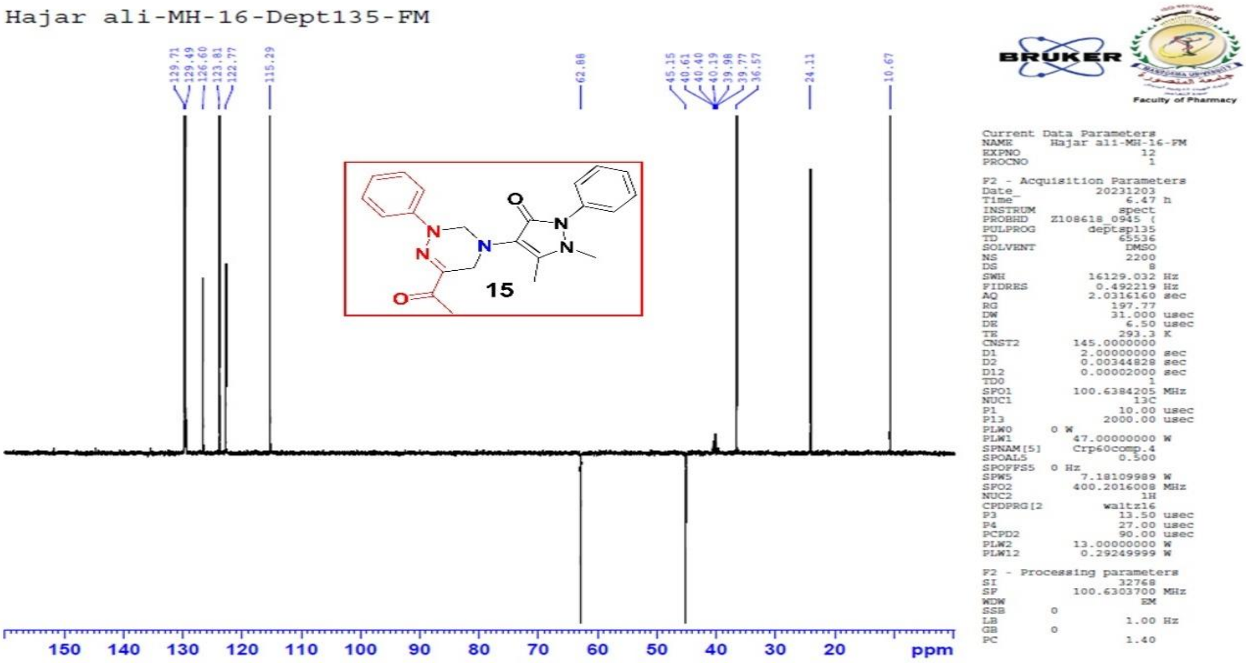

Fig. S44: DEPT (135) NMR spectrum of compound 15

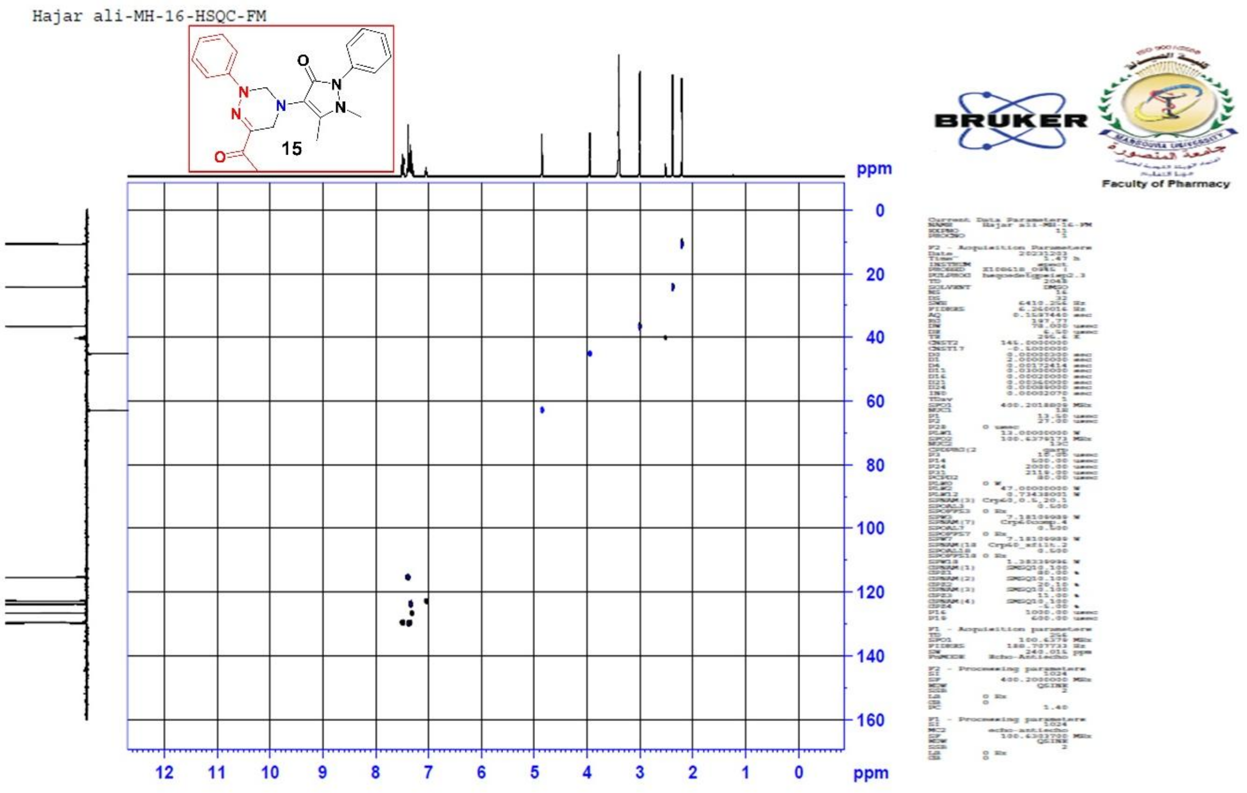

Fig. S45: HSQC spectrum of compound 15

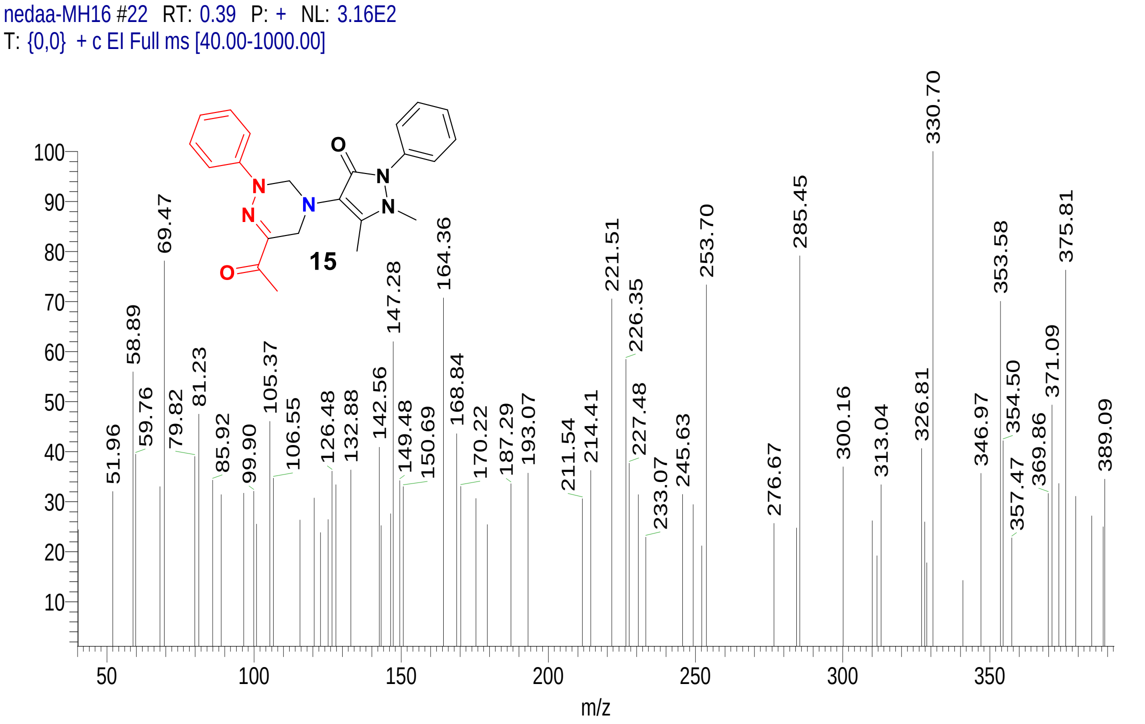

Fig. S46: Mass spectrum of compound 15

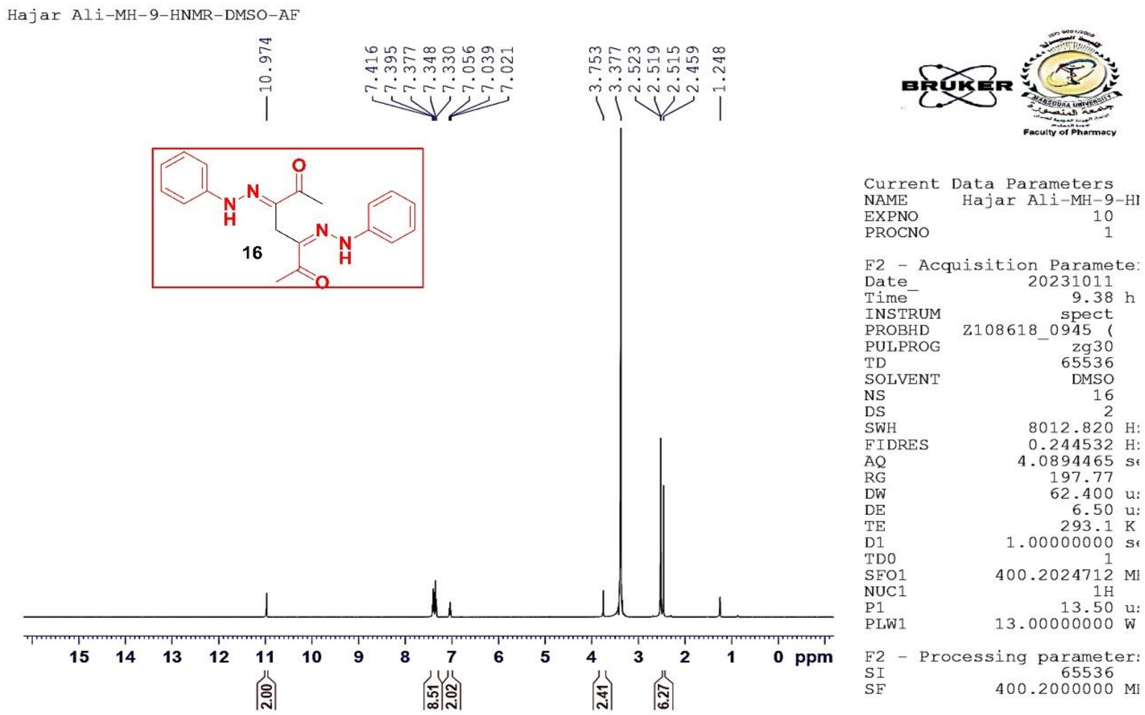

Fig. S47: ^{1}H-NMR spectrum of compound 16

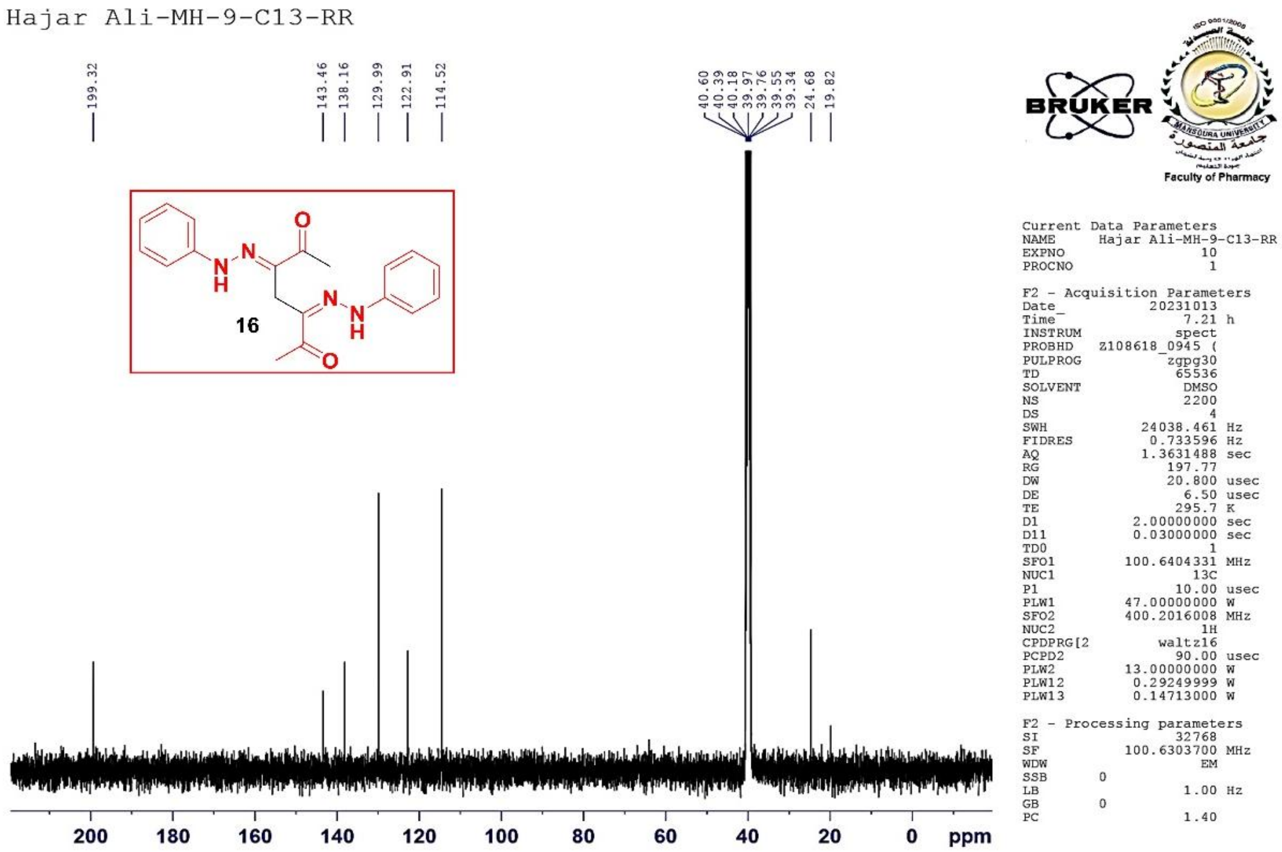

Fig. S48: ^{13}C-NMR spectrum of compound 16

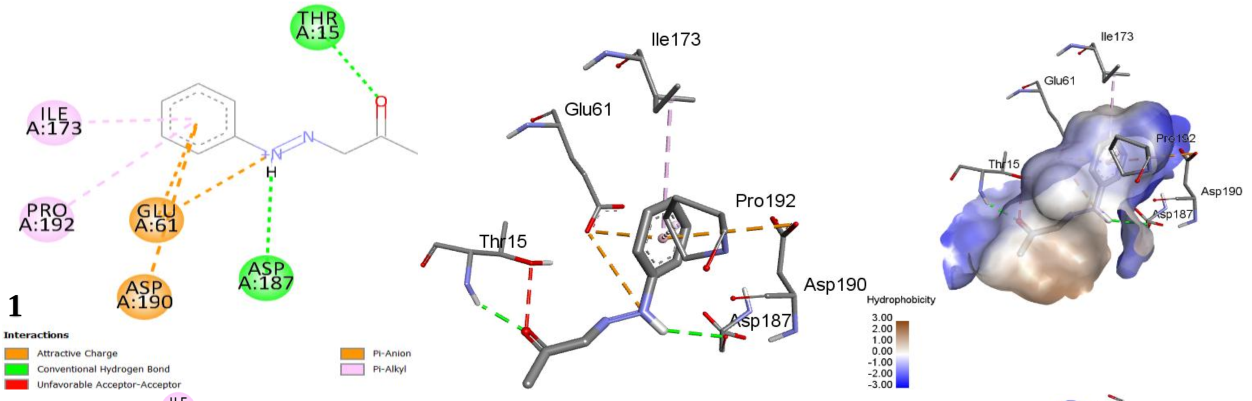

1

Interactions

- Attractive Charge
- Conventional Hydrogen Bond
- Unfavorable Acceptor-Acceptor
- Pi-Anion
- Pi-Alkyl

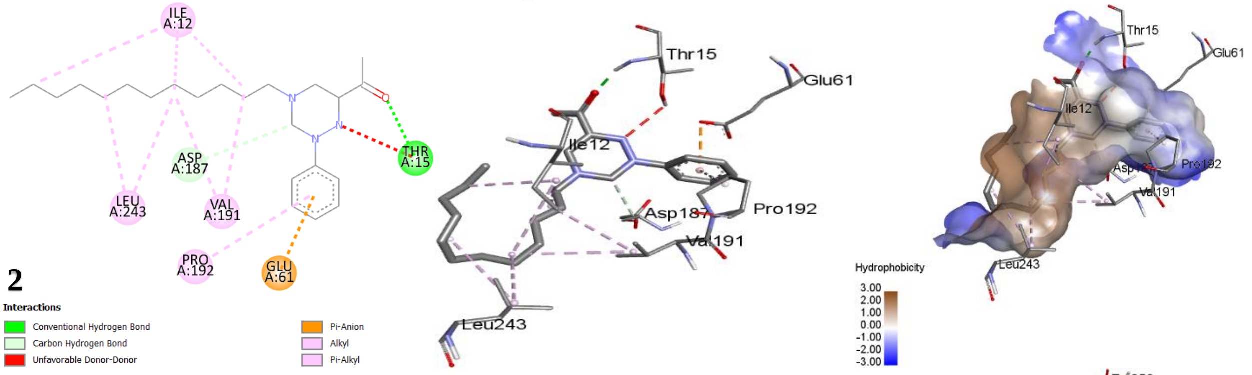

2

Interactions

- Conventional Hydrogen Bond
- Carbon Hydrogen Bond
- Unfavorable Donor-Donor
- Pi-Anion
- Alkyl
- Pi-Alkyl

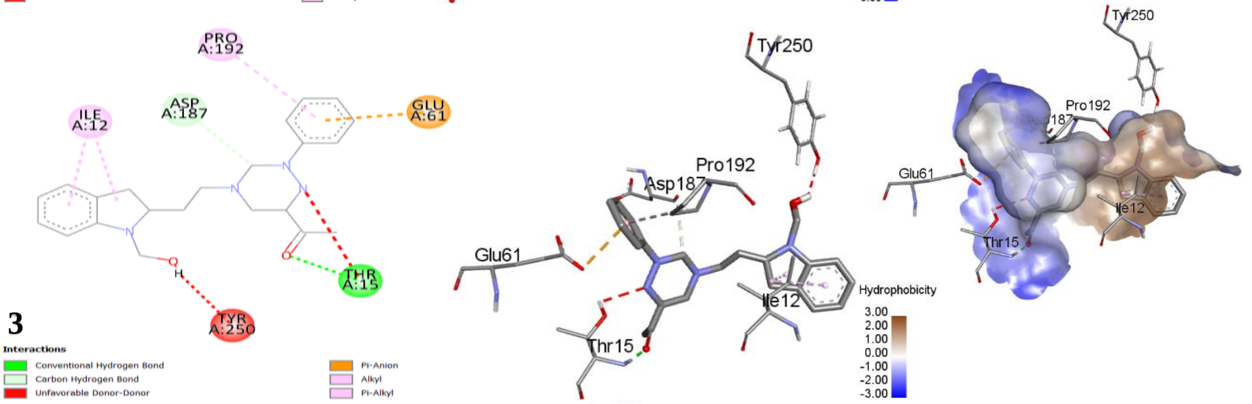

3

Interactions

- Conventional Hydrogen Bond
- Carbon Hydrogen Bond
- Unfavorable Donor-Donor
- Pi-Anion
- Alkyl
- Pi-Alkyl

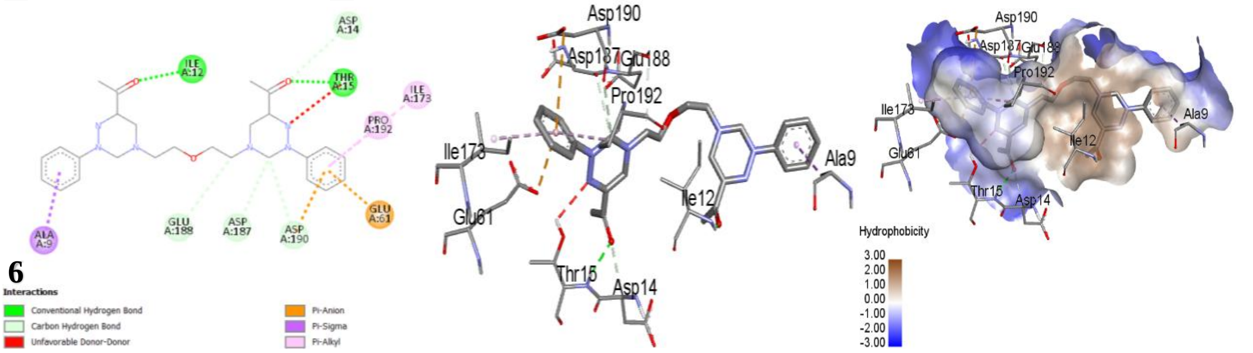

6

Interactions

- Conventional Hydrogen Bond
- Carbon Hydrogen Bond
- Unfavorable Donor-Donor
- Pi-Anion
- Pi-Sigma
- Pi-Alkyl

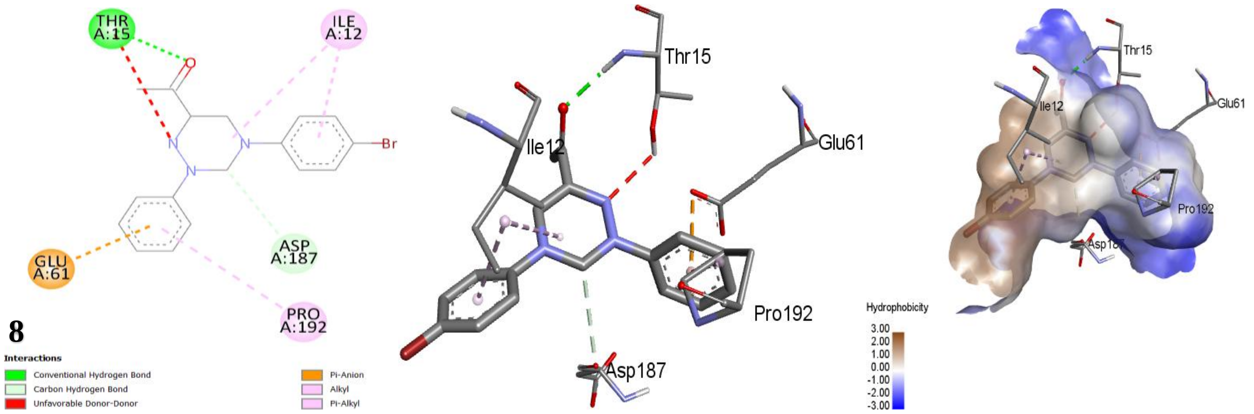
THR A:15
ILE A:12
Br
GLU A:61
ASP A:187
PRO A:192
Interactions
Conventional Hydrogen Bond
Carbon Hydrogen Bond
Unfavorable Donor-Donor
Pi-Anion
Alkyl
Pi-Alkyl
Thr15
Ile12
Glu61
Pro192
Asp187
Hydrophobicity
3.00
2.00
1.00
0.00
-1.00
-2.00
-3.00

8

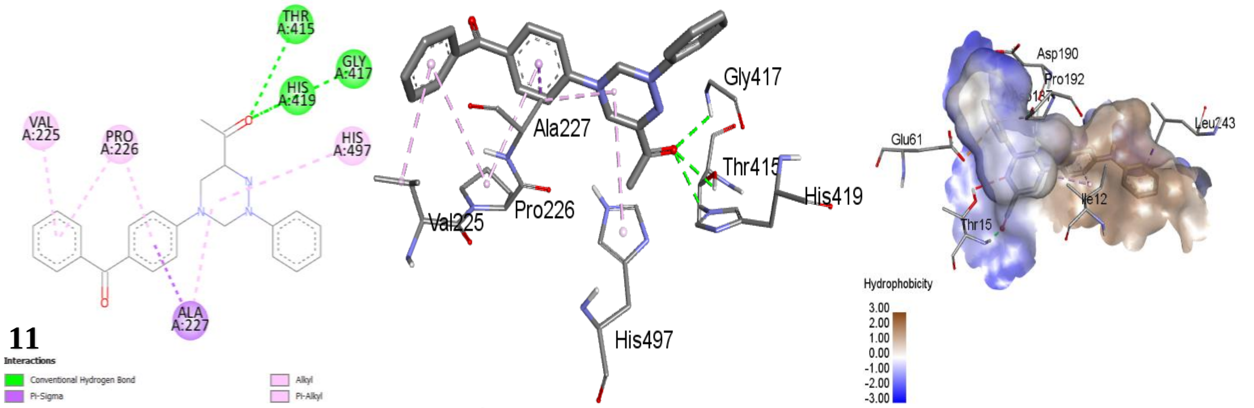
THR A:415
GLY A:417
HIS A:419
VAL A:225
PRO A:226
HIS A:497
ALA A:227
Interactions
Conventional Hydrogen Bond
Pi-Sigma
Alkyl
Pi-Alkyl
Gly417
Ala227
Thr415
His419
Val225
Pro226
His497
Asp190
Pro192
Glu61
Leu243
Ile12
Thr15
Hydrophobicity
3.00
2.00
1.00
0.00
-1.00
-2.00
-3.00

11

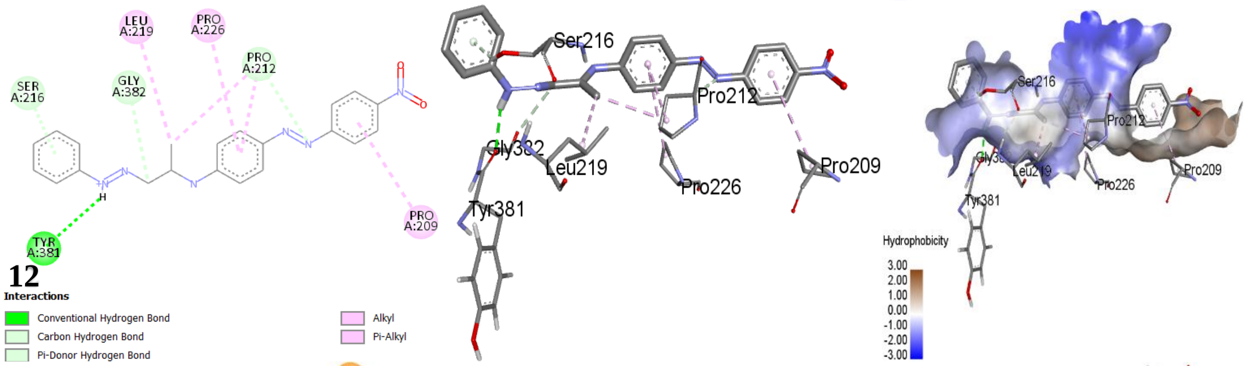
LEU A:219
PRO A:226
SER A:216
GLY A:382
PRO A:212
PRO A:209
TYR A:381
Interactions
Conventional Hydrogen Bond
Carbon Hydrogen Bond
Pi-Donor Hydrogen Bond
Alkyl
Pi-Alkyl
Ser216
Pro212
Gly382
Leu219
Pro226
Pro209
Tyr381
Hydrophobicity
3.00
2.00
1.00
0.00
-1.00
-2.00
-3.00

12

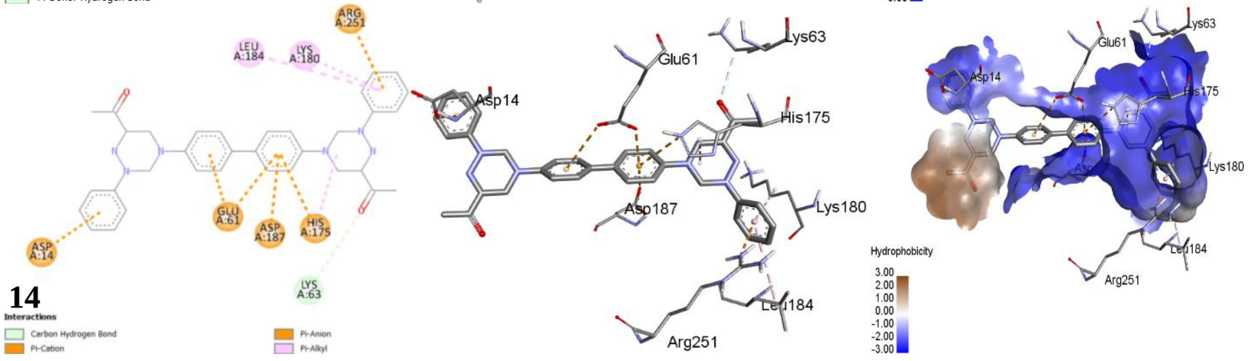
ARG A:251
LEU A:184
LYS A:180
GLU A:61
ASP A:187
HIS A:175
ASP A:14
LYS A:63
Interactions
Carbon Hydrogen Bond
Pi-Cation
Pi-Anion
Pi-Alkyl
Lys63
Glu61
Asp14
His175
Asp187
Lys180
Leu184
Arg251
Hydrophobicity
3.00
2.00
1.00
0.00
-1.00
-2.00
-3.00

14

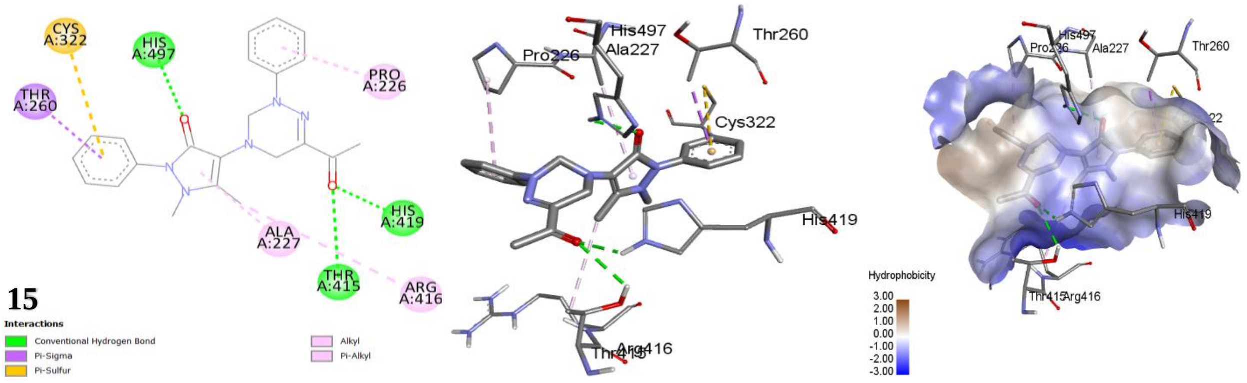

Fig. S49: **Depictions of the 2D, 3D structures, and hydrophobic views of tested compounds against 1OF0 protein.**

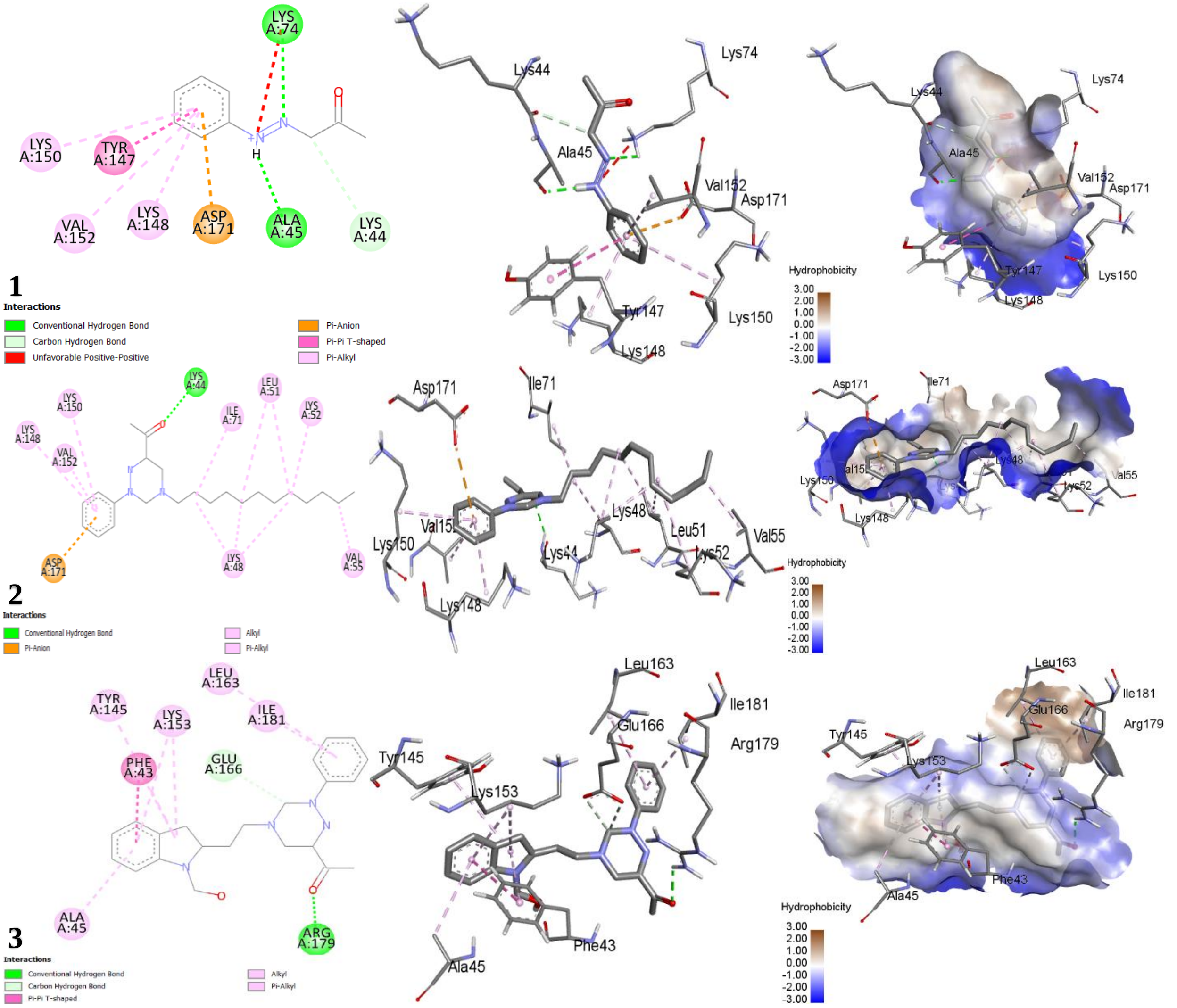

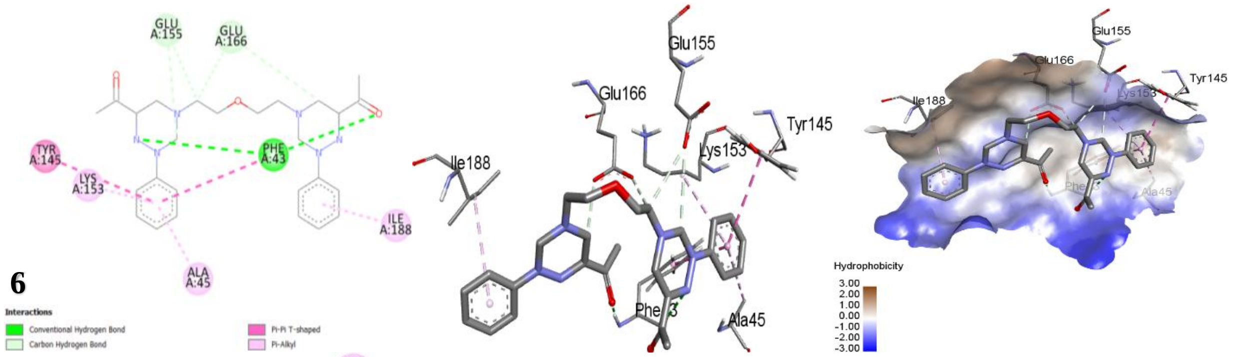
Interactions
Conventional Hydrogen Bond
Carbon Hydrogen Bond
Pi-Pi T-shaped
Pi-Alkyl
Hydrophobicity

6

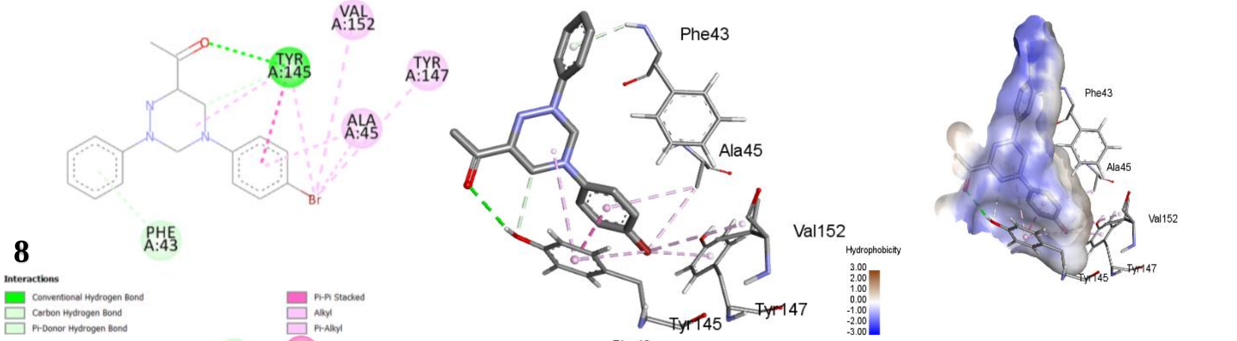
Interactions
Conventional Hydrogen Bond
Carbon Hydrogen Bond
Pi-Donor Hydrogen Bond
Pi-Pi Stacked
Alkyl
Pi-Alkyl
Hydrophobicity

8

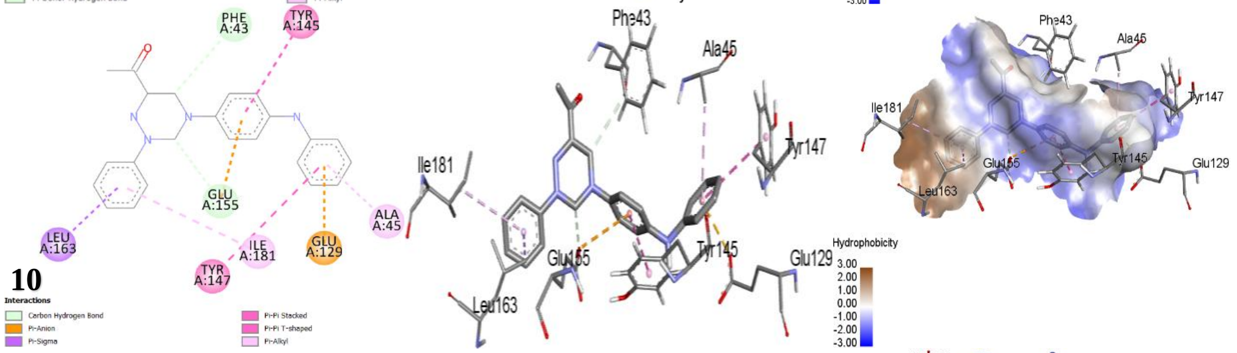
Interactions
Carbon Hydrogen Bond
Pi-Anion
Pi-Sigma
Pi-Pi Stacked
Pi-Pi T-shaped
Pi-Alkyl
Hydrophobicity

10

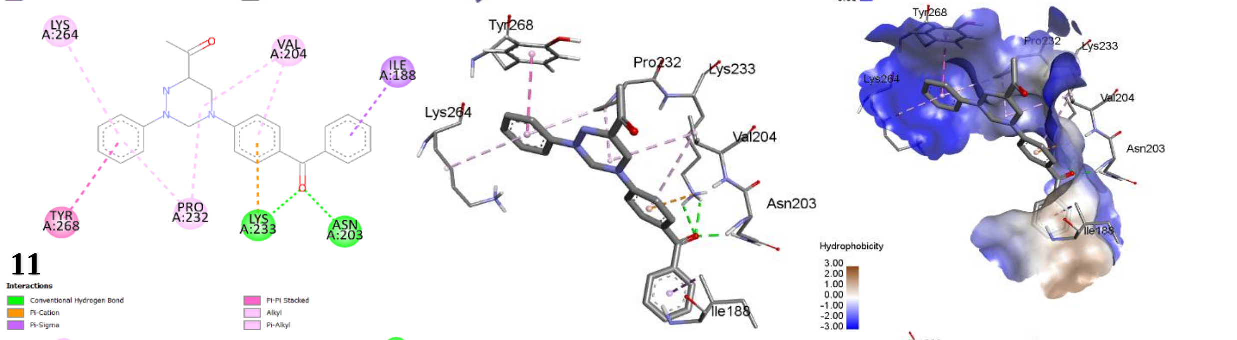
Interactions
Conventional Hydrogen Bond
Pi-Cation
Pi-Sigma
Pi-Pi Stacked
Alkyl
Pi-Alkyl
Hydrophobicity

11

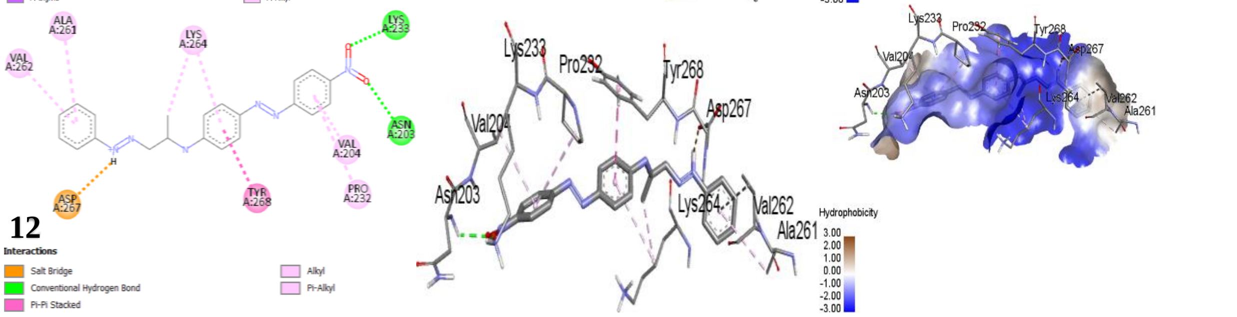
Interactions
Salt Bridge
Conventional Hydrogen Bond
Pi-Pi Stacked
Alkyl
Pi-Alkyl
Hydrophobicity

12

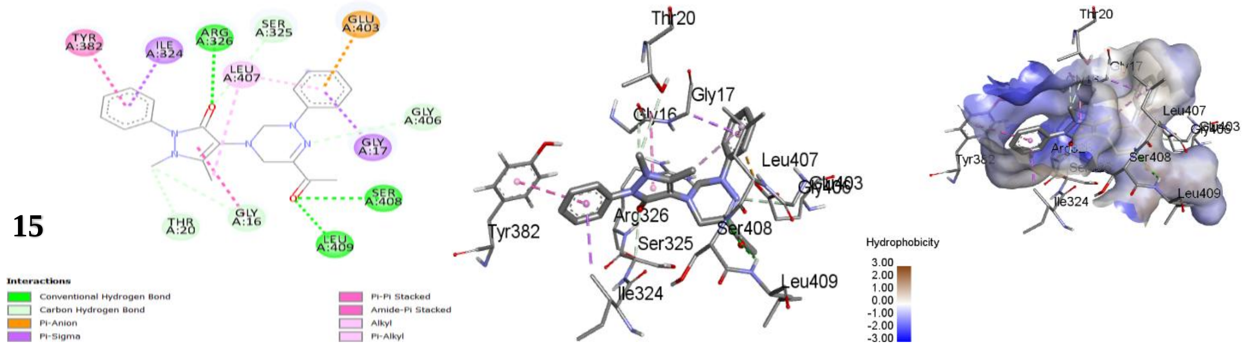

15

Fig. S50: **Binding mode as well as visual interaction of all synthesized compounds with the binding active site of 8P20 protein.**

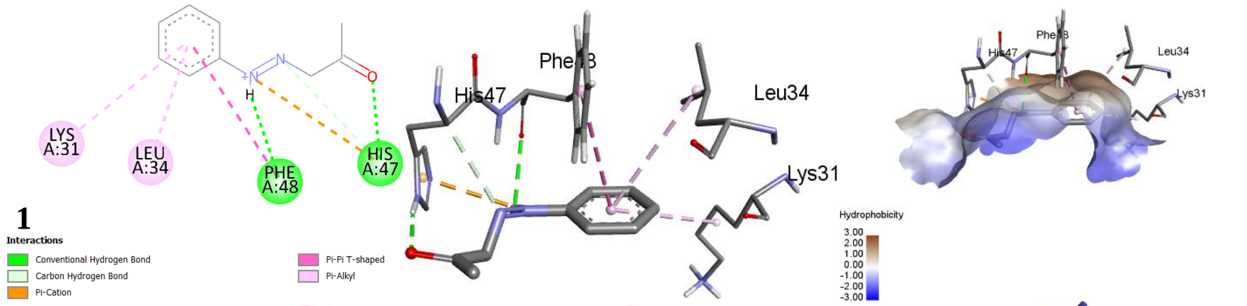

1

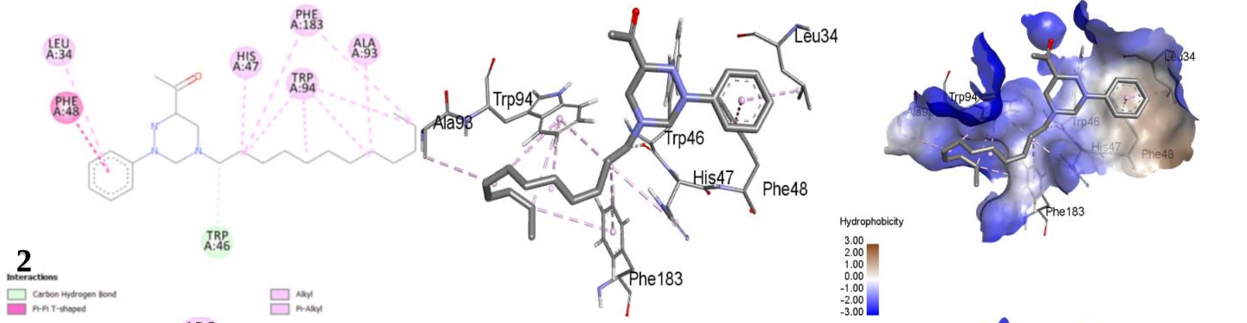

2

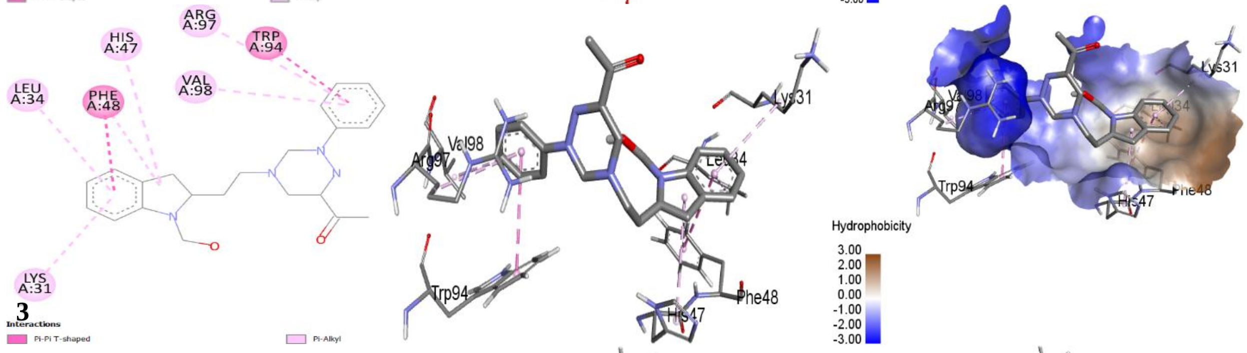

3

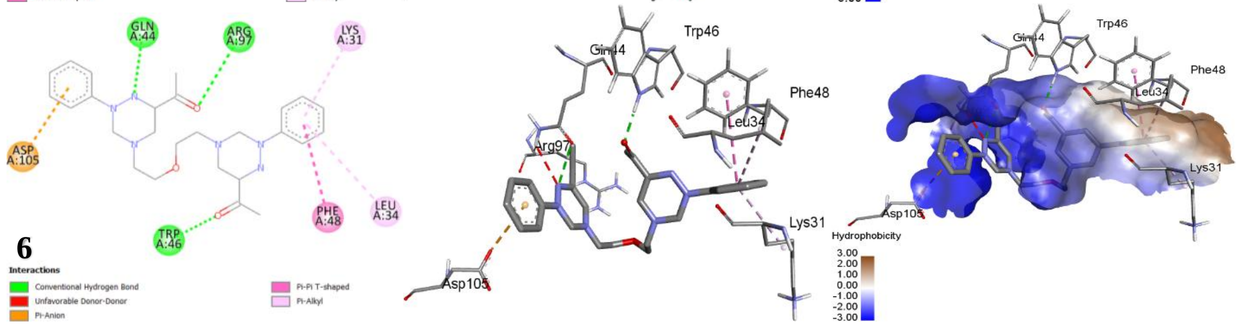

6

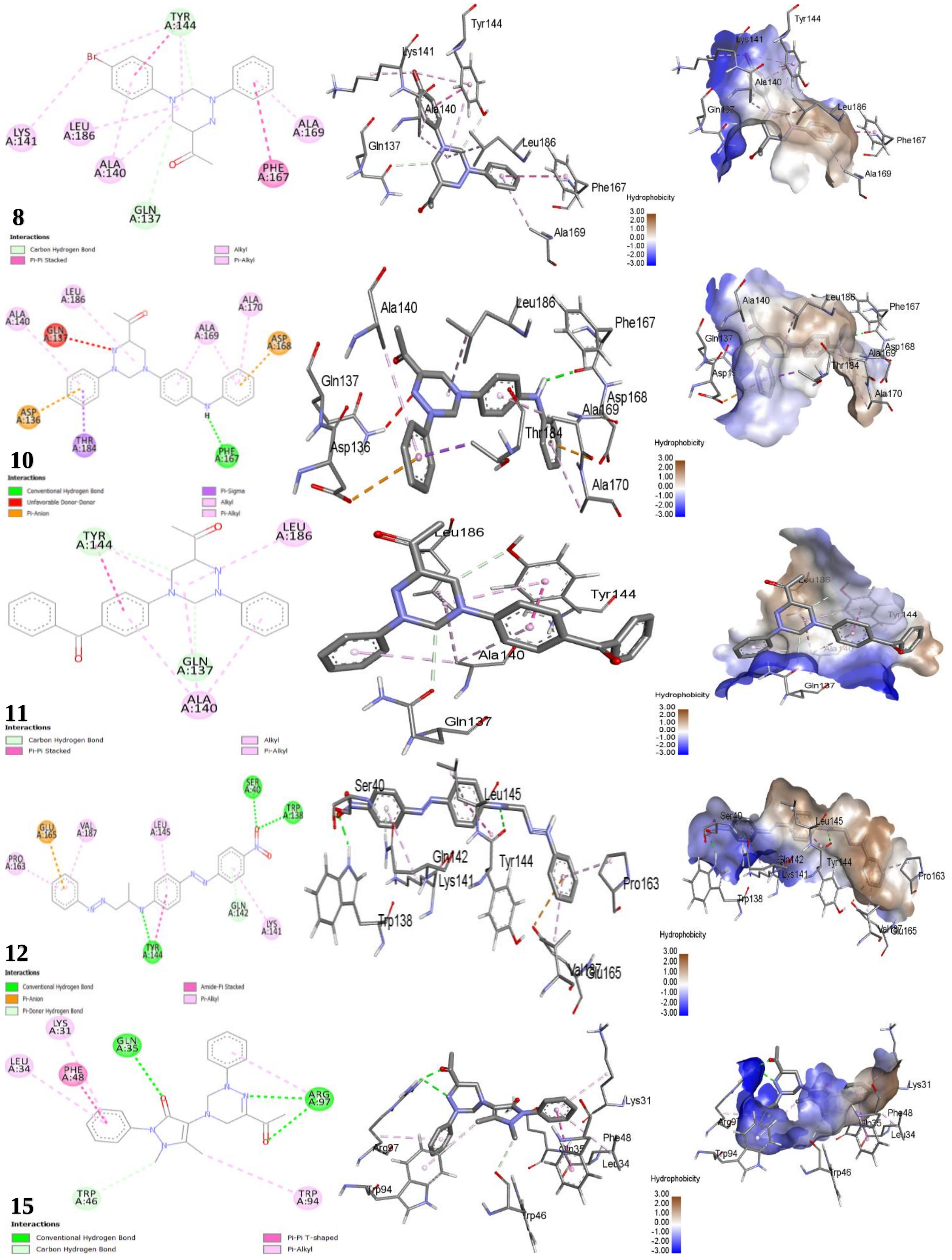

Fig. S51: **Molecular docking images of the inhibitors with 1KQB protein.**

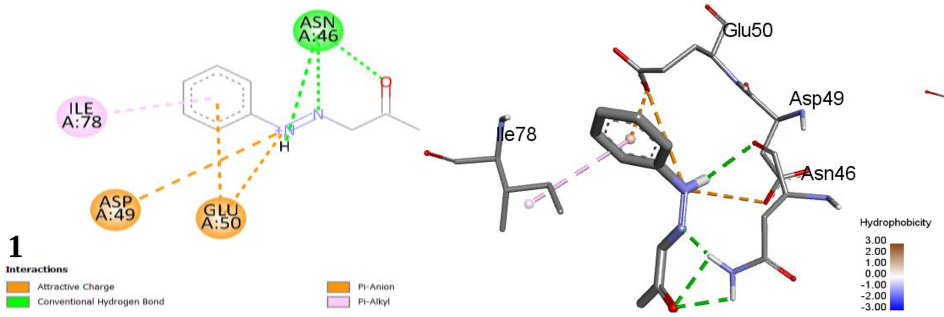
ASN A:46
ILE A:78
ASP A:49
GLU A:50
1
Interactions
Attractive Charge
Conventional Hydrogen Bond
Pi-Anion
Pi-Alkyl
Glu50
Asp49
Ile78
Asn46
Hydrophobicity
3.00
2.00
1.00
0.00
-1.00
-2.00
-3.00

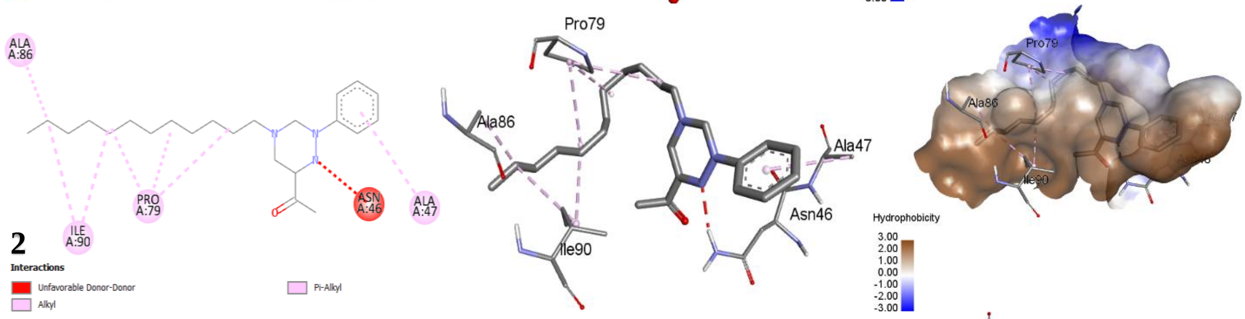
ALA A:86
PRO A:79
ILE A:90
ASN A:46
ALA A:47
2
Interactions
Unfavorable Donor-Donor
Alkyl
Pi-Alkyl
Pro79
Ala86
Ala47
Asn46
Ile90
Hydrophobicity
3.00
2.00
1.00
0.00
-1.00
-2.00
-3.00

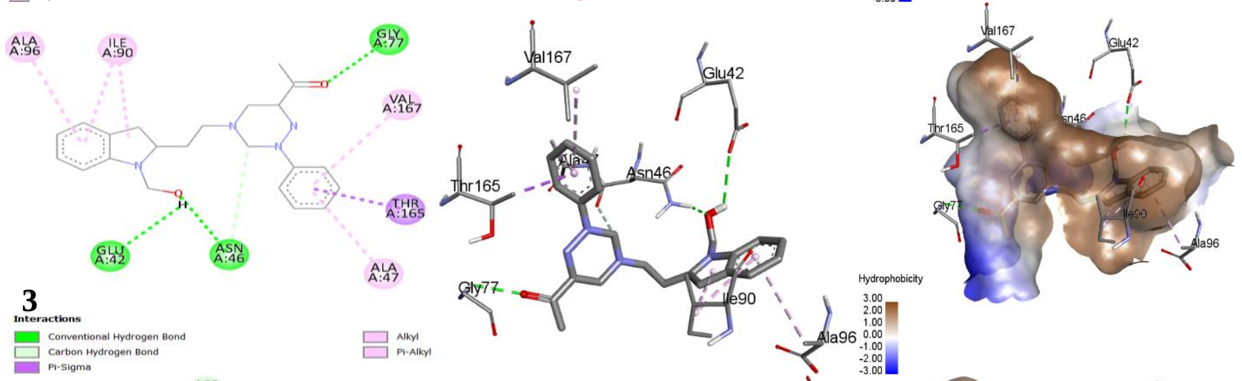
ALA A:96
ILE A:90
GLY A:77
VAL A:167
THR A:165
GLU A:42
ASN A:46
ALA A:47
3
Interactions
Conventional Hydrogen Bond
Carbon Hydrogen Bond
Pi-Sigma
Alkyl
Pi-Alkyl
Val167
Glu42
Thr165
Asn46
Gly77
Ile90
Ala96
Hydrophobicity
3.00
2.00
1.00
0.00
-1.00
-2.00
-3.00

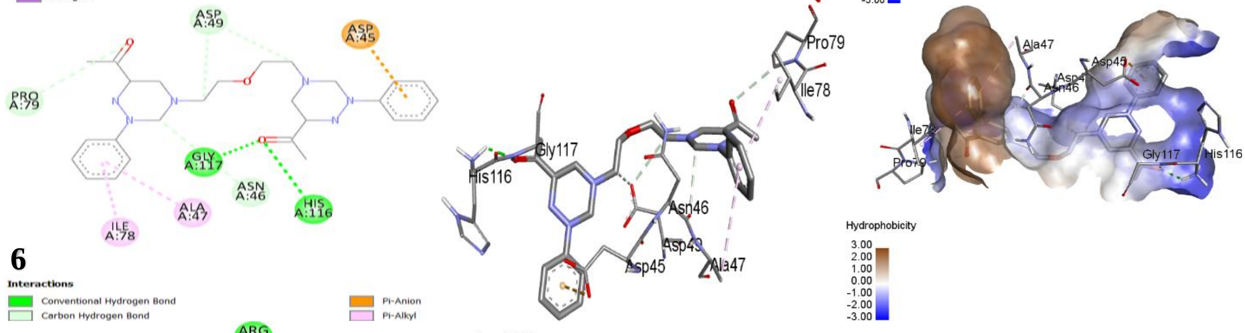
ASP A:49
ASP A:45
PRO A:79
GLY A:117
ASN A:46
HIS A:116
ALA A:47
ILE A:78
6
Interactions
Conventional Hydrogen Bond
Carbon Hydrogen Bond
Pi-Anion
Pi-Alkyl
Pro79
Ile78
Gly117
His116
Asn46
Asp49
Asp45
Ala47
Hydrophobicity
3.00
2.00
1.00
0.00
-1.00
-2.00
-3.00

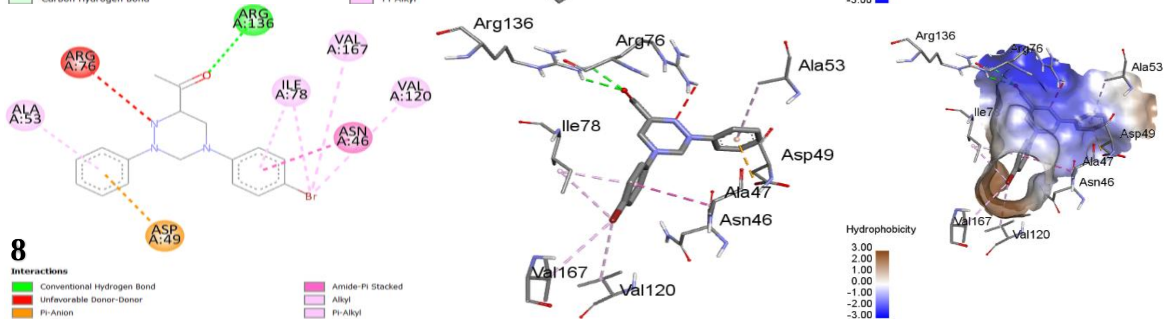
ARG A:136
ARG A:76
VAL A:167
ILE A:78
VAL A:120
ALA A:53
ASN A:46
ASP A:49
Br
8
Interactions
Conventional Hydrogen Bond
Unfavorable Donor-Donor
Pi-Anion
Amide-Pi Stacked
Alkyl
Pi-Alkyl
Arg136
Arg76
Ala53
Ile78
Asp49
Ala47
Asn46
Val167
Val120
Hydrophobicity
3.00
2.00
1.00
0.00
-1.00
-2.00
-3.00

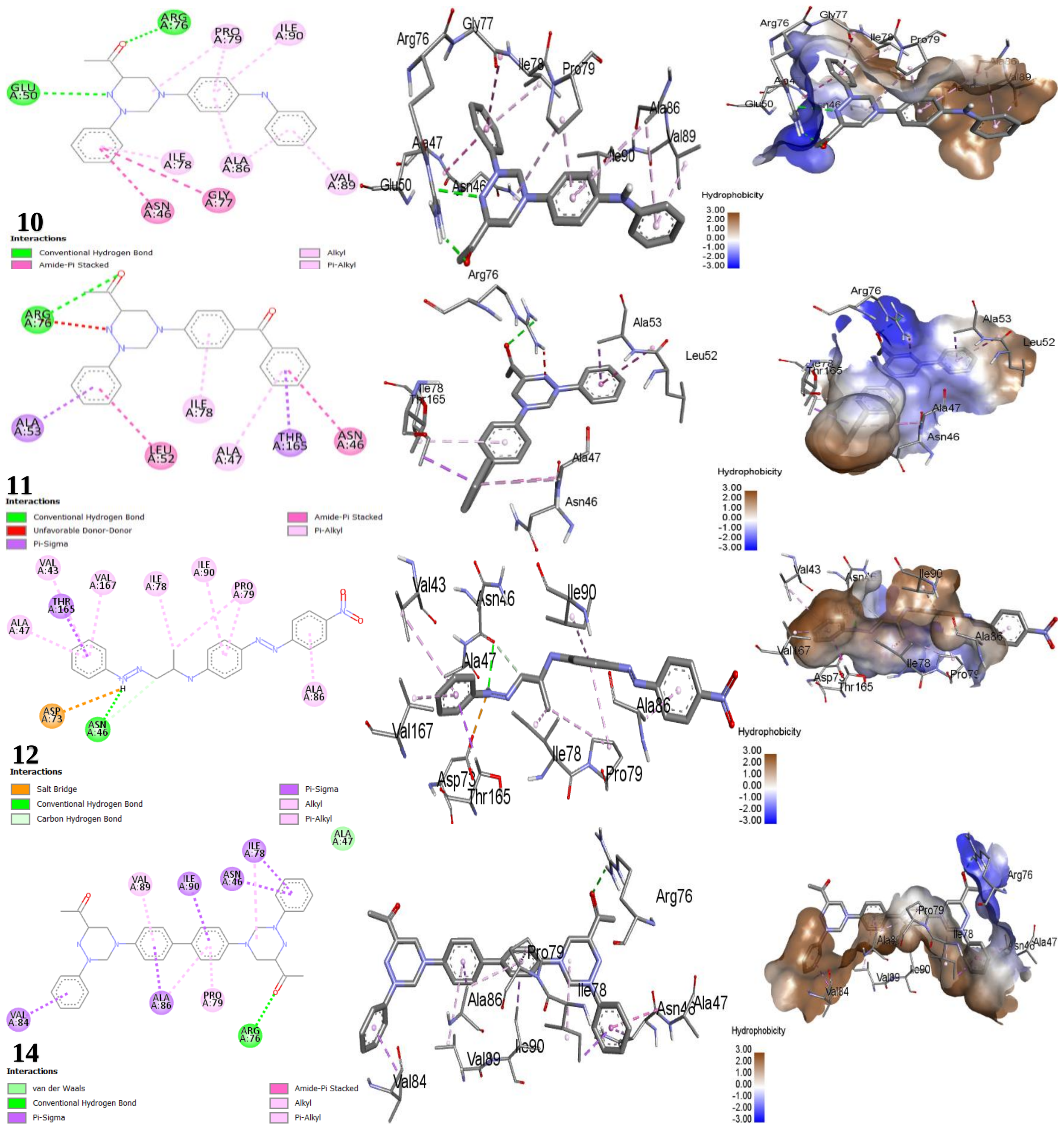

Fig. S52: Interaction modes of synthesized compounds with *E. coli* protein (PDB: 1KZN).

<u>Method used for antibacterial analysis:</u>

Agar well diffusion method:

The agar plate surface is inoculated by spreading a volume of the microbial inoculum over the entire agar surface. Then, a hole with a diameter of 9 mm is punched aseptically with a sterile corn borer or a tip, and a volume (100 µL) of sample at desired concentration is introduced into the well. Then, agar plates are incubated under suitable conditions depending upon the test microorganism. The antimicrobial agent diffuses in the agar medium and inhibits the growth of the microbial strain tested [1,2].

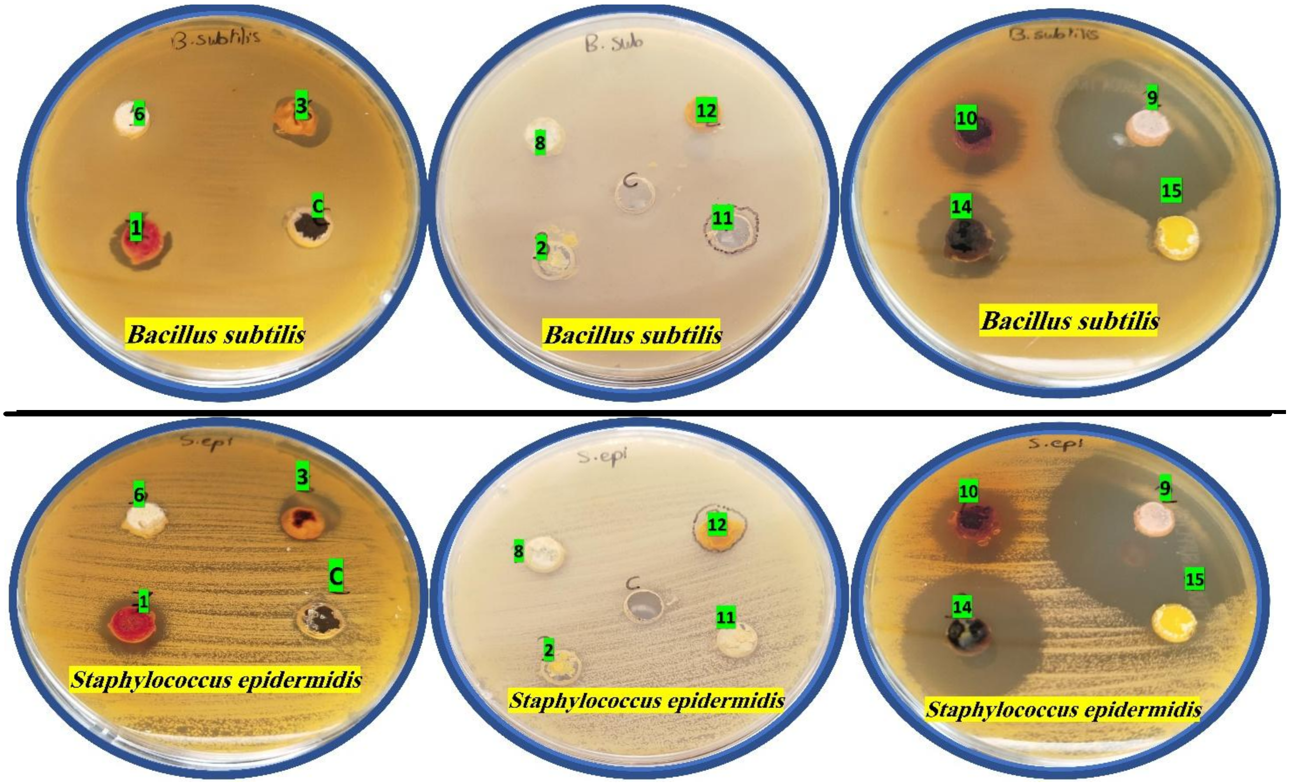

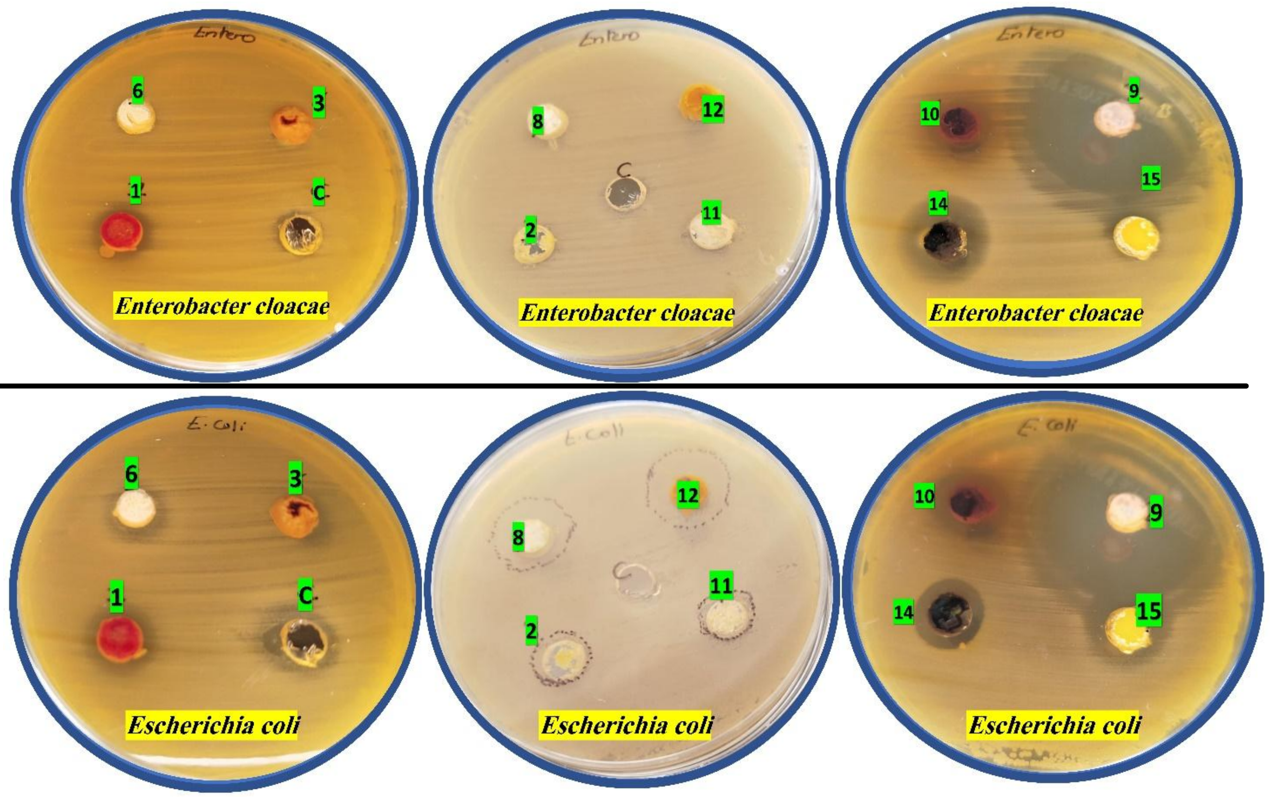

Fig. S53: Inhibition zone diameter for the designed compounds.

1. S. Magaldi, S. Mata-Essayag, C. H. De Capriles, C. Pérez, M. T. Colella, C. Olaizola and Y. Ontiveros, *Int. J. Infect. Dis.*, 2004, **8**, 39–45.

2. C. Valgas, S. M. de Souza, E. F. A. Smânia and A. Smânia Jr, *Braz. J. Microbiol.*, 2007, **38**, 369–380.
